# Toward Multi(radio)metalated DNA: Enzymatic Polymerization of Metal‐Chelate‐Modified Deoxyribonucleoside Triphosphates

**DOI:** 10.1002/anie.1429706

**Published:** 2026-05-07

**Authors:** Antonio A. W. L. Wong, François Bénard, David M. Perrin

**Affiliations:** ^1^ Department of Basic and Translational Research BC Cancer Research Institute Vancouver BC Canada; ^2^ Department of Chemistry University of British Columbia Vancouver BC Canada; ^3^ Department of Molecular Imaging and Therapy BC Cancer Agency Vancouver BC Canada; ^4^ Department of Radiology University of British Columbia Vancouver BC Canada

**Keywords:** chemical biology, chemoenzymatic synthesis, nuclear medicine, nucleic acid modifications, radiopharmaceuticals

## Abstract

We report the first enzymatic synthesis of precisely defined metal‐oligonucleotide conjugates through template‐directed polymerase‐mediated primer extension with two different (radio)metal‐chelator‐modified deoxyribonucleoside triphosphates. We successfully incorporated five different metals (Ga, In, Tb, Lu, and Y) along designed oligonucleotide templates, including the simultaneous incorporation of two different metal chelates within a given strand. To provide bio‐orthogonality, this approach exploits the well‐known enzymatic fidelity of DNA polymerase to generate uniformly metal‐loaded DNA constructs with well‐defined and programmable compositions. The platform is validated using chelators DOTA and DTPA and confirmed by both inductively coupled plasma mass spectrometry (ICP‐MS) quantification and autoradiographic analysis following gel electrophoresis to demonstrate radioisotope incorporation with clinically relevant metals (^68^Ga for PET, ^111^In for SPECT, ^161^Tb and ^177^Lu for β^−^ therapy), establishing the potential for using the information content of DNA to create multi‐metalated radiopharmaceuticals. This programmable platform establishes a new paradigm for metalated molecular probe development with versatility for potential therapeutic, theranostic, and mass spectrometry‐based applications, and potentially generalizable to other metal isotopes based on diagnostic or therapeutic requirements.

## Graphical abstract

This work presents the first enzymatic polymerization of defined metal‐chelator‐modified nucleotides that leverage polymerase fidelity for uniform (radio)metal loading with successful incorporation of five different metals: Ga, In, Tb, Lu, and Y. Autoradiography validates radiometal incorporation whereas ICP‐MS is used to quantify multi‐metal incorporation establishing a versatile platform for theranostic development and for bioanalytical applications.

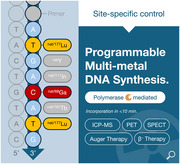

## 1 Introduction

Chelated metals empower a broad range of biomedical and bioanalytical applications. In precision medicine, bioconjugates to chelated radiometals (e.g., ^68^Ga, ^90^Y, ^111^In, ^177^Lu, ^225^Ac) target radiotherapy to tumors, thereby improving patient outcomes, even offering long‐term remission [1, 2]. With imaging mass cytometry, antibodies conjugated to polymer‐bearing mass‐encoded metal‐chelates with different isotopic distributions drive multiplexed tissue profiling via time‐of‐flight cytometry (CyTOF) [3].

In parallel, synthetic nucleic acids underpin multiple biomedical applications [4], especially in in vitro diagnostics and genome sequencing [5, 6, 7, 8, 9]. Nucleic acids find roles as radio‐theranostics with selected aptamers serving as stand‐alone precision agents [10, 11, 12] and in pre‐targeting applications, where radiolabeled oligonucleotides hybridize to a complementary DNA strand conjugated to an antibody that has already associated with its target in vivo [13, 14].

**FIGURE 1:**
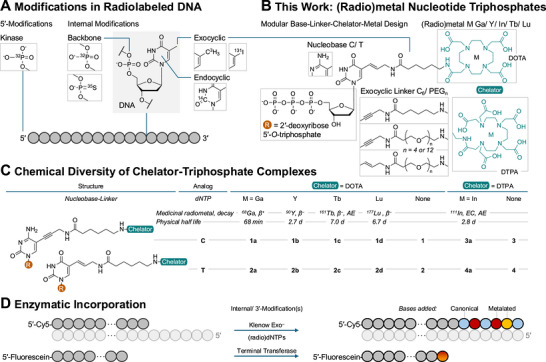
Methodologies of radiolabeling oligonucleotides. (A) Historical approaches to radiolabeling: 5′‐terminal labeling through radiochemistry or kinase activity (^32^P), or internally by enzymatic polymerization of radiolabeled triphosphates (^3^H, ^14^C, ^32^P, ^35^S, or ^131^I). (B) This work: (radio)metalated deoxynucleotide triphosphates (dYTPs) as modified dCTP (C) and dTTP (T) analogs. (C) An array of metalated dNTPs **1a–d**, **2a–d**, **3a,** and **4a** complexed with Ga, Y, In, Tb, and Lu, shown with radioactive decay modes (β^+^, positron decay; β^–^, electron decay; AE, Auger electron decay; EC, electron capture), and half‐lives (see Supporting Information). (D) dYTPs are substrates for Klenow Exo^– ^polymerase or terminal deoxynucleotidyl transferase (TdT). Grey circles represent commercial oligonucleotides, blue circles present canonical bases, and circles in red or yellow represent metalated bases.

Traditionally, nucleic acids have been radiolabeled enzymatically by incorporating deoxyribonucleotide triphosphates (dNTPs), bearing radioisotopes such as ^3^H, ^14^C, ^35^S, ^32^P, and ^131^I (Figure 1A). Nonradioactive labeling approaches have exploited fluorophore‐conjugated dNTPs [15] for sequencing applications, such as in situ imaging (DNA‐PAINT) [16, 17]. A few recent examples have employed stable metal chelates on base‐modified triphosphates for biophysical applications: these include a fluorescent Eu^3+^‐cryptate [18] and redox‐active Ru(bipy)_2_‐labeled deoxyribouridine triphosphates (dUTPs) [19], the simultaneous incorporation of two different pyrimidine deoxyribonucleotide triphosphates (dYTPs) bearing [Ru(bpy)_3_]^2+^ and [Os(bpy)_3_]^2+^ for luminescent and electrochemical detection [20], and the use of ferrocenyl‐modified nucleotides for potentiometric applications [21]. More recently, photoactivatable aptamers have been selected using dUTPs modified with Ru‐chelates [22].

To date, however, strategies for incorporating (radio)metal chelates into oligonucleotides have relied on standard bioconjugation approaches, which constrain stoichiometric control and hinder the assembly of multi‐metalated constructs. Most commonly, oligonucleotides are 5′‐conjugated to a single chelator, which is subsequently metalated with a luminescent metal (e.g., Tb^3+^, Eu^3+^) or a radiometal (e.g., ^64^Cu, ^68^Ga, ^111^In, or ^89^Zr) [23, 24, 25]. In contrast, (radio)metalation via enzymatic polymerization remains unreported, and this knowledge gap could be addressed by drawing inspiration from aforementioned precedents of dNTPs conjuguated to exchange‐inert, group 8 metal complexes.

By using metal chelate‐conjugated dNTPs as substrates in enzymatic polymerization, oligonucleotides could be internally labeled sequence‐specifically for programmable oligonucleotide metalation. In turn, such labeled oligonucleotides would amalgamate the well‐known properties of DNA for information storage and highly specific complementary strand recognition with the bioanalytic and radiological properties of chelated (radio)metals – for potential use both in highly sensitive mass‐spectrometry detection and the development of new radiopharmaceuticals that rely on accuracy and speed (see discussion). Yet, to date, the internal labeling of DNA with multiple (radio)metal chelates remains unknown.

To address this knowledge gap, we report the enzymatic polymerization of two chelate‐modified deoxyribonucleoside triphosphates, yielding DNA strands with multiple chelated metal cations in ratios defined by template strand base‐pairing information. These metalated oligonucleotides retain base‐pairing fidelity. Sequence‐specific (radio)metal incorporation is confirmed by inductively coupled plasma mass spectrometry (ICP‐MS) and autoradiography. Additionally, terminal transferase‐mediated elongation demonstrates non‐templated, enzyme‐mediated 3′‐labeling. This preliminary report establishes a new platform for DNA labeling that enables simultaneous incorporation of two different (radio)metals for bioanalytical and potential radiotheranostic applications.

## 2 Results and Discussion

### 2.1 Programable Metalated dNTP Incorporation Leads to Detection of Metals in Discrete Levels

**FIGURE 2:**
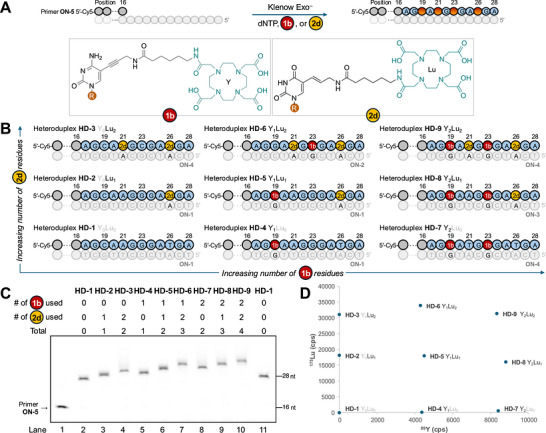
Primer extension in the presence of multiple metalated‐dYTPs and ICP‐MS detection of metalated DNA in discrete levels. (A) An array of metalated DNA oligonucleotides with 0–2 modified cytosines and 0–2 modified thymidines at positions 19, 21, 23, and 26 was synthesized by primer extension with dGTP, dATP, and dCTP or **1b**, and dTTP or **2d**, where grey circles represent commercial oligonucleotides, blue and red‐yellow circles, respectively represent canonical and artificial bases added by the polymerase. (B) An exemplary array of heteroduplexes **HD‐1–9** comprising extended primers hybridized to templates **ON‐1–4**, shown with the expected number of metals added. (C) The strands were separated by denaturing PAGE with 16‐nt primer **ON‐5** as marker and respective triphosphate(s) incorporated as noted. (D) Non‐radioactive ^89^Y and ^175^Lu were detected by ICP‐MS in discrete ratios corresponding to the template sequences (Figure S102, error bars are smaller than the marker).

Non‐radioactive dYTPs (*Y* = pyrimidine: C or T) were synthesized bearing several well‐established linker arms, including the use of strain‐promoted cycloaddition reactions, enjoined to either DOTA or DTPA as chelators (Figure 2C, and additionally in Schemes S2 and S23 for triphosphates **1–4**, Scheme S15 for PEGylated DOTA nucleotide triphosphates **19–24**, as well as Schemes S26 and S31 for the PEGylated DTPA conjugates **35–38**) for late‐stage chelation of (radio)metals. To avoid contamination with unmodified triphosphate precursors and to ensure clean separation by HPLC, a sequential deprotect‐couple‐deprotect strategy was employed. Protected linker/chelator moieties were coupled to the triphosphate, yielding nonpolar products that are readily separated from polar starting materials by reverse‐phase HPLC. Subsequent deprotection affords highly polar products that are similarly well‐resolved from any remaining protected starting material. Although acid‐mediated deprotection of chelator moieties has been reported to yield substantial diphosphate byproducts [26], we observed less than 10% triphosphate degradation (as evidenced by detection of free inorganic phosphate using ^31^P NMR spectroscopy) when reacting dCTP as a test substrate under our deprotection conditions. Following final purification, ^31^P NMR spectroscopy suggested purity of the triphosphate as evidenced by the absence of diphosphate or free phosphate in the product.

These dYTPs are substrates for Klenow Exo^−^ DNA polymerase (Figures S68–S71, and S74–S75). To investigate the role of the linker arm, an expanded series of dYTPs was designed for initial screening. Starting with 5‐aminopropargyl‐dCTP **9** and 5‐aminoallyl‐dUTP **10**, linkers (C_6_ and PEG_n_ where *n* = 4, 12, 24, along with click‐linked dYTPs Scheme S26) were appended to address the effect of steric bulk imposed by the metal complex. Although all constructs were readily polymerized, following optimization, we chose to use dYTPs **1–4** bearing an aminohexanamide (C_6_) linker and characterized these by ^31^P‐NMR spectroscopy. These were first metalated with 1 eq. of naturally (^nat^) abundant, non‐radioactive ^nat^Lu and ^nat^Y, respectively and purified by HPLC (Figures S41–S50). Their (radio)syntheses and time‐dependent enzymatic incorporation are available in the Electronic Supporting information.

A 5′‐Cy5‐primer (**ON‐5**) was hybridized to different oligonucleotide templates and extended in the presence of metalated triphosphates **1b** and **2d**, each at 8 µM (Figure 2). This approach enabled templated incorporation of 0–2 metalated cytosines and 0–2 metalated thymidines at positions defined by the template sequences. Where four modified nucleotides were incorporated, **1b** and **2d** were used at 40 µM (Figure S72, lane 11). Enzymatic synthesis was rapid, with extension complete in 9 min (see Figure S73). Following primer extension, the heteroduplexes **HD‐1–9** was purified by ion‐exchange HPLC (see Figures S91–S101). An aliquot of each was analyzed by denaturing polyacrylamide gel electrophoresis (PAGE) to visualize the fluorescently labeled primer extension products (Figure 2B) thereby confirming full‐length incorporation with no evidence of truncates. An apparent electrophoretic retardation, commensurate with the increased number of modified nucleosides incorporated, provides additional evidence of faithfully templated incorporation.

A direct consequence of sequence‐specific incorporation of **1b** and **2d** is that the Y:Lu ratio is dictated by the template strand sequence. To measure the relative metal concentrations of ^nat^Y and ^nat^Lu, aliquots of the purified heteroduplexes were diluted in environmental‐grade HNO_3_ and analyzed by inductively coupled plasma‐mass spectrometry (ICP‐MS, Figure 2C). Although detection efficiency is known to vary between metals, the discrete metal levels are, within experimental error, proportional to the number of metalated Y‐dCTPs (**1b**), Lu‐dTTPs (**2d**), or both that were incorporated into the full‐length extension product, as dictated by the template sequence. This demonstrates the ability to detect and differentiate metal loading levels in accordance with the high‐fidelity, error‐free template‐directed incorporation of dYTPs.

These results also confirm the exchange inertness of each chelate despite the presence of Mg^2+^ (2–5 mM) in primer extension buffer. To further demonstrate that Mg^2+^ does not displace a metal cation from DOTA, we subjected a clinically evaluated PSMA‐targeting radiotracer labeled with ^68^Ga to incubation in polymerase buffer containing Mg^2+^ (Figure S52). As expected, no exchange was observed. While the stability of FDA‐approved ^177^Lu‐DOTA‐labeled radiotherapeutics, for example, lutetium (^177^Lu) oxodotreotide, in formulation and in the presence of serum Mg^2+^, is ensured by well‐known kinetic stability. Here, this inertness is also guaranteed by thermodynamic stability owing to the 14 orders of magnitude difference in affinity constants: pK_D_[Lu(DOTA)] = 25 versus pK_D_[Mg(DOTA)] = 11 [27]. Under these thermodynamic constraints, no significant exchange can occur at 100 µM Lu(DOTA) in the presence of 10 mM Mg^2+^.

### 2.2 Enzymatic Incorporation of Radiometalated dNTP

**FIGURE 3:**
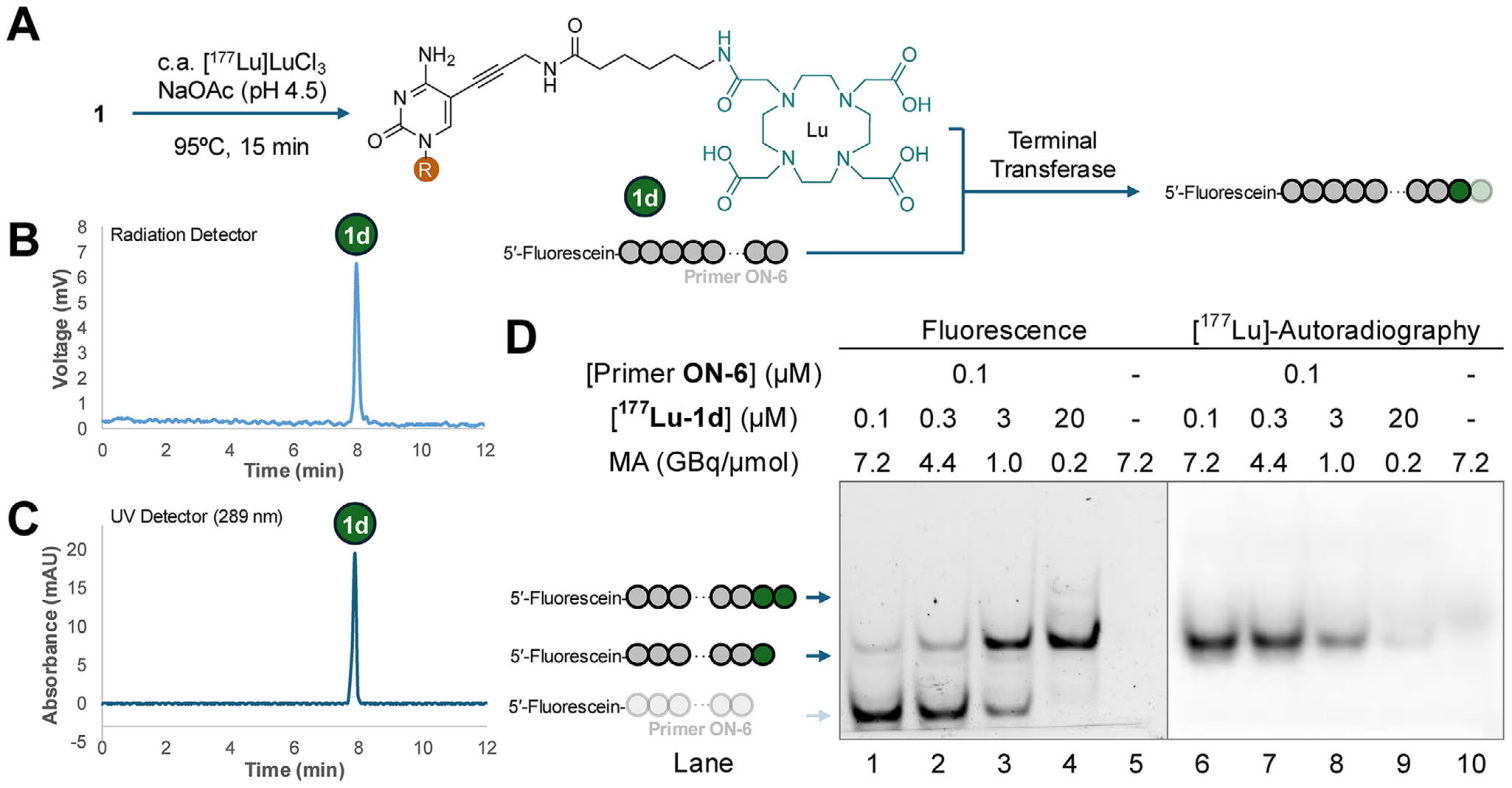
Radiolabeling at 3′‐terminus by incorporation of ^177^ **Lu‐1d** using TdT. (A) Radiolabeling of 1 with c.a. [^177^Lu]LuCl_3_ afforded **1d**, following radio‐HPLC purification: (B) radiation (also in Figure S55) and (C) UV–vis (289 nm, also in Figure S56). Following 3′‐incorporation by TdT with **ON‐6**, the mixture was resolved by PAGE and visualized by (D) fluorescence and autoradiography (also in Figure S85). Grey circles represent primer **ON‐6**, and green circles represent **1d**. The identity of dNTPs and molar activity used are provided below each lane. Other examples such as **^161^Tb‐1c** (Figure S83), **^161^Tb‐2c** (Figure S84), **^177^Lu‐2d** (Figure S86), **^111^In‐3a** (Figure S88), and **^111^In‐4a** (Figure S89) are available in the Supporting Information.

To prepare radiometalated DNA, triphosphate **1** was radiometalated in the presence of ^177^Lu to give **^177^Lu‐1d** (Figure 3A). Owing to safety‐imposed limits on a maximum activity of 300 MBq, which in turn limited the total mass attainable from no carrier added (n.c.a.) ^177^Lu, all radiosyntheses were performed as carrier‐added (c.a.) at a maximum molar activity (A_m_) of 10 GBq µmol^−1^. Lower molar activities were realized by adding increasing amounts of non‐radioactive **1d**. In a preliminary test, terminal deoxynucleotidyl transferase (TdT) was used (Figure 3A) to investigate enzymatic incorporation. Using 5′‐fluorescein‐primer **ON‐6**, TdT introduced a single **^177^Lu‐1d** in a template‐independent fashion. Fluorescent detection of the labeled products along with correlated autoradiography shows enzymatic incorporation of **1d** (Figure 3D). Higher chemical yields were achieved at higher overall concentrations of **1d** (lanes 3–4). Yet at constant radioactivity, increasing the concentration of **1d** by adding non‐radioactive **1d** necessarily lowers the molar activity (A_m_) of **^177^Lu‐1d**, resulting in an apparent reduction in radiochemical yield (lanes 6–9), consistent with isotopic dilution.

The incorporation result is also noteworthy: whereas TdT typically generates a heterogeneous ladder of products [28, 29], even with unnatural nucleobase substrates [30, 31], a single incorporation event is observed here. Serendipitously, this reaction enables the generation of homogeneously labeled products, which is advantageous for applications requiring precise stoichiometric control of radiometal incorporation. As a preliminary explanation for this observation, we propose that the chelator‐modified dCTP may act as a suicide substrate, whereby the incorporated Lu(DOTA) chelate inhibits further polymerization. Consistent with this hypothesis, we show that exogenous Lu(DOTA) at 300 µM effectively reduces the number of deoxycytidines incorporated by TdT (Figure S90). Nevertheless, definitive elucidation of the inhibitory mechanism would require structural studies to identify potential contacts between the metal chelate and the enzyme active site that may stabilize an inhibited conformation.

**FIGURE 4:**
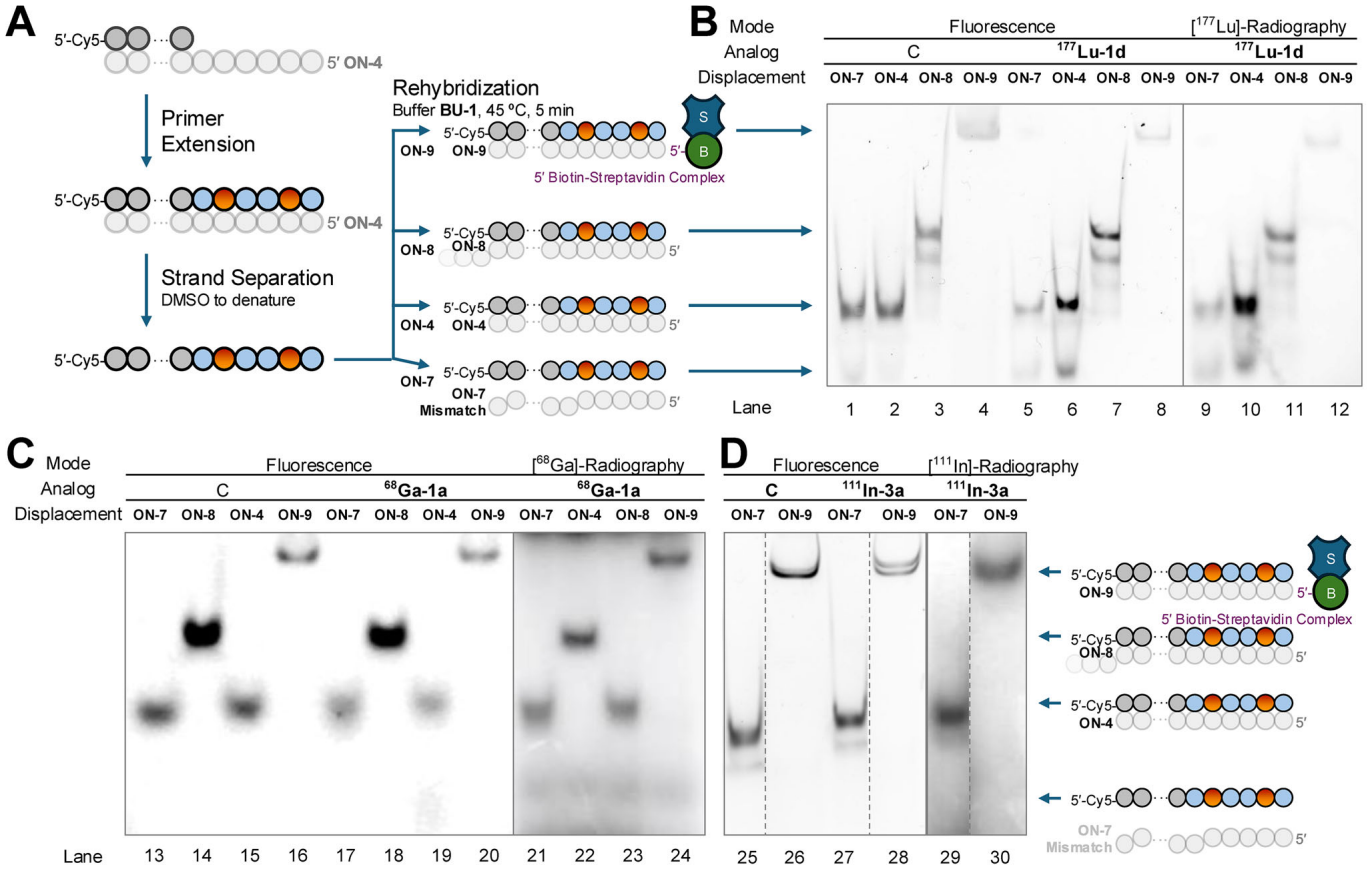
Incorporation of radiometalated dNTPs by primer extension, strand displacement and electrophoretic mobility shift assay (EMSA). (A) Workflow for primer extension, strand dissociation, and strand displacement/association with a competitor: (i) a template‐annealed **ON‐5** is extended by Klenow Exo− polymerase; (ii) following denaturation, oligonucleotides **ON‐7–9** were added to displace the template and hybridize to the extended primer. Grey circles represent commercially obtained oligonucleotides, blue and red circles, respectively represent canonical and artificial bases. The encircled B (green) and blue S polygon represent 5′‐biotin and streptavidin. Sequence‐specific binding validation of (B) **^177^Lu‐1d** (also in Figure S79), (C) **^68^Ga‐1a** (also in Figure S78), and (D) **^111^In‐3a**‐labeled oligonucleotides (also in Figure S81) after strand displacement via EMSA‐based fluorescence and autoradiography imaging. Other examples such as **^177^Lu‐2d** (Figure S80) and **^111^In‐4a** (Figure S82) are available in the Supporting Information.

For proof‐of‐concept in pre‐targeting applications, we sought to demonstrate sequence‐specificity/ strand‐complementarity of radio metalated DNA in the following fashion: **1**. primer extension resulting in a template‐bound heteroduplex (Figure 2B); **2**. strand‐separation to release the radiolabeled primer extension product by adding DMSO as a mild denaturant and applying mild heating to 45° C, and **3**. re‐hybridization challenge using against one non‐complementary mismatched strand (negative control, **ON‐7**) and two complementary strands (**ON‐8** or **ON‐9**) designed to afford different electrophoretic mobilities to demonstrate successful strand hybridization (Figure 4A).

Two primer extension reactions were performed: (**i**) with unmodified dCTP as a control and (**ii**) with **^177^Lu‐1d**, yielding unlabeled and radiolabeled products, respectively. Without further purification, an aliquot of the extended primer/template heteroduplex was added to DMSO either lacking a competing complementary oligonucleotide (negative control), a mismatched **ON‐7** (a second negative control where the primer extension product and its original template **ON‐4** would reassociate), **ON‐8** (a longer complement with a 5′‐T_30_‐tail used in excess as a competitor) or **ON‐9** (a 5′‐biotinylated complement complexed to avidin, representative of an oligonucleotide‐bound protein target). The reaction contents were then heated at 45 °C for 5 min to separate the strands, followed by dilution into strand displacement buffer **B‐1** (Table S3) to dilute the DMSO, and then resolved by native PAGE.

Strand displacement was visualized either by fluorescence for the unmodified control (**i**) and by both fluorescence and autoradiography for internally radiolabeled primer extension products (**ii**). When dCTP, as the non‐radioactive control, was used to calibrate this assay, fluorescence showed no retardation in electrophoretic mobility when strand separation is performed in the absence or presence of the mismatched oligonucleotide **ON‐7** (Figure 4B, lanes 1 and 2, respectively). For the mismatched negative control (lanes 1,5, and 9), following dilution into Buffer **B‐1**, the primer extension product did not hybridize with the mismatched competitor **ON‐7** and instead rehybridized with **ON‐4**, the still‐present original template strand, thus reforming the original heteroduplex **HD‐3** (Figure 2). A minor product is seen as a faster‐running band, seen in lanes 1, 2, 5, 6, 9, and 10 (Figure 4B), which we attribute to be the still‐dissociated single‐stranded primer extension product that did not rehybridize with the template strand. By contrast, when **ON‐8**, a longer complement, is added, a higher‐running band predominates (lane 3); two fainter, lower‐running bands are also observed and their appearance is not readily intuited. Finally, addition of a biotinylated complement **ON‐9** complexed to avidin resulted in a significantly retarded band (lane 4). In nearly identical fashion, primer extension products synthesized from ^**177**^ **Lu‐1d** and treated identically gave the same pattern with fluorescent imaging (lanes 5–8) and recapitulated auto‐radiographically (lanes 9–12). Encouraged by these results, very similar results are obtained with template‐directed primer extension using ^**68**^ **Ga‐1a** (Figure 4C) and ^**111**^ **In‐3a** (Figure 4D).

## 3 Discussion

In the realm of nucleic acids, numerous functionalities have been enzymatically incorporated for example, biotin [32], fluorophores [33, 34, 35, 36], spin‐labels [37], photosensitizers and photocages [22, 38], electrophiles [39, 40, 41], glycosides, and proteins [42], as reviewed [43, 44, 45, 46]. In addition, click‐type reactions have been used post‐polymerization to label DNAs [47, 48]. Yet missing from this repertoire are chelators that can be loaded at will with different (radio)metals to support many applications ranging from bioanalytical detection to the development of multi‐metalated theranostics (*vide infra*). Appreciating the collective interest in expanding the functionality of enzymatically synthesized DNA [45, 49, 50], for the first time, we introduce (radio)metal chelates onto two dYTPs to connect the information‐bearing properties of DNA to metal isotopes for highly sensitive detection methods, most notably, mass spectrometry and radioactive decay. Although we did not label deoxyribopurine triphosphates (dATP, dGTP), it reasons that 7‐deaza‐functionalized analogs could be appended with chelators to double the number of accessible (radio)metals. The use of orthogonal base pairing dNTPs will increase this number to six or eight [6, 51, 52, 53, 54]. An additional consideration is the stability of metal‐chelate modified nucleosides. Previously reported dNTP conjugates bearing exchange‐inert group 8 metal complexes exhibited poor long‐term stability [20]. Although long‐term stability was not evaluated in this study, we recommend fresh preparation of chelate‐modified dNTPs. Such will be obligatory when working with radioisotopes. Another phenomenon related to stability is that of radiolysis, which is known with ^32^P‐labeled dNTPs, [55, 56, 57, 58] as well as with clinically used radiotracers [59]. While radiolysis was not observed at the molar activities used in this report, it may become significant under no‐carrier‐added conditions and can be mitigated with excipients such as ascorbic acid, gentisic acid, or ethanol [60, 61, 62, 63, 64].

In this preliminary work, we pre‐loaded different (radio)metals onto two different nucleosides and incorporated them enzymatically along templates whose sequences dictate the number and ratio of different metals used. Klenow Exo^−^ polymerase provided rapid and efficient primer extension products with no observable truncates. Polymerization was complete in <10 min, making this work compatible with short‐lived isotopes and comparable to click‐type bioconjugation reactions in terms of reaction rates. Although we did not observe truncates, these are known to arise, particularly in certain sequence contexts or at low dNTP concentrations. Nevertheless, polymerases can be evolved with greater capability of polymerizing modified dNTPs to provide for even more efficient incorporation if needed [65, 66, 67].

ICP‐MS analysis of the primer‐extension products, which detects metal cations at parts‐per‐trillion sensitivity, showed metal ratios that matched the template sequence, confirming sequence‐specific, and error‐free incorporation of both modified dYTPs. The lack of signal observed from templates that did not program the incorporation of the modified dYTPs confirm that chelate‐modified dYTPs are not misincorporated with any substantial frequency. These results demonstrate that chelate‐modified nucleosides enable customizable incorporation of (radio)metals with control over both elemental/isotopic identity and relative abundance.

We posit that the above ICP‐MS work holds great promise for bioanalytical applications where laser ablation (LA) ICP‐MS should enable detection of metalated probes directly on a solid tissue sample [68], akin to fluorescent in situ hybridization (FISH) [69]. Such would be analogous to metalated immunocytochemistry reagents for imaging mass cytometry that exploit LA‐ICP‐MS for sensitive detection, including loading with different stable isotope ratios [70]. In addition, for spatio‐temporal probing with ICP‐MS, isotopic encoding of oligonucleotides is now possible: dCTP could be loaded with one or more “light” isotopes of a metal e.g. ^155^Gd while dTTP could be loaded with one or more “heavy” isotopes of a metal, e.g. ^158^Gd. Synthesis of metalated oligonucleotides should also find use with isothermal amplification methods for cancer and pathogen detection including rolling circle amplifications (RCA) and loop‐mediated (LAMP) approaches [71, 72, 73, 74]. Metalated nucleotides could also be used in aptamer selection through SELEX, where the inclusion of the built‐in radiometal complexes would accelerate radiotheranostic development.

For applications in nuclear medicine, radiometalated oligonucleotides can be used for both diagnosis and therapy. Toward this eventuality, we demonstrate that such constructs can be rapidly synthesized, easily dissociated from templates (45 °C in DMSO), and re‐hybridized with complementary strands. It thus reasons that such constructs should find use in pre‐targeting applications where the intended target complement would be conjugated to a therapeutic antibody.

In terms of radiotracer development, no‐carrier‐added (NCA) syntheses are standard practice for achieving high effective molar activity (A_m_). Owing to practical constraints related to safety limits, we performed carrier‐added syntheses to ensure good yields in primer extension reactions (Figure S77). For primer extension, ∼10.5 MBq of **^177^Lu‐1d** was supplemented with 1.2 nmol **^nat^Lu‐1d**, yielding a final A_m_ = 8.8 GBq µmol^−1^. For comparison, FDA‐approved ^177^Lu‐vipivotide tetraxetan (Pluvicto) is clinically dispensed at 50 GBq µmol^−1^; only 6‐fold higher than what we achieved under these carrier‐added conditions. Notwithstanding the use of carrier, the potential for radiolabeling dYTPs under n.c.a. conditions is readily anticipated based on the historic production of n.c.a. [α‐^32^P]‐dATP at >1 GBq and n.c.a. ^177^Lu at >500 GBq, along with the development of microfluidic reactors [75, 76]. Notably, the incorporation of multiple radiometals further increases the effective A_m_ of the products of primer extension [77].

An emerging challenge in radiopharmaceuticals is the creation of so‐called radiohybrids that combine diagnostic and therapeutic isotopes within the same molecule as seen in radiofluorinated/radio metalated tracers [78, 79, 80]. Yet this approach becomes chemically challenging when labeling with two metal isotopes. Solutions are limited to the use of theranostic (radio)metal pairs that can be orthogonally chelated by two different chelators appended to a single bioconjugate (for example, Cu(NOTA)/Lu(DOTA)) [81, 82], or by sequential conjugation of a metal‐chelate to a pre‐metalated bioconjugate.

Here, orthogonality is achieved by the polymerase that enforces specific base‐pairing interactions during primer extension [83] to ensure appropriate incorporation of multiple different (radio)metals irrespective of the chelator used. Although practical safety considerations limited us to single‐radiometal labeling, we successfully demonstrated the incorporation of two different metals – each chelated by DOTA – on a single DNA strand using ICP‐MS, highlighting applicability to CyTOF.

Typically, enzymes have not been used in nuclear medicine to radiolabel complex molecules, a notable exception being fluorinase that catalyzes the nucleophilic addition of [^18^F]fluoride onto *S*‐adenosylmethionine‐conjugated peptidic precursors [84, 85]. By contrast, enzymes dominate nucleic acid applications ranging from analytical applications to synthesis on‐scale. Nevertheless, enzymes are increasingly used in the synthesis of biopharmaceuticals [86] with noted utility in enantioselective synthesis of short‐lived ^11^C‐labeled radiotracers [87]. Here, we extend the use of enzymes to label nucleic acids with radioisotopes typically used in radiopharmaceuticals.

The use of enzymes in radiosynthesis need not be limited to template‐directed DNA synthesis. The use of terminal transferase, a template‐independent enzyme, points to a broader approach for developing other transferases that will site‐specifically transfer chelated radiometal substrates onto diverse biomolecules such as proteins, antibodies, affibodies, and glycosides etc. Logical extensions of this approach include enzymatic ADP‐ribosylation, *trans*‐glutamination, farnesylation, ligation, *trans*‐amidation and glycosylation [88]. It is also appreciated that ribozymes capable of using modified dNTPs with relaxed tolerance for modification could also be used for labeling methods proposed herein [92]. Given a growing interest in expanding the chemical diversity of DNA and interests in new radiosynthetic methods, this work offers a foundation for further exploration at their intersection.

## 4 Conclusion

This work discloses the production of (radio)metal complexes into DNA oligonucleotides by enzymatic polymerization of chelate‐modified nucleotide triphosphates. Use of polymerases to incorporate metal‐chelate‐modified nucleosides into DNA results in oligonucleotides that combine the informational precision of DNA with the unique analytical, imaging, and therapeutic properties of (radio)metals. The integration of metal chelates on DNA nucleobases, thus defines a programmable, multifunctional, and biologically compatible class of materials thus bridging (radio)chemistry and nucleic acid biology. This should provide flexibility in probe generation for bioanalytical methods (e.g., ICP‐MS) and radiopharmaceutical design. By integrating multiple (radio)metals including ^nat/68^Ga, ^nat^Y, ^nat/111^In, ^nat/161 ^Tb, and ^nat/177^Lu, along with different linkers and chelators (e.g., DOTA, DTPA) within a base‐pairing architecture, the programmable multi‐metal platform establishes a new paradigm for metalated molecular probe development for theranostic development and mass spectrometry probes. This strategy provides precise control over chelator placement within oligonucleotide sequences while enabling multiple metal incorporations: A feature unprecedented in (radio)metalated oligonucleotides to date. Such versatility facilitates the development of radiohybrids adaptable to various isotopes for theranostic applications.

## Author Contributions

**Antonio A. W. L. Wong**: conceptualization, investigation, methodology, validation, writing – review and editing, formal analysis, data curation, writing – original draft, and visualization. **François Bénard**: funding acquisition, writing – review and editing, project administration, supervision, resources, formal analysis, and visualization. **David M. Perrin**: conceptualization, funding acquisition, writing – original draft, methodology, visualization, writing – review and editing, formal analysis, project administration, resources, and supervision.

## Conflicts of Interest

A provisional patent application has been filed on compositions by the described authors.

## Supporting information




**Supporting File**: The authors have cited additional references within the Supporting Information [23, 89, 90, 91, 92].

## Data Availability

The data that support the findings of this study are available from the corresponding author upon reasonable request.
