# Supplementary material for: Toward Multi(radio)metalated DNA: Enzymatic Polymerization of Metal‐Chelate‐Modified Deoxyribonucleoside Triphosphates
Source: Angew Chem Int Ed Engl. 2026 May 7;65(27):e1429706. doi: 10.1002/anie.1429706 (PMC13327564; doi:10.1002/anie.1429706)
Supplement: Supplementary file 1 — Supporting File: The authors have cited additional references within the Supporting Information [23, 89, 90, 91, 92]. [file ANIE-65-e1429706-s001.pdf]

Supplementary Materials:
Towards Multi(radio)metalated DNA: Enzymatic Polymerization of Metal-Chelate-Modified Deoxyribonucleoside Triphosphates

Antonio A. W. L. Wong,[a],[b] François Bénard,[a],[c],[d] David M. Perrin[b]*

Affiliations:
[a] Department of Basic and Translational Research, BC Cancer Research Institute; Vancouver, BC V5Z 1L3, Canada
[b] Department of Chemistry, University of British Columbia; Vancouver, BC V6T 1Z1, Canada
[c] Department of Molecular Imaging and Therapy, BC Cancer; Vancouver, BC V5Z 4E6, Canada
[d] Department of Radiology, University of British Columbia; Vancouver, BC V5Z 1M9, Canada
* Corresponding author David M. Perrin dperrin@chem.ubc.ca.

Table of Contents

List of Supplementary Figures 3

List of Supplementary Schemes 8

List of Supplementary Tables 9

Preamble 10

Sourcing of Commercial Available Starting Materials 10

Equipment and Consumables 13

Buffer Formulations 14

Methods for High Performance Liquid Chromatography (HPLC) 15

Gel Image Presentation 17

Chemical Synthesis 17

A. Synthesis of XadYTP-AHX-DOTA Series 17

Fmoc-AHX-OSu **5** ... 17
General acylation step with Fmoc-AHX-OSu **5** ... 19
General Fmoc deprotection ... 20
General acylation step with DOTA-mono-NHS tris (*t*-Bu ester) **13** ... 21
General deprotection with TFA ... 23

B. Synthesis of XadYTP-PEG$_n$-DOTA series ... 25
General acylation steps with DOTA-PEG$_n$-TFP **16–18** ... 25

C. Synthesis of XadYTP-Linker-DTPA series ... 32
DTPA-Tetrazine **25** and byproduct DTPA-Bis-tetrazine **26** ... 32
General acylation steps with DTPA-Bis-anhydride **27** ... 34
General acylation steps with TCO-PEG$_4$-OSu **29** and TCO-PEG$_{12}$-OSu **30** ... 37
General SPAAC steps with DTPA-Tetrazine **25** ... 40

D. Non-radioactive synthesis of XadYTP-AHX-DOTA(M) 1a–d, 2a–d and XadYTP-AHX-DTPA(In) 3a, 4a .. 43
General non-radioactive labeling ... 43

NMR Chelation Test ... 49

HPLC Stability Test ... 50

Radiosynthesis ... 51

E. Radiosynthesis of XadYTP-AHX-DOTA(M) and XadYTP-AHX-DTPA(In) series ... 51
General radioactive labeling ... 51
[^{68}Ga]-PadCTP-AHX-DOTA(Ga) **^{68}Ga-1a** ... 52
[^{161}Tb]-PadCTP-AHX-DOTA(Tb) **^{161}Tb-1c** ... 52
[^{177}Lu]-PadCTP-AHX-DOTA(Lu) **^{177}Lu-1d** ... 53
[^{161}Tb]-AadUTP-AHX-DOTA(Tb) **^{161}Tb-2c** ... 54
[^{177}Lu]-AadUTP-AHX-DOTA(Lu) **^{177}Lu-2d** ... 54
[^{111}In]-PadCTP-AHX-DTPA(In) **^{111}In-3a** ... 55
[^{111}In]-AadUTP-AHX-DTPA(In) **^{111}In-4a** ... 56

DNA Sequences and Gel Electrophoresis ... 57

Oligonucleotide Sequences ... 57

Polyacrylamide Gel Electrophoresis (PAGE) ... 57
Purification of Oligonucleotides by Denaturing PAGE ... 57
Analysis of Denatured DNA Strands ... 57
Analysis of Native DNA Strands ... 58
Analysis of Denatured Protein Samples (SDS-PAGE) ... 58

Cloning of Klenow Exo$^-$ ... 58

Plasmid for Klenow Exo$^-$ Expression ... 58

Cloning, Expression, Purification, and Characterization of Klenow Exo$^-$ Polymerase ... 61

Klenow Exo$^-$ Polymerase Activity Assays ... 62

Routine Primer Extension 62

Standardized Primer Extensions 62

Primer Extension with up to 160 µM Modified Nucleotide Triphosphates 68

Primer Extension with XadYTP-PEG$_n$-DOTA series 19–24 70

Deleterious Effects of Superstoichiometric Metal Trication on Primer Extension 72

Radioactive Primer Extension 73

Electrophoretic Mobility Shift Assay (EMSA) 74

Terminal Transferase 3′-Extension 76

Terminal Deoxynucleotidyl Transferase (Radio)labeling 76

Determination of Lu(DOTA) as an Inhibitor of Terminal Deoxynucleotidyl Transferase 79

Inductively Coupled Plasma–Mass Spectrometry (ICP-MS) 80

Preparation of ICP-compliant Reagents, Calibration Standards and Analytes 80

Anionic Exchange HPLC of Oligonucleotides for ICP-MS Analysis Method M-11 81

Oligonucleotides HPLC Traces 81

Sample Introduction and ICP-MS Settings 83

ICP-MS Source Data 84

List of Supplementary Figures

Figure S1. ^{1}H NMR spectrum of Fmoc-AHX-OSu **5**. 18
Figure S2. ^{13}C NMR spectrum of Fmoc-AHX-OSu **5**. 18
Figure S3. UV-Vis spectrum of Fmoc-AHX-OSu **5**. 19
Figure S4. HPLC chromatogram of PadCTP-AHX-DOTA **1**. 23
Figure S5. ^{31}P NMR spectrum of PadCTP-AHX-DOTA **1**. 24
Figure S6. UV-Vis spectrum of PadCTP-AHX-DOTA **1**. 24
Figure S7. HPLC chromatogram of AadUTP-AHX-DOTA **2**. 25
Figure S8. ^{31}P NMR spectrum of AadUTP-AHX-DOTA **2**. 25
Figure S9. UV-Vis spectrum of AadUTP-AHX-DOTA **2**. 25
Figure S10. HPLC chromatogram of PadCTP-PEG$_4$-DOTA **19**. 26
Figure S11. UV-Vis spectrum of PadCTP-PEG$_4$-DOTA **19**. 26
Figure S12. HPLC chromatogram of PadCTP-PEG$_{12}$-DOTA **20**. 27
Figure S13. UV-Vis spectrum of PadCTP-PEG$_{12}$-DOTA **20**. 27
Figure S14. HPLC chromatogram of PadCTP-PEG$_{24}$-DOTA **21**. 28
Figure S15. UV-Vis spectrum of PadCTP-PEG$_{24}$-DOTA **21**. 28

Figure S16. HPLC chromatogram of AadUTP-PEG$_4$-DOTA **22**. 29
Figure S17. UV-Vis spectrum of AadUTP-PEG$_4$-DOTA **22**. 29
Figure S18. HPLC chromatogram of AadUTP-PEG$_{12}$-DOTA **23**. 30
Figure S19. UV-Vis spectrum of AadUTP-PEG$_{12}$-DOTA **23**. 30
Figure S20. HPLC chromatogram of AadUTP-PEG$_{24}$-DOTA **24**. The minor peak t_R = 5.7 min was 2,3,5,6-tetrafluorophenol, which was inert in further reactions. 31
Figure S21. UV-Vis spectrum of AadUTP-PEG$_{24}$-DOTA **24**. 31
Figure S22. ^{1}H NMR spectrum of DTPA-Tetrazine **25**. 33
Figure S23. ^{13}C NMR spectrum of DTPA-Tetrazine **25**. 33
Figure S24. ^{13}C NMR spectrum of DTPA-Tetrazine **25**, zoomed in f_1 50.0–65.0 ppm region. 34
Figure S25. UV-Vis spectrum of DTPA-Tetrazine **25**. 34
Figure S26. HPLC chromatogram of PadCTP-AHX-DTPA **3**. 35
Figure S27. ^{31}P NMR spectrum of PadCTP-AHX-DTPA **3**. 35
Figure S28. UV-Vis spectrum of PadCTP-AHX-DTPA **3**. 35
Figure S29. HPLC chromatogram of AadUTP-AHX-DTPA **4**. 36
Figure S30. ^{31}P NMR spectrum of AadUTP-AHX-DTPA **4**. 36
Figure S31. UV-Vis spectrum of AadUTP-AHX-DTPA **4**. 36
Figure S32. UV-Vis spectrum of PadCTP-PEG$_4$-TCO **31**........ 38
Figure S33. UV-Vis spectrum of PadCTP-PEG$_{12}$-TCO **32**. 38
Figure S34. UV-Vis spectrum of AadUTP-PEG$_4$-TCO **33**........ 39
Figure S35. UV-Vis spectrum of AadUTP-PEG$_{12}$-TCO **34**. 39
Figure S36. UV-Vis spectrum of PadCTP-PEG$_4$-DTPA **35**........ 41
Figure S37. UV-Vis spectrum of PadCTP-PEG$_{12}$-DTPA **36**. 41
Figure S38. UV-Vis spectrum of AadUTP-PEG$_4$-DTPA **37**........ 42
Figure S39. UV-Vis spectrum of AadUTP-PEG$_{12}$-DTPA **38**. 43
Figure S40. Structures of precursors **1–4**........ 44
Figure S41. UV-Vis spectrum of PadCTP-AHX-DOTA(Ga) **1a**........ 44
Figure S42. UV-Vis spectrum of PadCTP-AHX-DOTA(Y) **1b**........ 45
Figure S43. UV-Vis spectrum of PadCTP-AHX-DOTA(Y) **1c**. 45
Figure S44. UV-Vis spectrum of PadCTP-AHX-DOTA(Lu) **1d**. 46
Figure S45. UV-Vis spectrum of AadUYP-AHX-DOTA(Ga) **2a**. 46
Figure S46. UV-Vis spectrum of AadUTP-AHX-DOTA(Y) **2b**........ 47
Figure S47. UV-Vis spectrum of AadUTP-AHX-DOTA(Tb) **2c**. 47
Figure S48. UV-Vis spectrum of AadUTP-AHX-DOTA(Lu) **2d**. 48
Figure S49. UV-Vis spectrum of PadCTP-AHX-DTPA(In) **3a**........ 48
Figure S50. UV-Vis spectrum of AadUTP-AHX-DTPA(In) **4a**........ 49
Figure S51. Coordination competition between the triphosphate and DOTA moieties. [1] PPT refers to tendencies of white precipitate formation prior to 95ºC heating. The precipitates did not solubilize during heating. 49
Figure S52. [^{68}Ga]Ga-HTK03149 stability study via radio-HPLC in selected conditions. (**A**) NEBuffer 2, 1X, 60 min, incubated in RT; (**B**), NaOH (10 mM), 1 min, incubated in RT; (**C**) TBE, 0.5X, 45 min, incubated in 65ºC; (**D**) H_2O as negative control, 60 min, incubated in RT. 51
Figure S53. Radioactive HPLC trace of [^{161}Tb]-PadCTP-AHX-DOTA(Tb) **^{161}Tb-1c**. 52
Figure S54. UV (λ = 289 nm) HPLC trace of [^{161}Tb]-PadCTP-AHX-DOTA(Tb) **^{161}Tb-1c**. 53

Figure S55. Radioactive HPLC trace of [^{177}Lu]-PadCTP-AHX-DOTA(Lu) **^{177}Lu-1d**. 53
Figure S56. UV (λ = 289 nm) HPLC trace of [^{177}Lu]-PadCTP-AHX-DOTA(Lu) **^{177}Lu-1d**. 53
Figure S57. Radioactive HPLC trace of [^{161}Tb]-AadUTP-AHX-DOTA(Tb) **^{161}Tb-2c**. 54
Figure S58. UV (λ = 289 nm) HPLC trace of [^{161}Tb]-AadUTP-AHX-DOTA(Tb) **^{161}Tb-2c**. 54
Figure S59. Radioactive HPLC trace of [^{177}Lu]-AadUTP-AHX-DOTA(Lu) **^{177}Lu-2d**. 55
Figure S60. UV (λ = 289 nm) HPLC trace of [^{177}Lu]-AadUTP-AHX-DOTA(Lu) **^{177}Lu-2d**. 55
Figure S61. Radioactive HPLC trace of [^{111}In]-PadCTP-AHX-DTPA(In) **^{111}In-3a**. 55
Figure S62. UV (λ = 289 nm) HPLC trace of [^{111}In]-PadCTP-AHX-DTPA(In) **^{111}In-3a**. 56
Figure S63. Radioactive HPLC trace of [^{111}In]-AadUTP-AHX-DTPA(In) **^{111}In-4a**. 56
Figure S64. UV (λ = 289 nm) HPLC trace of [^{111}Tb]-PadCTP-AHX-DOTA(Tb) **^{111}In-1c**. 56
Figure S65. Plasmid Map of **pET-28(+)-KlenowExo⁻**. The plasmid contains a kanamycin resistance (*KanR*) gene for antimicrobial selection, and is inducible via the *lac* operon. The gene of interest containing Klenow Exo⁻ is flanked by polyhistidine sequences for downstream purification by immobilized metal affinity chromatography (IMAC). 59
Figure S66. Purification of Klenow Exo⁻ polymerase following IMAC. Aliquots of Klenow Exo⁻ polymerase at different stages of purification were separated by SDS-PAGE following Coomassie Brilliant Blue staining. Lane 1, Molecular ladder (ML); Lane 2, Lysate (L); Lane 3, Sample flow through (FT); Lane 4, Wash 1 (W1) with 10 mM imidazole in **B-15**; Lane 5, Wash 2 (W2); Lane 6, Wash 3 (W3) with 50 mM imidazole in **B-15**; Lane 7, Wash 4 (W4); Lane 8, Wash 5 (W5); Lane 9, Elution (E) with 500 mM imidazole in **B-15**; Lane 10, Klenow Exo⁻ Fragment (KF) standard (73 kDa). 62
Figure S67. Preliminary functional validation of in-house Klenow Exo⁻ polymerase against commercial (New England Biolabs NEB) sample with only canonical nucleotide triphosphates. 63
Figure S68. Primer extension using template **ON-1**, 5′-Cy5-labeled primer **ON-5** (16 nt), and modified nucleotides **1a** and **2d**. (**A**) The expected heteroduplex **HD-5** in the case where both **1a** and **2d** were used in lieu of dCTP (C) or dTTP (T) respectively. Grey circles represent commercially obtained oligonucleotides, blue, red, and yellow circles respectively represent canonical dNTPs, **1a**, and **2d** added by the polymerase. (**B**) PEx products were resolved by denaturing PAGE with different degrees of modification. Final dNTP concentrations for each primer extension are indicated in the respective lanes. 64
Figure S69. Primer extension using template **ON-2**, 5′-Cy5-labeled primer **ON-5** (16 nt), and modified nucleotides **1a** and **2d**. (**A**) The expected heteroduplex **HD-6** in the case where both **1a** and **2d** were used in lieu of dCTP (C) or dTTP (T) respectively. Grey circles represent commercially obtained oligonucleotides, blue, red, and yellow circles respectively represent canonical dNTPs, **1a**, and **2d** added by the polymerase. (**B**) PEx products were resolved by denaturing PAGE with different degrees of modification. Final dNTP concentrations for each primer extension are indicated in the respective lanes. 65
Figure S70. Primer extension using template **ON-3**, 5′-Cy5-labeled primer **ON-5** (16 nt), and modified nucleotides **1a** and **2d**. (**A**) The expected heteroduplex **HD-8** in the case where both **1a** and **2d** were used in lieu of dCTP (C) or dTTP (T) respectively. Grey circles represent commercially obtained oligonucleotides, blue, red, and yellow circles respectively represent canonical dNTPs, **1a**, and **2d** added by the polymerase. (**B**) PEx products were resolved by denaturing PAGE with different degrees of modification. Final dNTP concentrations for each primer extension are indicated in the respective lanes. 66

Figure S71. Primer extension using template **ON-4**, 5′-Cy5-labeled primer **ON-5** (16 nt), and modified nucleotides **1a** and **2d**. (**A**) The expected heteroduplex **HD-9** in the case where both **1a** and **2d** were used in lieu of dCTP (C) or dTTP (T) respectively. Grey circles represent commercially obtained oligonucleotides, blue, red, and yellow circles respectively represent canonical dNTPs, **1a**, and **2d** added by the polymerase. (**B**) PEx products resolved by denaturing PAGE with different degrees of modification. Final dNTP concentrations for each primer extension are indicated in the respective lanes. 67
Figure S72. Primer extension using templates **ON-1–4**, 5′-Cy5-labeled primer **ON-5** (16 nt), and modified nucleotides **1b** and **2d**. PEx products were resolved by denaturing PAGE with different degrees of modification. Final dNTP concentrations for each primer extension are indicated in the respective lanes. 68
Figure S73. Primer extension using templates **ON-1** or **ON-4**, 5′-Cy5-labeled primer **ON-5** (16 nt), and modified nucleotides **1b** and **2d**. Aliquots of PEx products were removed at time points 9, 30 or 60 min and resolved by denaturing PAGE with different degrees of modification. Final dNTP concentrations for each primer extension are indicated in the respective lanes..69
Figure S74. Primer extension using template **ON-10**, 5′-Cy5-labeled primer **ON-5** (16 nt), commercially available nucleotide triphosphates dCTP (C), 5-propargylamino-dCTP **9**, and modified counterparts **19–21**. (**A**) The expected heteroduplex product after primer extension. Grey circles represent commercially obtained oligonucleotides, blue and red-yellow circles respectively represent canonical dNTPs and the nucleobase to be added to position 17. (**B**) The PEx products were resolved by denaturing PAGE..70
Figure S75. Primer extension using template **ON-11**, 5′-Cy5-labeled primer **ON-5** (16 nt), commercially available nucleotide triphosphates dCTP (C), 5-aminoallyl-dUTP **10**, and modified counterparts **22–24**. (**A**) The expected heteroduplex product after primer extension. Grey circles represent commercially obtained oligonucleotides, blue and red-yellow circles respectively represent canonical dNTPs and the nucleobase to be added to position 17. (**B**) The PEx products were resolved by denaturing PAGE..71
Figure S76. Primer extension using template **ON-1**, 5′-Cy5-labeled primer **ON-5** (16 nt), modified nucleotide triphosphates **1**, **2**, **1b**, **2d**. (**A**) The expected heteroduplex **HD-5** in the case where both **1b** and **2d** were used in lieu of dCTP (C) or dTTP (T) respectively. Grey circles represent commercially obtained oligonucleotides, blue, red, and yellow circles respectively represent canonical dNTPs, **1b**, and **2d** added by the polymerase. (**B**) The PEx products were resolved by denaturing PAGE. Final MCl_3 and dNTP concentrations for each primer extension are indicated in the respective lanes. 72
Figure S77. Typical radiolabeling of nucleotide triphosphates following primer extension with relevant radiochemical calculations, using **[^{177}Lu]-1d** as an example. (**A**) Radiosynthesis of **[^{177}Lu]-1d** with carrier added [^{177}Lu]$LuCl_3$, following HPLC purification. (**B**) Formulation of **[^{177}Lu]-1d** for primer extension by resuspending with dried **1d** (1.2 nmol) and canonical dNTPs with purified **[^{177}Lu]-1d** (15 μL at 700 kBq μL^{-1}). A 2X Master Mixture containing the annealed primer-template duplex, Klenow Exo$^-$ polymerase, yeast inorganic pyrophosphatase (YIPP), and buffer salts was prepared ahead of time. (**C**) Primer Extension commenced by mixing 2X Master Mix and the formulated (radio)nucleotide triphosphate at 1:1 volume ratio, at a final volume of 30 μL. 73

Figure S78. Primer extension using template **ON-4**, 5′-Cy5-labeled primer **ON-5** (16 nt), and modified nucleotide **^{68}Ga-1a**. (**A**) The expected heteroduplex in the case where **1a** was used in lieu of dCTP (C). Grey circles represent commercially obtained oligonucleotides, and red circles respectively represent **^{68}Ga-1a** added by the polymerase. (**B**) PEx products were strand-separated and rehybridized with **ON-4** or **ON-7–9**. The newly formed heteroduplexes were resolved by native PAGE. 74
Figure S79. Primer extension using template **ON-4**, 5′-Cy5-labeled primer **ON-5** (16 nt), and modified nucleotide **^{177}Lu-1d**. (**A**) The expected heteroduplex in the case where **1d** was used in lieu of dCTP (C). Grey circles represent commercially obtained oligonucleotides, and red circles respectively represent **^{177}Lu-1d** added by the polymerase. (**B**) PEx products were strand-separated and rehybridized with **ON-4** or **ON-7–9**. The newly formed heteroduplexes were resolved by native PAGE. 74
Figure S80. Primer extension using template **ON-4**, 5′-Cy5-labeled primer **ON-5** (16 nt), and modified nucleotide **^{177}Lu-2d**. (**A**) The expected heteroduplex in the case where **2d** was used in lieu of dTTP (T). Grey circles represent commercially obtained oligonucleotides, and red circles respectively represent **^{177}Lu-2d** added by the polymerase. (**B**) PEx products were strand-separated and rehybridized with **ON-4** or **ON-7–9**. The newly formed heteroduplexes were resolved by native PAGE. 75
Figure S81. Primer extension using template **ON-4**, 5′-Cy5-labeled primer **ON-5** (16 nt), and modified nucleotide **^{111}In-3a**. (**A**) The expected heteroduplex in the case where **3a** was used in lieu of dCTP (C). Grey circles represent commercially obtained oligonucleotides, and red circles respectively represent **^{111}In-3a** added by the polymerase. (**B**) PEx products were strand-separated and rehybridized with **ON-4** or **ON-7–9**. The newly formed heteroduplexes were resolved by native PAGE. 75
Figure S82. Primer extension using template **ON-4**, 5′-Cy5-labeled primer **ON-5** (16 nt), and modified nucleotide **^{111}In-4a**. (**A**) The expected heteroduplex in the case where **4a** was used in lieu of dTTP (T). Grey circles represent commercially obtained oligonucleotides, and red circles respectively represent **^{111}In-4a** added by the polymerase. (**B**) PEx products were strand-separated and rehybridized with **ON-4** or **ON-7–9**. The newly formed heteroduplexes were resolved by native PAGE. 76
Figure S83. 3′-terminal transferase reaction using 5′-fluorescein-labeled primer **ON-6** (22 nt), and one of dCTP (C), and modified nucleotides **1c**, and **^{161}Tb-1c**. Concentrations and molar activities (for radioactive dNTPs) are indicated in each lane. 76
Figure S84. 3′-terminal transferase reaction using 5′-fluorescein-labeled primer **ON-6** (22 nt), and one of dTTP (T), and modified nucleotides **2c**, and **^{161}Tb-2c**. Concentrations and molar activities (for radioactive dNTPs) are indicated in each lane. 77
Figure S85. 3′-terminal transferase reaction using 5′-fluorescein-labeled primer **ON-6** (22 nt), and one of dCTP (C), and modified nucleotides **1d**, and **^{177}Lu-1d**. Concentrations and molar activities (for radioactive dNTPs) are indicated in each lane. 77
Figure S86. 3′-terminal transferase reaction using 5′-fluorescein-labeled primer **ON-6** (22 nt), and one of dTTP (T), and modified nucleotides **2d**, and **^{177}Lu-2d**. Concentrations and molar activities (for radioactive dNTPs) are indicated in each lane. 78
Figure S87. Cropping of original fluorescence and autoradiographic images to produce dataset for 3′-terminal transfer of dTTP (T), modified nucleotides **2d**, or **^{177}Lu-2d** to primer **ON-6** (**Figure S86**) from original images. Dataset (**A**) was collected by cropping the marked

sections (**B**) of the unmarked images (**C**). Lane numbers in **Figure S86** are recapitulated for clarity. 78
Figure S88. 3′-terminal transferase reaction using 5′-fluorescein-labeled primer **ON-6** (22 nt), and one of dCTP (C), and modified nucleotides **3a**, and **^{111}In-3a**. Concentrations and molar activities (for radioactive dNTPs) are indicated in each lane. 79
Figure S89. 3′-terminal transferase reaction using 5′-fluorescein-labeled primer **ON-6** (22 nt), and one of dTTP (T), and modified nucleotides **4a**, and **^{111}In-4a**. Concentrations and molar activities (for radioactive dNTPs) are indicated in each lane. 79
Figure S90. 3′-terminal transferase reaction using 5′-Cy5-labeled primer **ON-5** (16 nt), dCTP, and inhibitor Lu(DOTA). Concentrations are indicated in each lane. 80
Figure S91. HPLC Trace of primer **ON-5**. 81
Figure S92. HPLC Trace of primer **ON-5** hybridized to template **ON-1**. 81
Figure S93. HPLC Trace of heteroduplex **HD-1**. 81
Figure S94. HPLC Trace of heteroduplex **HD-2**. 82
Figure S95. HPLC Trace of heteroduplex **HD-3**. 82
Figure S96. HPLC Trace of heteroduplex **HD-4**. 82
Figure S97. HPLC Trace of heteroduplex **HD-5**. 82
Figure S98. HPLC Trace of heteroduplex **HD-6**. 82
Figure S99. HPLC Trace of heteroduplex **HD-7**. 83
Figure S100. HPLC Trace of heteroduplex **HD-8**. 83
Figure S101. HPLC Trace of heteroduplex **HD-9**. 83
Figure S102. ICP-MS data with error bars, before background subtraction. Error bars are smaller than the marker size. 84

List of Supplementary Schemes

Scheme S1. Synthesis of Fmoc-AHX-OSu **5**. 17
Scheme S2. General synthesis strategy for **1** and **2**. PPPO refers to 5′-*O*-triphosphate. 19
Scheme S3. General acylation reaction with Fmoc-AHX-OSu **5**. 19
Scheme S4. Synthesis of PadCTP-AHX-Fmoc **7**. 20
Scheme S5. Synthesis of AadUTP-AHX-Fmoc **8**. 20
Scheme S6. General Fmoc-removal in 20% (v/v) piperidine/ DMF. 20
Scheme S7. Synthesis of PadCTP-AHX-NH_2 **11**. 21
Scheme S8. Synthesis of AadUTP-AHX-NH_2 **12**. 21
Scheme S9. General acylation reaction with DOTA(tBu)$_3$-OSu **13**. 21
Scheme S10. Synthesis of PadCTP-AHX-DOTA(tBu)$_3$ **14**. 22
Scheme S11. Synthesis of AadUTP-AHX-DOTA(tBu)$_3$ **15**. 22
Scheme S12. General *tert*-butyl ester removal by 95:2.5:2.5 TFA/TIS/H_2O. 23
Scheme S13. Synthesis of PadCTP-AHX-DOTA **1**. 23
Scheme S14. Synthesis of AadUTP-AHX-DOTA **2**. 24
Scheme S15. General synthetic strategy for PEGylated series **19–24**. 25
Scheme S16. Synthesis of PadCTP-PEG$_4$-DOTA **19**. 26
Scheme S17. Synthesis of PadCTP-PEG$_{12}$-DOTA **20**. 27
Scheme S18. Synthesis of PadCTP-PEG$_{24}$-DOTA **21**. 28
Scheme S19. Synthesis of AadUTP-PEG$_4$-DOTA **22**. 29

Scheme S20. Synthesis of AadUTP-PEG$_{12}$-DOTA **23.** 30
Scheme S21. Synthesis of AadUTP-PEG$_{24}$-DOTA **24.** 31
Scheme S22. Synthesis of DTPA-tetrazine **25** and by-product **26.** 32
Scheme S23. General acylation reaction with DTPA-dianhydride **27.** 34
Scheme S24. Synthesis of PadCTP-AHX-DTPA **3.** 35
Scheme S25. Synthesis of AadUTP-AHX-DTPA **4.** 36
Scheme S26. General acylation reaction with TCO-PEG$_4$-OSu **29** and TCO-PEG$_{12}$-OSu **30.** 37
Scheme S27. Synthesis of PadCTP-PEG$_4$-TCO **31.** 37
Scheme S28. Synthesis of PadCTP-PEG$_{12}$-TCO **32.** 38
Scheme S29. Synthesis of AadUTP-PEG$_4$-TCO **33.** 39
Scheme S30. Synthesis of AadUTP-PEG$_{12}$-TCO **34.** 39
Scheme S31. General strain-promoted alkyne-azide cycloaddition (SPAAC) reaction with DTPA-Tetrazine **25.** 40
Scheme S32. Synthesis of PadCTP-PEG$_4$-DTPA **35.** 40
Scheme S33. Synthesis of PadCTP-PEG$_{12}$-DTPA **36.** 41
Scheme S34. Synthesis of AadUTP-PEG$_{12}$-DTPA **37.** 42
Scheme S35. Synthesis of AadUTP-PEG$_{12}$-DTPA **38.** 43

List of Supplementary Tables

Table S1. Sources of commercially available starting materials and reagents with vendor identifiers, where appropriate. 10
Table S2. List of equipment and consumables used with vendor identifiers, where appropriate. 13
Table S3. List of buffers used with 1X composition. 14
Table S4. List of HPLC methods and columns. 16
Table S5. Summary of radiolabeling conditions. 52
Table S6. Oligonucleotide sequences used in this study. All oligomers were purchased from Integrated DNA Technologies (IDT). 57
Table S7. ICP-MS parameters used. 83
Table S8. ICP-MS data before background subtraction. 84

The remainder of this page is intentionally left blank.

Preamble

Sourcing of Commercial Available Starting Materials

The following commercially available reagents were purchased.

Table S1. Sources of commercially available starting materials and reagents with vendor identifiers, where appropriate.

Reagent		Supplier	Vendor Identifier
Fmoc-AHX-OH	**6**	Ambeed	A162112
PadCTP	**9**	Mason-Chem, or<br>MedChemExpress	MC011927<br>HY-132142
AadUTP	**10**	BOC Sciences	B2001-160513
DOTA(tBu$_3$)-OSu	**13**	Macrocyclics	B-270
DOTA-PEG$_4$-TFP	**16**	Vector Labs (previously Quanta BioDesign)	QBD-11160-100
DOTA-PEG$_{12}$-TFP	**17**	Vector Labs (previously Quanta BioDesign)	QBD-11157-100
DOTA-PEG$_{24}$-TFP	**18**	Vector Labs (previously Quanta BioDesign)	QBD-11158-100
DTPA-Dianhydride	**27**	Millipore Sigma	284025-1G
Tetrazine-NH$_2$ (TFA Salt)	**28**	Ambeed	A721965
TCO-PEG$_4$-OSu	**29**	BroadPharm	BP-22418
TCO-PEG$_{12}$-OSu	**30**	BroadPharm	BP-24123
[^{111}In]-InCl$_3$	-	BWXT (Vancouver, Canada)	-
[^{161}Tb]-TbCl$_3$	-	Either TheThera (Breda, Netherlands) or;<br>Isotopia (Petah Tikva, Israel)	-
[^{177}Lu]-LuCl$_3$	-	Either Eckert & Ziegler (Berlin, Germany) or;<br>ITM (Munich, Germany)	-
[^{68}Ga]-GaCl$_3$	-	From ^{68}Ge generator (ITM, Munich, Germany), and purified via a DGA resin column from Eichrom Technologies LLC (Lisle, IL, USA) per manufacturer instructions.	-
Acetic Acid (AcOH)	-	Millipore Sigma	A6283-2.5L
Acetonitrile (MeCN)	-	Millipore Sigma	34851-4L
Acrylamide: Bisacrylamide (19:1), 40% m/v	-	Bio Basic	A0006
Acrylamide: Bisacrylamide (29:1), 40% m/v	-	Bio Basic	A0007
Ammonium Acetate (NH_4OAc)	-	Fisher Scientific	A637-500
Ammonium Persulfate (APS)	-	Millipore Sigma	A3678-100G
BL21 *E. coli*	-	Generous gift	Katherine Ryan Group, UBC

Reagent		Supplier	Vendor Identifier
Boric Acid	-	Fisher Scientific	A73-500
Bovine Serum Albumin	-	Millipore Sigma	A2153-10G
Bromophenol Blue	-	Millipore Sigma	B0126-25G
Cellulose Dialysis Membrane	-	Millipore Sigma	D9777-100FT
Coomassie Brilliant Blue R250	-	Millipore Sigma	1125530025
Deuterated NMR solvents	-	Either Cambridge Isotope Laboratories or Millipore Sigma	-
Dry Ice (CO_2), nuggets	-	Linde Canada	-
DOTA (free acid)	-	Macrocylics	M-140
Ethanol (EtOH)	-	Millipore Sigma	459836
Ethylenediaminetetraacetic acid (EDTA)	-	Anachemia	37560-300
Formamide	-	ThermoFisher	15515026
Formic Acid (FA)	-	Fisher Scientific	AC423755000
Gallium (III) Chloride ($GaCl_3$)	-	Millipore Sigma	427128
Gallium Standard (ICP)	-	Millipore Sigma	16639
Glycerol	-	Fisher Scientific	G32
Glycine	-	Fisher Scientific	G46-500
HEPES	-	Millipore Sigma	H3375-100G
Hydrochloric Acid (HCl)	-	Millipore Sigma	320331-2.5L
Indium (III) Chloride ($InCl_3$)	-	Ambeed	A715125
Isopropyl β-D-1-thiogalactopyranoside (IPTG)	-	Bio Basic	IB0168
Kanamycin Sulfate	-	Bio Basic	KB0286
Klenow Fragment (3'→5' Exo^{-})	-	New England Biolabs, or; In-house	M0212M (NEB), or; In-house
Lithium Perchlorate ($LiClO_4$), 99.99% Basis	-	Chem-Impex	26479
Luria-Bertani (LB) Broth	-	BD Biosciences	244620
Lutetium (III) Chloride ($LuCl_3$)	-	ThermoFisher	011260.06
Lutetium Standard (ICP)	-	Millipore Sigma	03909
Magnesium Sulfate Hexahydrate ($MgSO_4 \cdot 6H_2O$)	-	Millipore Sigma	M2393-100G
Methanol (MeOH)	-	Millipore Sigma	34860-4X4L-R
N-Hydroxysuccinimide (HOSu)	-	Ambeed	A171441

Reagent		Supplier	Vendor Identifier
N,*N*-Dimethylacetamide (DMAc), AcroSeal anhydrous grade	-	Millipore Sigma	271012-1L
N,*N*-Dimethylformamide (DMF), AcroSeal anhydrous grade	-	Millipore Sigma	227056-1L
N,*N*,*N′*,*N′*-tetramethylethylenediamine (TEMED)	-	ThermoFisher	A12536.AE
Ni Sepharose High Performance column	-	Cytiva	17526801
Phenylmethanesulfonyl Fluoride (PMSF)	-	Bio Basic	PB0425
Phosphate Buffered Saline (PBS)	-	ThermoFisher	10010023
Pierce™ Bradford Protein Assay Kit	-	ThermoFisher	23200
Piperidine	-	Millipore Sigma	104094
Pyrophosphatase, Inorganic from baker's yeast (YIPP)	-	Millipore Sigma	I1643-100UN
Sodium Acetate Trihydrate (NaOAc · $3H_2O$)	-	Fisher Scientific	S209-500
Sodium Chloride (NaCl)	-	Millipore Sigma	S9888-2.5KG
Sodium Dodecyl Sulfate (SDS)	-	Millipore Sigma	L3771-100G
Sodium Hydroxide (NaOH)	-	Fisher Scientific	S318-500
Streptavidin Magnetic Beads	-	New England Biolabs	S1420S
Terbium (III) Chloride ($TbCl_3$)	-	ThermoFisher	044472.06
Terminal Transferase	-	New England Biolabs	M0315L
Triethylamine (TEA)	-	Millipore Sigma	8083521000
Trifluoroacetic Acid (TFA)	-	Millipore Sigma	T6508
TRIS · HCl	-	Ambeed	A104132
TRIS (base)	-	Fisher Scientific	BP152-1
TRIS-Borate-EDTA (TBE)	-	Bio Basic	A0024
Urea	-	Bio Basic	UB0148
Xylene Cyanol FF	-	Millipore Sigma	X4126-10G
Yttrium (III) Chloride (YCl_3)	-	Alfa Aesar	011184.14
Yttrium Standard (ICP)	-	Millipore Sigma	01357
β-mercaptoethanol (β-ME)	-	Thermo Fisher	A158900B
Sodium Dodecyl Sulfate (SDS)	-	Millipore Sigma	L3771-100G

Reagent		Supplier	Vendor Identifier
Sodium Hydroxide (NaOH)	-	Fisher Scientific	S318-500
Streptavidin Magnetic Beads	-	New England Biolabs	S1420S
Terbium (III) Chloride ($TbCl_3$)	-	ThermoFisher	044472.06
Terminal Transferase	-	New England Biolabs	M0315L
Triethylamine (TEA)	-	Millipore Sigma	8083521000
Trifluoroacetic Acid (TFA)	-	Millipore Sigma	T6508
TRIS · HCl	-	Ambeed	A104132
TRIS (base)	-	Fisher Scientific	BP152-1
TRIS-Borate-EDTA (TBE)	-	Bio Basic	A0024
Urea	-	Bio Basic	UB0148
Xylene Cyanol FF	-	Millipore Sigma	X4126-10G
Yttrium (III) Chloride (YCl_3)	-	Alfa Aesar	011184.14
Yttrium Standard (ICP)	-	Millipore Sigma	01357
β-mercaptoethanol (β-ME)	-	Thermo Fisher	A158900B

Equipment and Consumables

Equipment and consumables were purchased from the following manufacturers.

Table S2. List of equipment and consumables used with vendor identifiers, where appropriate.

Name	Company	Model Name/ Number	City
Biomolecular Imager/ Phosphorimager	Cytiva	Typhoon™ 5 (29187191)	Marlborough, MA, USA
DNAPac™ PA-100 HPLC Column	Thermo Fisher	043010	Waltham, MA, USA
Eclipse XDB-C18 HPLC Column	Agilent	990967-202	Santa Clara, CA, USA
High Performance Liquid Chromatography (HPLC)	Agilent	Either of 1100, 1200, or 1260 with OpenLab CDS operating and analysis software. For the 1260 machine for radio-HPLC, it is equipped with a Bioscan (Washington, DC, USA) NaI scintillation detector.	Santa Clara, CA, USA
Inductively Coupled Plasma-Mass Spectrometry (ICP-MS)	Agilent	7850 ICP-MS (G8422A) equipped with an autosampler SPS4 and analysis software MassHunter 5.1 (Agilent G7201D, version D01.01, Build 653.5)	Santa Clara, CA, USA
Jupiter 10 µm C18 HPLC Column	Phenomenex	00G-4055-E0	Torrance, CA, USA

Name	Company	Model Name/ Number	City
Mass Spectrometer (MS) Electrospray Ionization-Time of Flight (ESI-TOF)	Waters	2695 Separation module with Waters-Micromass ZQ	Framingham, MA, USA
Mass Spectrometer (MS) High Capacity Ion Trap (HCT)	Bruker	High Capacity Ion Trap (HCT)	Billerica, MA, USA
Mini-PROTEAN® Gel Cassette System	Bio-Rad	1658005EDU	Hercules, CA, USA
Nalgene™ Rapid-Flow™ Sterile Disposable Bottle Top Filters with PES	Thermo Fisher	597-4520	Waltham, MA, USA
Nuclear Magnetic Resonance (NMR) Spectrophotometers	Bruker	Avance series equipped with a BACS-120 autosampler	Billerica, MA, USA
Scintillation Counter	Revvity	Wizard2 2480 automatic gamma counter	Waltham, MA, USA
SepPAK Cartridge, C_{18}, 5 g sorbent	Waters	WAT036925	Framingham, MA, USA
SepPAK Cartridge, C_{18}, 130 mg sorbent	Waters	WAT023501	Framingham, MA, USA
Thermocycler	Bio-Rad	T100	Hercules, CA, USA
UV-Vis Spectrophotometer	Molecular Devices	SpectraMax QuickDrop	San Jose, CA, USA

Buffer Formulations

Unless otherwise specified, the following buffers were produced in house.

Table S3. List of buffers used with 1X composition.

Name	Label	1X Composition
DNA Native Loading Buffer/ Strand Displacement Buffer	**B-1**	20 mM TRIS (pH 7.6), 10 mM $MgSO_4$, 50 mM NaCl, 15% v/v Glycerol
Triethylammonium Bicarbonate (TEAB)	**B-2**	50 mM Triethylammonium Bicarbonate
Terminal Transferase Buffer (New England Biolabs)	**B-3**	50 mM KOAc, 20 mM TRIS-OAc, 10 mM $Mg(OAc)_2$ (pH 7.9 at 25°C)
NEBuffer 2 (New England Biolabs)	**B-4**	50 mM NaCl, 10 mM TRIS-HCl, 10 mM $MgCl_2$, 1 mM DTT, pH 7.9
DNA Denaturing Loading Buffer	**B-5**	10 mM EDTA (aq., 2% v/v), 0.1% m/v Bromophenol Blue in 98% v/v formamide
DNA Denaturing Running Buffer (identical to 1X TBE)	**B-6**	100 mM TRIS (pH 8.3), 100 mM Boric Acid, 2 mM EDTA
DNA Gel Elution Buffer	**B-7**	10 mM TRIS (pH 8.0), 1 mM EDTA, 50 mM NaCl

Name	Label	1X Composition
DNA Native Running Buffer	**B-8**	100 mM TRIS (pH 8.3), 100 mM Boric Acid
Protein Denaturing Gel Separating Buffer	**B-9**	1.5 M TRIS-HCl (pH 8.8)
Protein Denaturing Gel Stacking Buffer	**B-10**	500 mM TRIS-HCl (pH 6.8)
Protein Denaturing Loading Buffer	**B-11**	60 mM TRIS-HCl (pH 6.8), 2% m/v SDS, 10% v/v Glycerol, 5% v/v β-ME, 0.01% m/v Bromophenol Blue
Protein Denaturing Running Buffer	**B-12**	25 mM TRIS (pH 8.3), 192 mM Glycine, 0.1% m/v SDS
Protein Gel Staining Solution	**B-13**	0.1% m/v Coomassie Brilliant Blue R-250, 40% v/v Methanol, 10% v/v Acetic Acid
Protein Gel Destaining Solution	**B-14**	40% v/v Methanol, 10% v/v Acetic Acid, 50% v/v Water
Protein Elution Buffer	**B-15**	11.6 mM Na_2HPO_4, 8.4 mM $NaH_2PO_4 \cdot H_2O$, 500.0 mM NaCl
Protein Dialysis Buffer	**B-16**	25 mM Tris-HCl, 0.1 mM EDTA, pH 7.4
DNA Oligonucleotide ($LiClO_4$) weak HPLC solvent	**B-17**	25 mM TRIS (pH 8.5) in 15% MeCN in milliQ-filtered H_2O
DNA Oligonucleotide ($LiClO_4$) strong HPLC solvent	**B-18**	25 mM TRIS (pH 8.5), 200 mM $LiClO_4$, in 15% MeCN in milliQ-filtered H_2O

For preparation of triethylammonium bicarbonate (TEAB) solution (1.0 M, 1.0 L, at 20X **B-2**), triethylamine (TEA, 139 mL) was mixed with dH_2O (800 mL) in a three-neck RBF over an ice bath to obtain a biphasic mixture under rapid stirring. In a separate sealed container with subliming dry ice (Linde Canada), gaseous CO_2 generated was introduced to the biphasic mixture via tubing connected to a bubbler. After the mixture was homogenized, the bubbling continued until pH 8 was reached. The solution was then topped up to 1.0 L with dH_2O, and filtered using Nalgene™ Rapid-Flow™ Sterile Disposable Bottle Top Filters (ThermoFisher 597-4520). For long term storage, the solution was aliquoted in 50 mL fractions and frozen in –20ºC. Prior to usage for HPLC analysis, a 50 mL aliquot was diluted to 1.0 L to afford a final working concentration of 50 mM as the aqueous strong mobile phase A. The organic weak mobile phase B was MeCN for the purpose of nucleotide triphosphate separation.

Terminal Transferase Buffer **B-3** (NEB B0315) and NEBuffer 2 **B-4** (NEB B7202) were purchased as 10X solutions from New England Biolabs (NEB). Production for DNA HPLC solvents **B-17** and **B-18** in preparation for ICP-MS was described in **Anionic Exchange HPLC of Oligonucleotides for ICP-MS Analysis d**. The remaining buffers were produced in-house at the described concentrations in milliQ H_2O, unless otherwise specified.

Methods for High Performance Liquid Chromatography (HPLC)

All HPLC solvents involving triethylammonium bicarbonate for purification of nucleotide triphosphates, its preparation was disclosed in the **B-2** recipe. For solvents **B-17–B-18** for DNA

oligonucleotides resolution in preparation for ICP-MS analysis, please consult **Anionic Exchange HPLC of Oligonucleotides for ICP-MS Analysis d**. HPLC Methods were summarized as following.

Table S4. List of HPLC methods and columns.

Method	Condition	Column
**M-1**	Solvent A, 50 mM triethylammonium bicarbonate (TEAB) in milliQ-filtered H_2O; solvent B, MeCN; 0.0–15.0 min, 0–75% B; 15.0–15.5 min, 75–80% B; 15.5–21.5 min, 80% B; 21.5–22.0 min, 80–0% B; 22.0–28.0 min, 0% B; flow rate, 2.0 mL/min; column temperature, 19ºC – 21ºC.	Eclipse XDB-C18 5 μm, 250 × 9.4 mm
**M-2**	Solvent A, 50 mM triethylammonium bicarbonate (TEAB) in milliQ-filtered H_2O; solvent B, MeCN; 0.0–10.0 min, 0–60% B; 10.0–10.5 min, 60–80% B; 10.5–12.5 min, 80% B; 12.5–13.0 min, 80–0% B; 13.0–15.0 min, 0% B; flow rate, 15.0 mL/min; column temperature, 19ºC – 21ºC.	Jupiter 10 μm C18 300 Å, 250 × 4.6 mm
**M-3**	Solvent A, 50 mM triethylammonium bicarbonate (TEAB) in milliQ-filtered H_2O; solvent B, MeCN; 0.0–20.0 min, 0–15% B; 20.0–20.5 min, 15–80% B; 20.5–22.5 min, 80% B; 22.5–23.0 min, 80–0% B; 23.0–25.0 min, 0% B; flow rate, 2.0 mL/min; column temperature, 19ºC – 21ºC.	Eclipse XDB-C18 5 μm, 250 × 9.4 mm
**M-4**	Solvent A, 50 mM triethylammonium bicarbonate (TEAB) in milliQ-filtered H_2O; solvent B, MeCN; 0.0–10.0 min, 0–15% B; 10.0–10.5 min, 15–80% B; 10.5–12.5 min, 80% B; 12.5–13.0 min, 80–0% B; 13.0–15.0 min, 0% B; flow rate, 2.0 mL/min; column temperature, 19ºC – 21ºC.	Eclipse XDB-C18 5 μm, 250 × 9.4 mm
**M-5**	Solvent A, 50 mM triethylammonium bicarbonate (TEAB) in milliQ-filtered H_2O; solvent B, MeCN; 0.0–10.0 min, 0–80% B; 10.0–10.5 min, 80–80% B; 10.5–12.5 min, 80% B; 12.5–13.0 min, 80–0% B; 13.0–15.0 min, 0% B; flow rate, 2.0 mL/min; column temperature, 19ºC – 21ºC.	Eclipse XDB-C18 5 μm, 250 × 9.4 mm
**M-6**	Solvent A, 0.1 % formic acid (% FA) in milliQ-filtered H_2O; solvent B, MeCN with 0.1% FA; 0.0–3.0 min, 0–60% B; 3.0–3.5 min, 60–100% B; 3.5–5.0 min, 100% B; flow rate, 15.0 mL/min; column temperature, 19ºC – 21ºC.	Agilent Prep 100Å 5 μm, C18, 21.2 × 50 mm
**M-7**	Solvent A, 50 mM triethylammonium bicarbonate (TEAB) in milliQ-filtered H_2O; solvent B, MeCN; 0.0–20.0 min, 5–20% B; 20.0–20.5 min, 20–100% B; 20.5–22.5 min, 100% B; 22.5–23.0 min, 100–5% B; 23.0–27.0 min, 5% B; flow rate, 1.0 mL/min; column temperature, 19ºC – 21ºC.	Jupiter 10 μm C18 300 Å, 250 × 4.6 mm
**M-8**	Solvent A, 50 mM triethylammonium bicarbonate (TEAB) in milliQ-filtered H_2O; solvent B, MeCN; 0.0–10.0 min, 5–75% B; 10.0–10.5 min, 75–100% B; 10.5–12.5 min, 100% B; 12.5–13.0 min, 100–5% B; 13.0–16.0 min, 5% B; flow rate, 2.0 mL/min; column temperature, 19ºC – 21ºC.	Jupiter 10 μm C18 300 Å, 250 × 4.6 mm

Method	Condition	Column
**M-9**	Solvent A, 50 mM triethylammonium bicarbonate (TEAB) in milliQ-filtered H_2O; solvent B, MeCN; 0.0–10.0 min, 5–20% B; 10.0–10.5 min, 20–100% B; 10.5–12.5 min, 100% B; 12.5–13.0 min, 100–5% B; 13.0–16.0 min, 5% B; flow rate, 2.0 mL/min; column temperature, 19ºC – 21ºC.	Jupiter 10 μm C18 300 Å, 250 × 4.6 mm
**M-10**	Solvent A, 50 mM triethylammonium bicarbonate (TEAB) in milliQ-filtered H_2O; solvent B, MeCN; 0.0–10.0 min, 0–15% B; 10.0–10.5 min, 15–80% B; 10.5–12.5 min, 80% B; 12.5–13.0 min, 80–0% B; 13.0–15.0 min, 0% B; flow rate, 2.0 mL/min; column temperature, 19ºC – 21ºC.	Jupiter 10 μm C18 300 Å, 250 × 4.6 mm
**M-11**	Solvent A, 25 mM TRIS (pH 8.50) in 15% MeCN in milliQ-filtered H_2O; solvent B, 25 mM TRIS (pH 8.50), 200 mM $LiClO_4$, in 15% MeCN in milliQ-filtered H_2O; 0.0–7.0 min, 40–61% B; 7.0–15.0 min, 61–74% B; 15.0–15.5 min, 74–75% B; 15.5–18.5 min, 75% B; 18.5–19.0 min, 75–40% B; 19.0–22.0 min, 40% B; flow rate, 0.5 mL/min; column temperature, 19ºC – 21ºC.	DNAPac^{TM} PA-100, BioLC 4 × 250 mm

Gel Image Presentation

Gel images were contrast-adjusted using Fiji (software Version 2.16.0/1.54p build 26d66057dd).[29] Figures were then assembled with LabFigures (https://labfigures.com/).

Chemical Synthesis

A. Synthesis of XadYTP-AHX-DOTA Series

Fmoc-AHX-OSu **5**

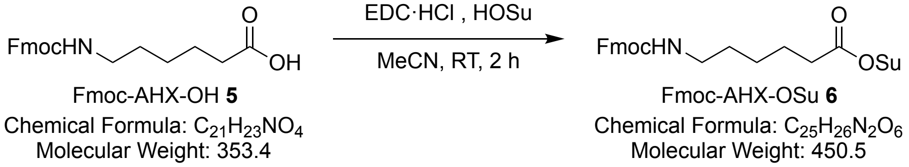

Scheme S1. Synthesis of Fmoc-AHX-OSu **5**.

To a flame dried 50 mL RBF under Ar atmosphere, Fmoc-AHX-OH (**6**, 51.8 mg, 141 μmol, R_f = 0.31 with streaking, 50% EtOAc/Hexanes), *N*-hydroxysuccinimide (HOSu, 22.1 mg, 178 μmol, 1.3 eq.), MeCN (6.0 mL, dried by stills) was first dissolved and finally added EDC·HCl (37.6 mg, 182 μmol, 1.3 eq.), and reacted quantitatively in 17 h in RT as evidenced by TLC. The crude mixture was concentrated *in vacuo* and resuspended in CH_2Cl_2 (10 mL), and washed against H_2O (10 mL × 2) and sat. NaCl (aq., 10 mL) by liquid-liquid extraction. The recovered organic layer was dried over anhy. $MgSO_4$ to obtain Fmoc-AHX-OSu (**5**, 65.5 mg, quant.) as a colorless oil. R_f = 0.40, 50% EtOAc/Hexanes. ^{1}H NMR (300 MHz, CD_2Cl_2) δ 7.79 (d, *J* = 7.5 Hz, 2H), 7.63 (d, *J* = 7.4 Hz, 2H), 7.41 (t, *J* = 7.4 Hz, 2H), 7.33 (t, *J* = 7.4 Hz, 2H), 5.09 (t, *J* = 5.9 Hz, 1H), 4.38 (d, *J* = 6.9 Hz, 2H), 4.22 (t, *J* = 6.9 Hz, 1H), 3.17 (q, *J* = 6.5 Hz, 2H), 2.78 (s, 4H), 2.61 (t, *J* = 7.3 Hz, 2H), 1.75 (p, *J* = 7.4 Hz, 2H), 1.61 – 1.45 (m, 2H), 1.45 – 1.36 (m, 2H). ^{13}C NMR (75 MHz, CD_2Cl_2) δ 169.75, 169.05, 156.60, 144.51, 141.58, 127.93, 127.33, 125.42,

120.23, 66.56, 47.63, 40.89, 31.12, 29.63, 26.01, 25.94, 24.56. ESI-TOF calcd. [$C_{25}H_{26}N_2O_6$ + Na]$^+$ 473.1, found 473.2. λ_{max} = 301 nm.

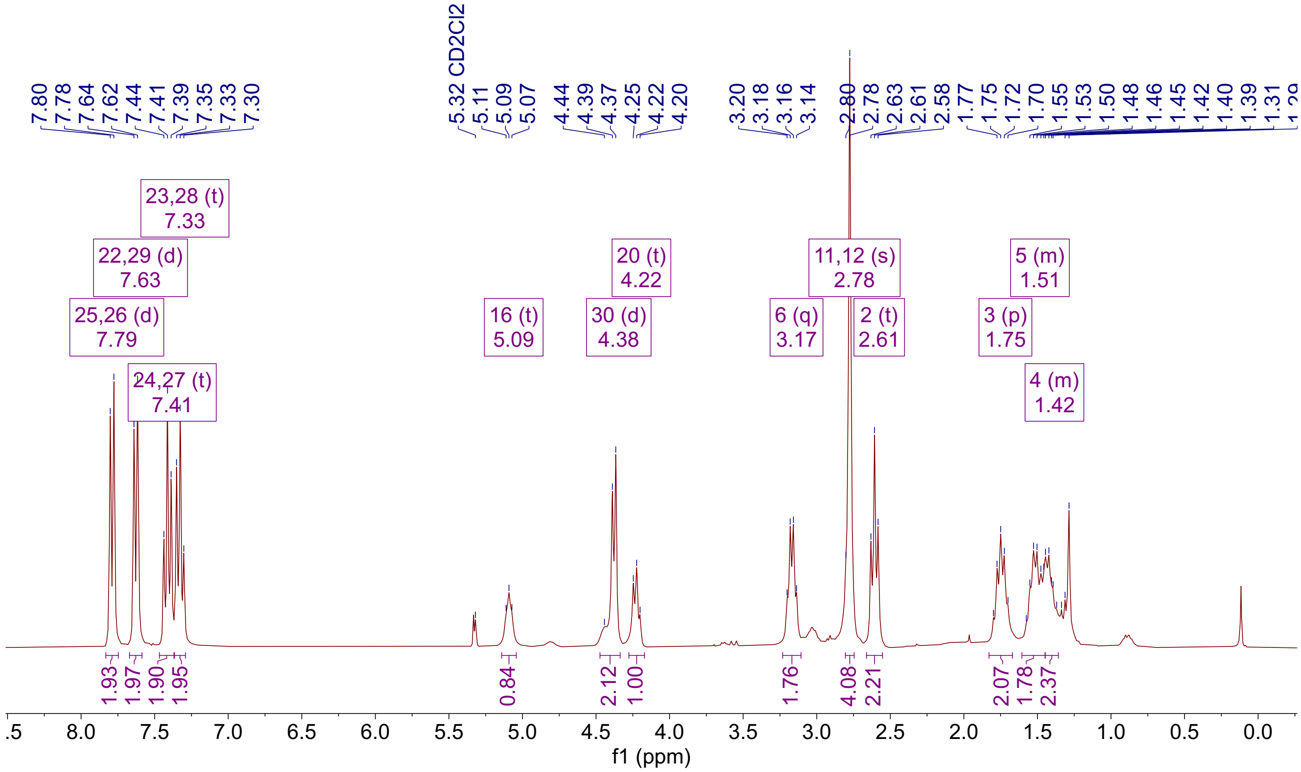

Figure S1. ^{1}H NMR spectrum of Fmoc-AHX-OSu **5**.

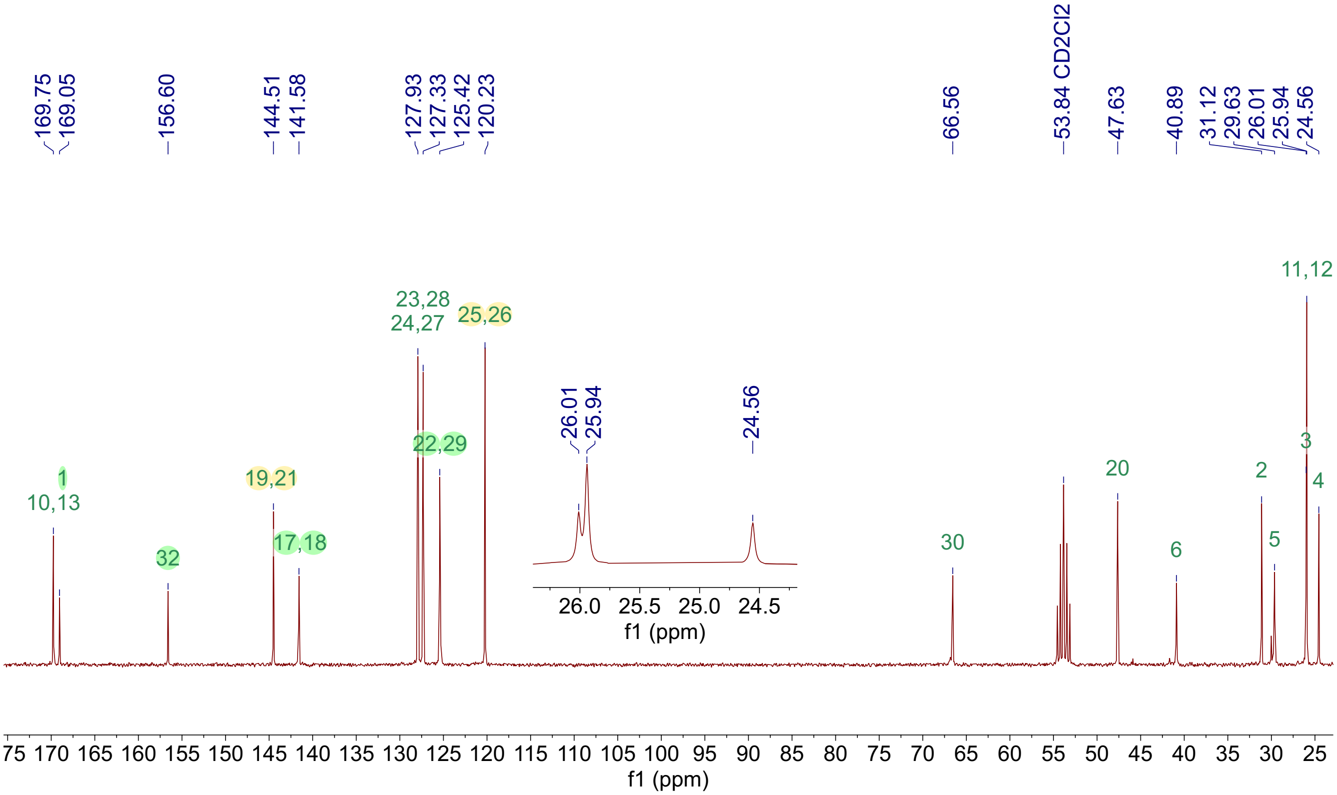

Figure S2. ^{13}C NMR spectrum of Fmoc-AHX-OSu **5**.

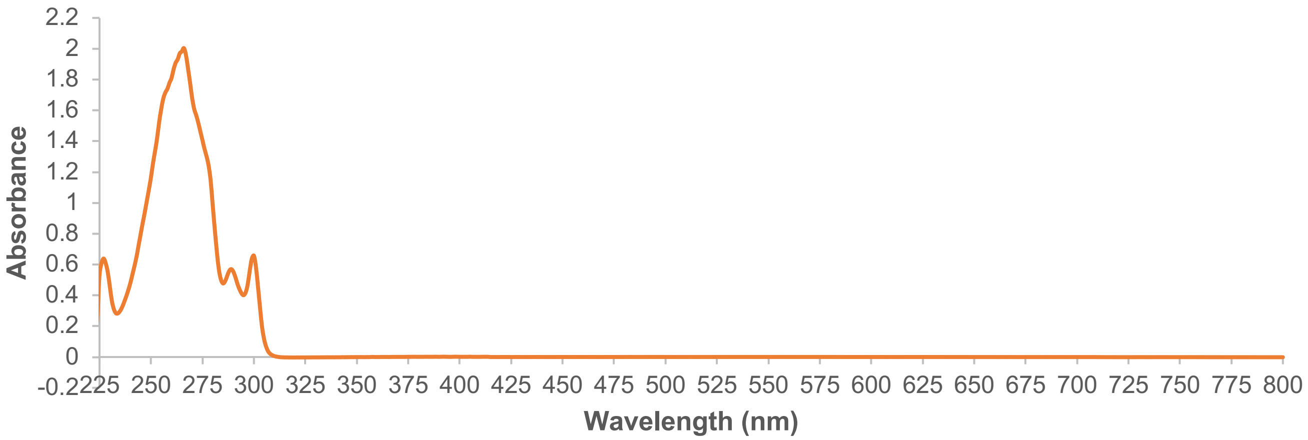

Figure S3. UV-Vis spectrum of Fmoc-AHX-OSu **5**.

Extinction coefficient of the Fmoc group is taken as ε_{290} = 6089 M^{-1} cm^{-1}.[30] The same reference also suggests ε_{304} = 8021 M^{-1} cm^{-1}. ε_{290} = 6089 M^{-1} cm^{-1} will be carried to estimate extinction coefficients of Fmoc-protected nucleotide triphosphates **7** and **8**.

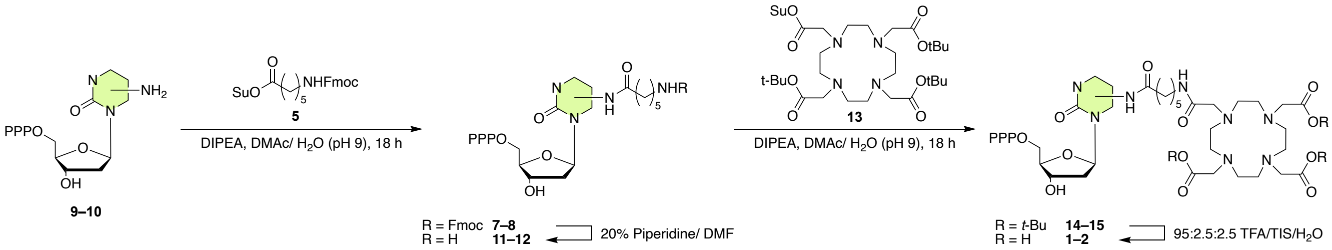

Scheme S2. General synthesis strategy for **1** and **2**. PPPO refers to 5′-*O*-triphosphate.

General acylation step with Fmoc-AHX-OSu **5**

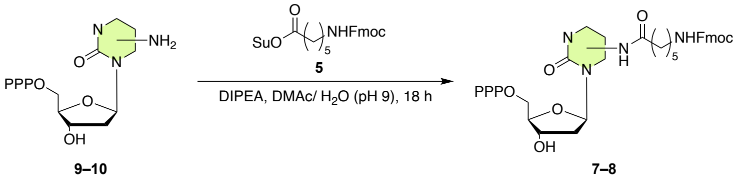

Scheme S3. General acylation reaction with Fmoc-AHX-OSu **5**.

To a 1.5 mL microcentrifuge Tube, DIPEA (17 µL, 97 µmol, 5.1 eq.) was introduced to an aqueous solution of nucleotide triphosphate (**9, 10**, 19 µmol in 100 µL ddH_2O), following Fmoc-AHX-OSu (**5**, 95 µmol, 5.0 eq., 186 µL, 519 mM in DMAc). The cloudy solution was diluted in DMAc and/or ddH_2O to completely dissolve the precipitates (as per specified below). After complete dissolution, its pH was tested to be 9, and was allowed to react for 18 h in RT, followed by solvent removal *in vacuo* in a Centrivap Evaporator. The dried white powder was resuspended in ddH_2O (500 µL) and acetone (500 µL) to give a clear solution, and then precipitated in $LiClO_4$ (10 mL, 1% *w/v* in acetone) at –78ºC for 10 min. After centrifugation, the pellet was further washed with fresh $LiClO_4$ (10 mL × 1, 1% *w/v* in acetone). The washed pellet was chromatographed via HPLC to yield product (**7, 8**) as white powders that are very water-soluble.

HPLC Method **M-1**: Solvent A, 50 mM triethylammonium bicarbonate (TEAB) in milliQ-filtered H_2O; solvent B, MeCN; 0.0–15.0 min, 0–75% B; 15.0–15.5 min, 75–80% B; 15.5–21.5 min, 80% B; 21.5–22.0 min, 80–0% B; 22.0–28.0 min, 0% B; flow rate, 2.0 mL/min; column temperature, 19ºC – 21ºC.
HPLC Column: Eclipse XDB-C18 5 μm, 250 × 9.6 mm.

PadCTP-AHX-Fmoc ***7***

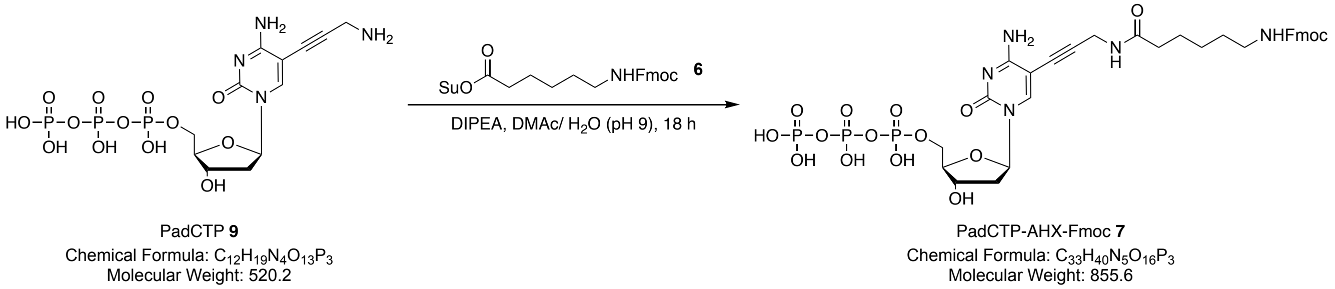

Scheme S4. Synthesis of PadCTP-AHX-Fmoc **7**.

PadCTP (**9**, 19 μmol in 100 μL ddH_2O) was used for this step, and the reaction mixture required further dilution (DMAc, 400 μL) to completely dissolve all particulates. t_R = 13.2 min. **7** was obtained as a fine white powder (5.2 μmol, 27%). λ_{max} = 294 nm, 301 nm.

AadUTP-AHX-Fmoc ***8***

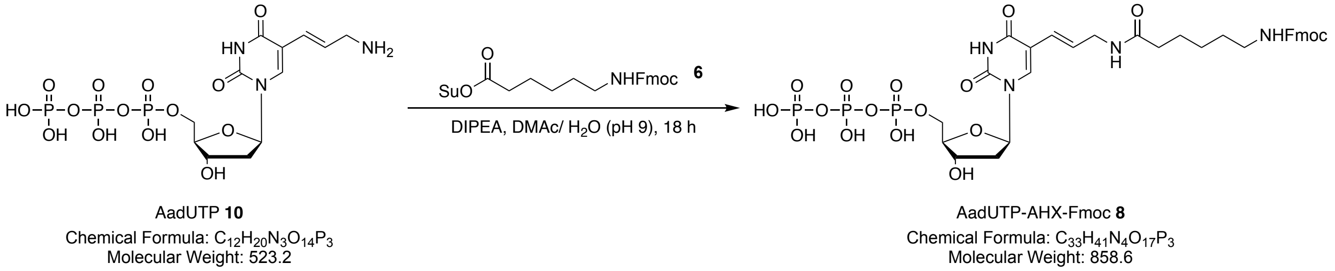

Scheme S5. Synthesis of AadUTP-AHX-Fmoc **8**.

AadUTP (**10**, 19 μmol in 100 μL ddH_2O) was used for this step, and the reaction mixture required further dilution (DMAc, 4.5 mL and ddH_2O, 2.0 mL) to completely dissolve all particulates. t_R = 12.6 min. **8** was obtained as a fine white powder (5.7 μmol, 30%). λ_{max} = 289 nm, 301 nm.

General Fmoc deprotection

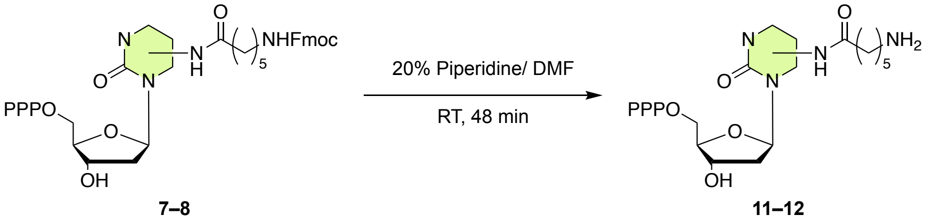

Scheme S6. General Fmoc-removal in 20% (v/v) piperidine/ DMF.

To a 15 mL conical tube, dYTP-AHX-Fmoc (**7, 8**) was completely dissolved in DMF (400 µL), followed by introduction of piperidine (100 µL). The reaction reacted for 48 min in RT, and the solvent was removed *in vacuo* by a Centrivap Evaporator. The dried white powder was resuspended in ddH_2O (500 µL) and acetone (500 µL) to give a clear solution, and then precipitated in $LiClO_4$ (10 mL, 1% *w/v* in acetone) at –78ºC for 10 min. After centrifugation, the pellet was further washed with fresh $LiClO_4$ (10 mL × 1, 1% *w/v* in acetone). The dried pellet product (**11, 12**) was directly used in the next step without further purification.

PadCTP-AHX-NH₂ ***11***

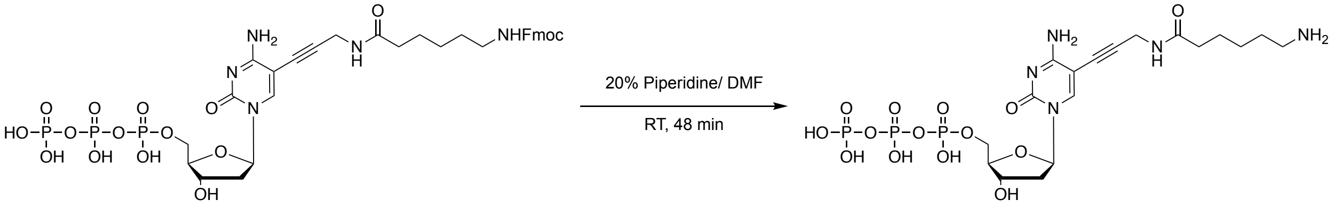

PadCTP-AHX-Fmoc **7**
Chemical Formula: $C_{33}H_{40}N_5O_{16}P_3$
Molecular Weight: 855.6

PadCTP-AHX-NH_2 **11**
Chemical Formula: $C_{18}H_{30}N_5O_{14}P_3$
Molecular Weight: 633.4

Scheme S7. Synthesis of PadCTP-AHX-NH_2 **11**.

PadCTP-AHX-Fmoc (**7**, 5.2 µmol) was the starting material of this reaction, yielding 5.2 µmol product (**11**, Quant.) ESI-TOF calcd. $[C_{18}H_{30}N_5O_{14}P_3 + H]^+$ 634.1, found 634.5. λ_{max} = 294 nm.

AadUTP-AHX-NH₂ ***12***

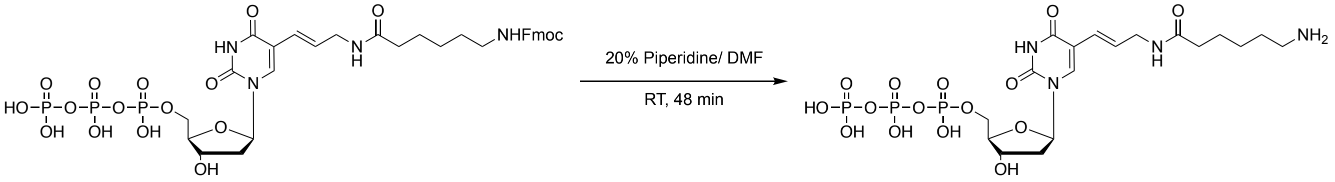

AadUTP-AHX-Fmoc **8**
Chemical Formula: $C_{33}H_{41}N_4O_{17}P_3$
Molecular Weight: 858.6

AadUTP-AHX-NH_2 **12**
Chemical Formula: $C_{18}H_{31}N_4O_{15}P_3$
Molecular Weight: 636.4

Scheme S8. Synthesis of AadUTP-AHX-NH_2 **12**.

AadUTP-AHX-Fmoc (**8**, 5.7 µmol) was the starting material of this reaction, yielding 5.6 µmol product (**12**, Quant.) ESI-TOF calcd. $[C_{18}H_{31}N_4O_{15}P_3 + H]^+$ 636.1, found 635.3. λ_{max} = 289 nm.

General acylation step with DOTA-mono-NHS tris (*t*-Bu ester) **13**

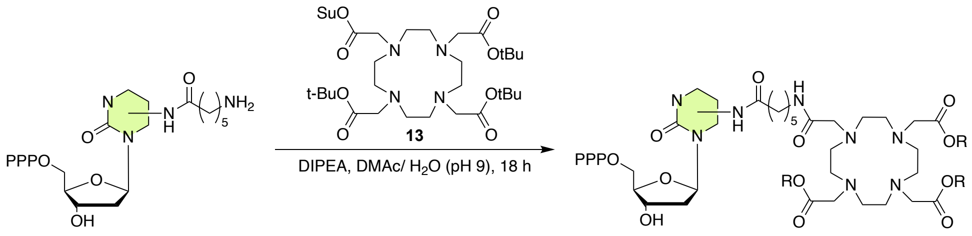

11–12 **13–14**

Scheme S9. General acylation reaction with DOTA(tBu)$_3$-OSu **13**.

Fresh aliquots of dYTP-AHX-DOTA (**11**, **12**, 2.0 µmol) in 1.5 mL Eppendorf Tubes was resuspended in ddH_2O (5.0 µL) and DIPEA (1.5 µL, 16 µmol, 7.8 eq.). DOTA(tBu)$_3$-OSu·HPF_6 (**13**, 5.6 µmol, 2.8 eq., 15.0 µL, 368 mM in DMAc) was added, and the mixture was sonicated to dissolve all particulates. The clear colorless solution was allowed to react at 37ºC for 18 h, following solvent removal *in vacuo* in a Centrivap Evaporator. The white pellet was extracted with ddH_2O and the supernatant was chromatographed via HPLC to obtain products (**14**, **15**) as white powders that are very water-soluble.

HPLC Method **M-2**: Solvent A, 50 mM triethylammonium bicarbonate (TEAB) in milliQ-filtered H_2O; solvent B, MeCN; 0.0–10.0 min, 0–60% B; 10.0–10.5 min, 60–80% B; 10.5–12.5 min, 80% B; 12.5–13.0 min, 80–0% B; 13.0–15.0 min, 0% B; flow rate, 15.0 mL/min; column temperature, 19ºC – 21ºC.
HPLC Column: Jupiter 10 µm C18 300A, 250 × 4.6 mm.

PadCTP-AHX-DOTA(tBu)$_3$ ***14***

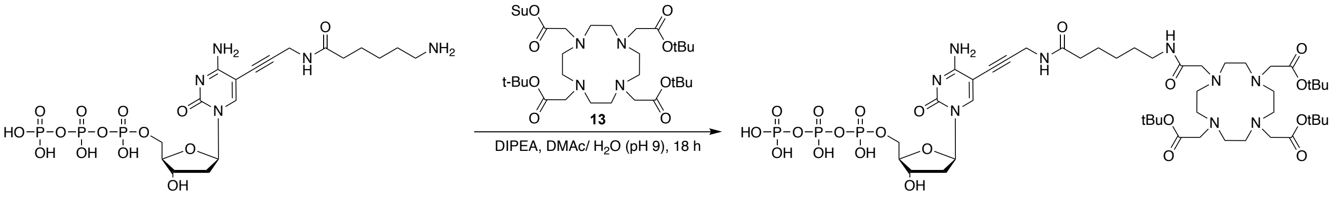

PadCTP-AHX-NH$_2$ **11**
Chemical Formula: $C_{18}H_{30}N_5O_{14}P_3$
Molecular Weight: 633.4

PadCTP-AHX-DOTA(tBu)$_3$ **13**
Chemical Formula: $C_{46}H_{80}N_9O_{21}P_3$
Molecular Weight: 1188.1

Scheme S10. Synthesis of PadCTP-AHX-DOTA(tBu)$_3$ **14**.

PadCTP-AHX-NH$_2$ (**11**, 2.0 µmol) was the starting material of this reaction, yielding product **14** (1.5 µmol, 76%). t_R = 9.6 min. ESI-TOF calcd. $[C_{46}H_{80}N_9O_{21}P_3 + H]^+$ 1188.5, found 1188.7. λ_{max} = 294 nm.

AadUTP-AHX-DOTA(tBu)$_3$ ***15***

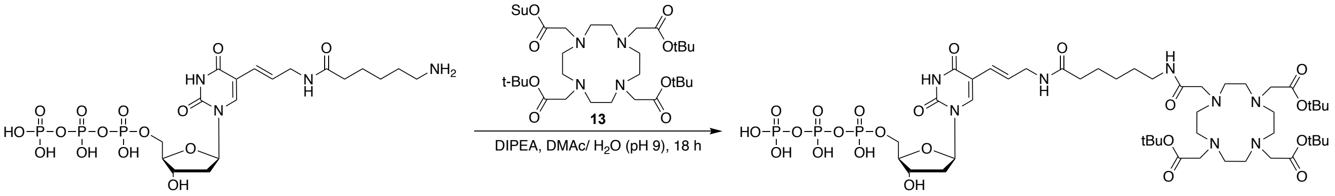

AadUTP-AHX-NH$_2$ **12**
Chemical Formula: $C_{18}H_{31}N_4O_{15}P_3$
Molecular Weight: 636.4

AadUTP-AHX-DOTA(tBu)$_3$ **14**
Chemical Formula: $C_{46}H_{81}N_8O_{22}P_3$
Molecular Weight: 1191.1

Scheme S11. Synthesis of AadUTP-AHX-DOTA(tBu)$_3$ **15**.

AadUTP-AHX-NH$_2$ (**12**, 2.0 µmol) was the starting material of this reaction, yielding product **15** (1.6 µmol, 81%). t_R = 9.6 min. ESI-TOF calcd. $[C_{46}H_{80}N_9O_{21}P_3 + H]^+$ 1191.5, found 1191.6. λ_{max} = 289 nm.

General deprotection with TFA

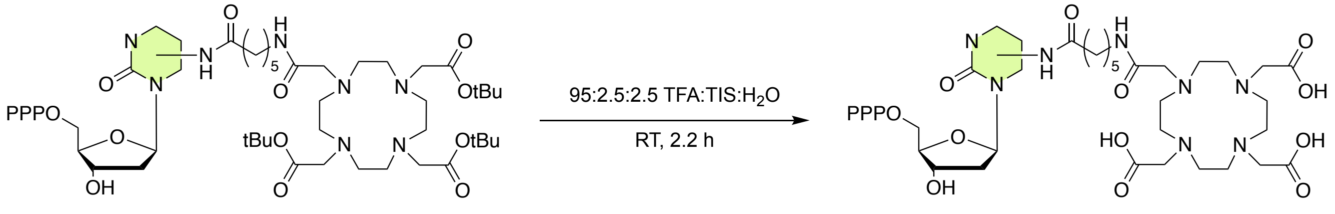

13–14 **1–2**

Scheme S12. General *tert*-butyl ester removal by 95:2.5:2.5 TFA/TIS/H_2O.

To a 15 mL conical tube, dYTP-AHX-DOTA(tBu)$_3$ (**14**, **15**) was completely dissolved in TFA:TIS:H_2O (95:2.5:2.5 *v/v/v*, 500 µL) and allowed to react for 2.5 h. The crude mixture was directly triturated in Et_2O (5 mL × 3, incubated at –78ºC for 10 min). The air-dried pellet was resuspended in 100 µL ddH_2O and purified via HPLC. Product containing fractions (**1**, **2**) were lyophilized to dryness, resuspended in ddH_2O for UV-Vis quantification.

HPLC Method **M-3**: Solvent A, 50 mM triethylammonium bicarbonate (TEAB) in milliQ-filtered H_2O; solvent B, MeCN; 0.0–20.0 min, 0–15% B; 20.0–20.5 min, 15–80% B; 20.5–22.5 min, 80% B; 22.5–23.0 min, 80–0% B; 23.0–25.0 min, 0% B; flow rate, 15.0 mL/min; column temperature, 19ºC – 21ºC.
HPLC Column: Jupiter 10 µm C18 300A, 250 × 4.6 mm.

PadCTP-AHX-DOTA ***1***

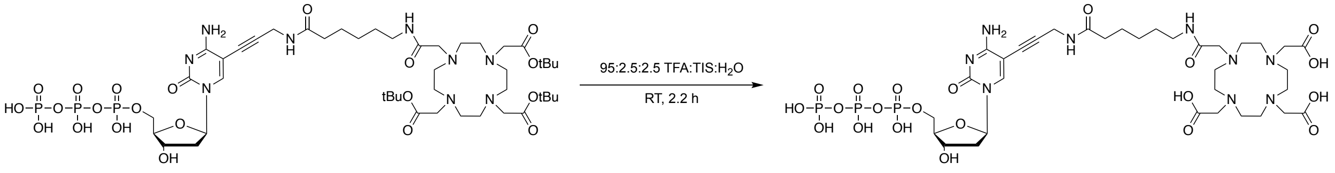

PadCTP-AHX-DOTA(tBu)$_3$ **13**
Chemical Formula: $C_{46}H_{80}N_9O_{21}P_3$
Molecular Weight: 1188.1

PadCTP-AHX-DOTA **1**
Chemical Formula: $C_{34}H_{56}N_9O_{21}P_3$
Molecular Weight: 1019.8

Scheme S13. Synthesis of PadCTP-AHX-DOTA **1**.

PadCTP-AHX-DOTA(tBu)$_3$ (**14**, 1.5 µmol) was the starting material of this reaction, yielding 761 nmol product (**1**, 52%). t_R = 8.9 min. ^{31}P NMR (202 MHz, D_2O) δ -10.42 – -11.09 (m), -11.27 – -12.06 (m), -23.20 (t). ESI-TOF calcd. $[C_{34}H_{56}N_9O_{21}P_3 + H]^+$ 1020.3, found 1020.2. λ_{max} = 294 nm.

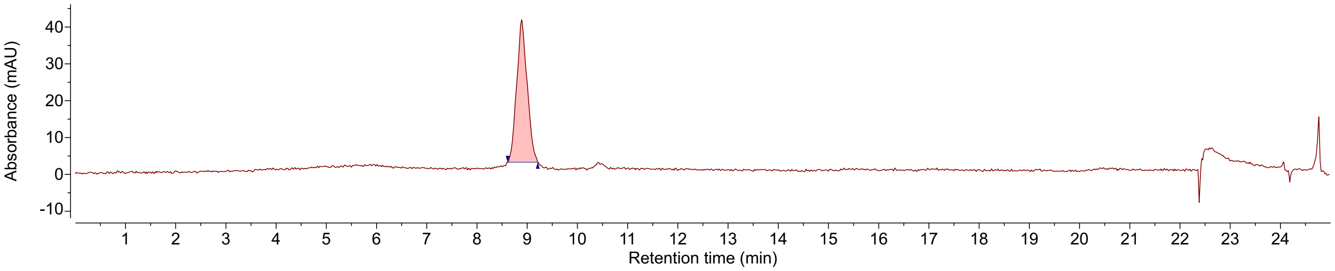

Figure S4. HPLC chromatogram of PadCTP-AHX-DOTA **1**.

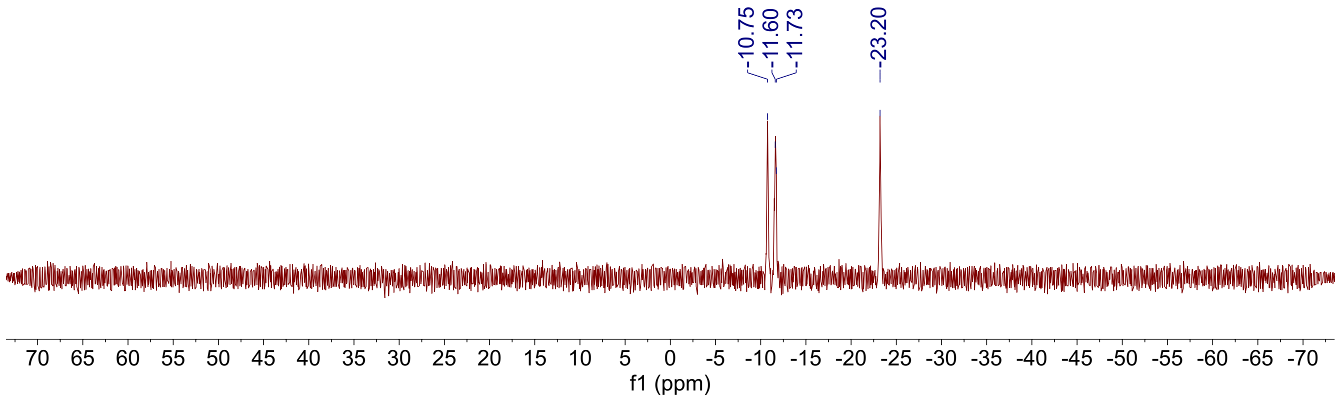

Figure S5. ^{31}P NMR spectrum of PadCTP-AHX-DOTA **1**.

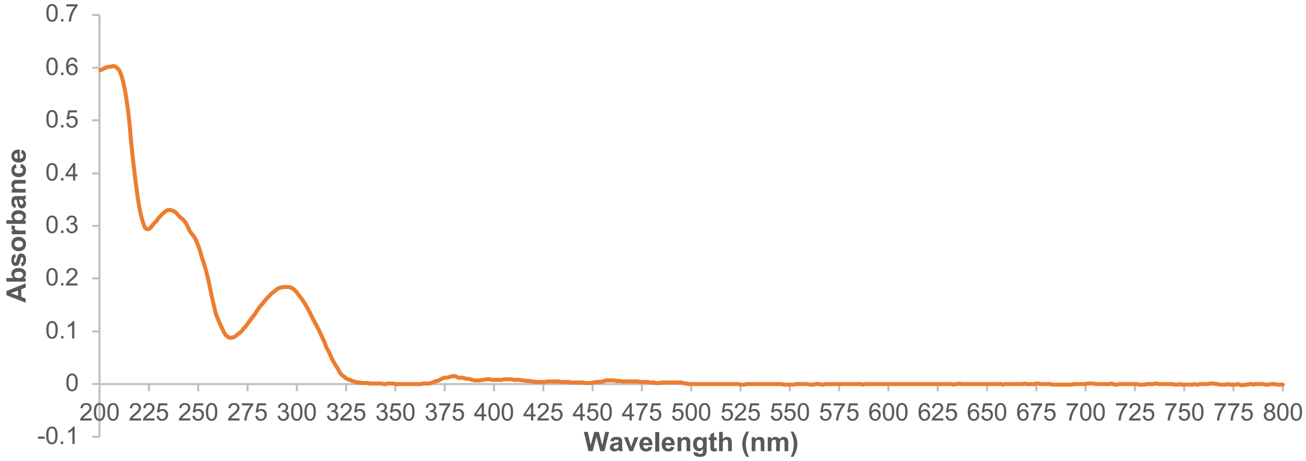

Figure S6. UV-Vis spectrum of PadCTP-AHX-DOTA **1**.

AadUTP-AHX-DOTA **2**

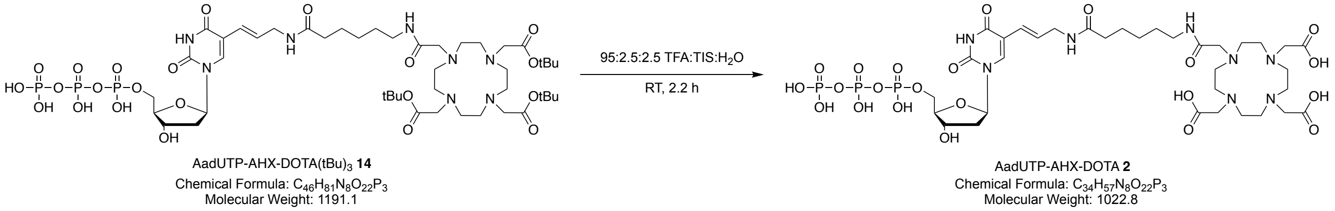

Scheme S14. Synthesis of AadUTP-AHX-DOTA **2**.

AadUTP-AHX-DOTA(tBu)$_3$ (**15**, 1.6 µmol) was the starting material of this reaction, yielding 1.1 µmol product (**2**, 71%). t_R = 8.9 min. ^{31}P NMR (202 MHz, D_2O) δ -11.01 (d, *J* = 18.8 Hz), -11.76 (d, *J* = 22.5 Hz), -23.11 – -23.67 (m). ESI-TOF calcd. $[C_{34}H_{57}N_8O_{22}P_3 + H]^+$ 1023.3, found 1023.3. λ_{max} = 294 nm.

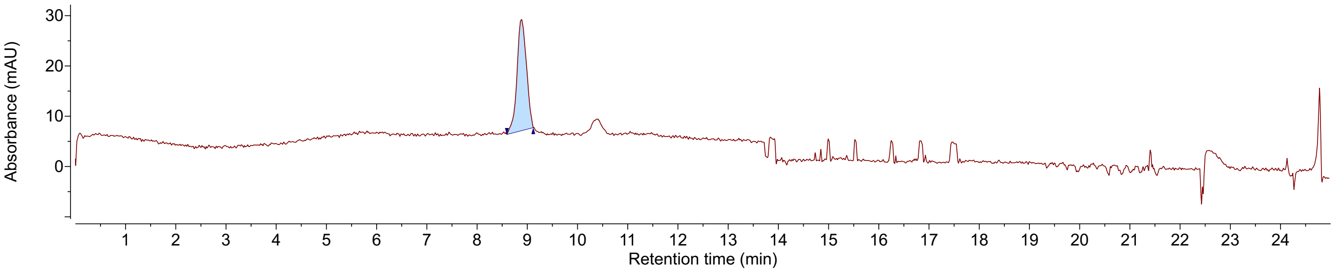

Figure S7. HPLC chromatogram of AadUTP-AHX-DOTA **2**.

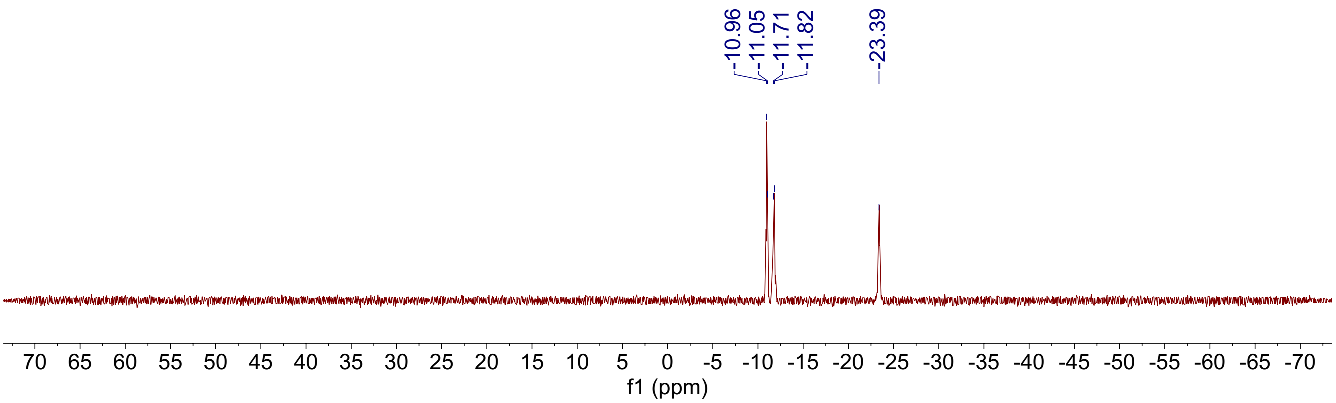

Figure S8. ^{31}P NMR spectrum of AadUTP-AHX-DOTA **2**.

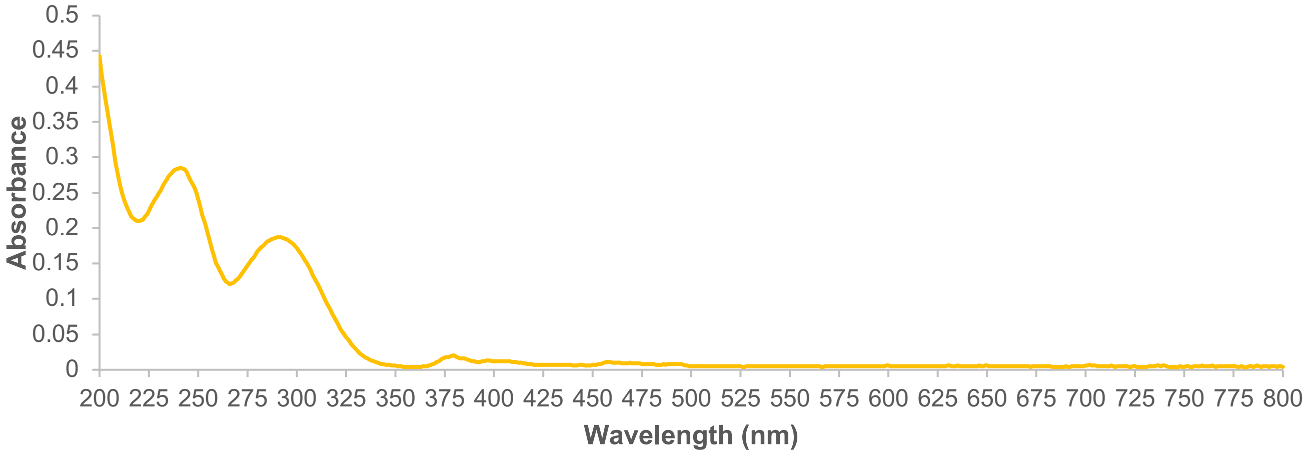

Figure S9. UV-Vis spectrum of AadUTP-AHX-DOTA **2**.

B. Synthesis of XadYTP-PEG$_n$-DOTA series

General acylation steps with DOTA-PEG$_n$-TFP **16–18**

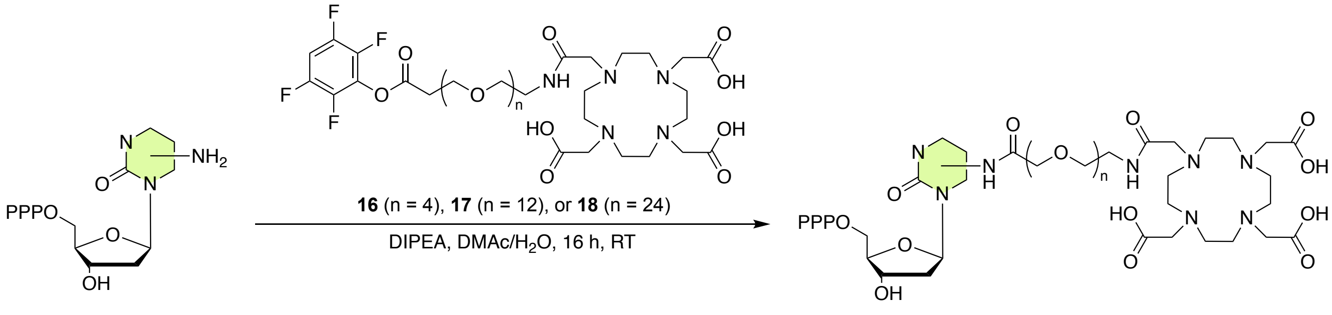

9–10 **19–24**

Scheme S15. General synthetic strategy for PEGylated series **19–24**.

To a 1.5 mL microcentrifuge Tube, an appropriate DOTA-PEG$_n$-TFP (**16** for n = 4, **17** for n = 12, **18** for n = 24, 500 mol, 1.0 eq., 5.0 µL, 100 mM in DMF) was added to either PadCTP (**9**, 500 nmol) or AadUTP (**10**, 500 nmol), followed by DIPEA (1.0 µL). If a white precipitate was formed, ddH_2O (5.0 µL) was supplemented to dissolve the solids. If pH ≤ 7, extra DIPEA would be introduced basify the reaction crude until pH reaches 8-9. Following incubation in 30ºC for

16 h, the solvent was removed *in vacuo* and resuspended in ddH_2O (100 µL) for separation in HPLC, where the gradient is specified below. Product containing fraction was lyophilized to dryness, resuspended in ddH_2O for UV-Vis quantification.

PadCTP-PEG$_4$-DOTA ***19***

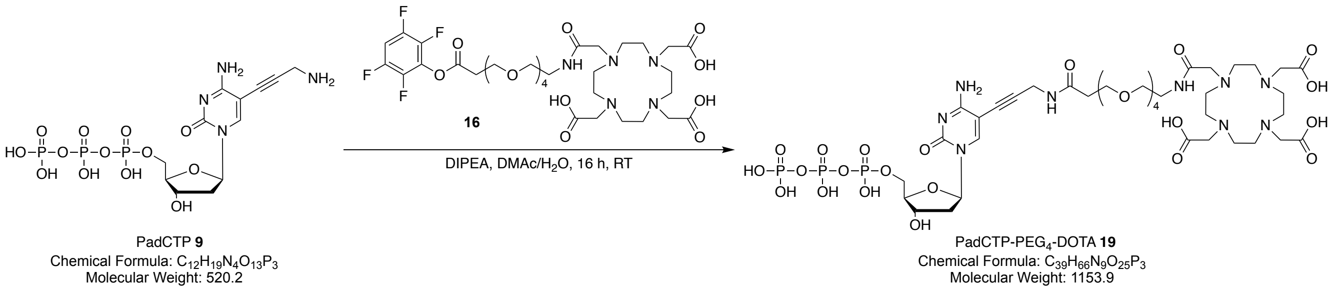

Scheme S16. Synthesis of PadCTP-PEG$_4$-DOTA **19**.

PadCTP (**9**, 500 nmol) and DOTA-PEG$_4$-TFP (**16**, 850 nmol) were the starting materials of this reaction, yielding 252 nmol product (**19**, 50%). t_R = 6.9 min. λ_{max} = 294 nm.

HPLC Method **M-4**: Solvent A, 50 mM triethylammonium bicarbonate (TEAB) in milliQ-filtered H_2O; solvent B, MeCN; 0.0–10.0 min, 0–15% B; 10.0–10.5 min, 15–80% B; 10.5–12.5 min, 80% B; 12.5–13.0 min, 80–0% B; 13.0–15.0 min, 0% B; flow rate, 2.0 mL/min; column temperature, 19ºC – 21ºC.
HPLC Column: Eclipse XDB-C18 5 µm, 250 × 9.6 mm.

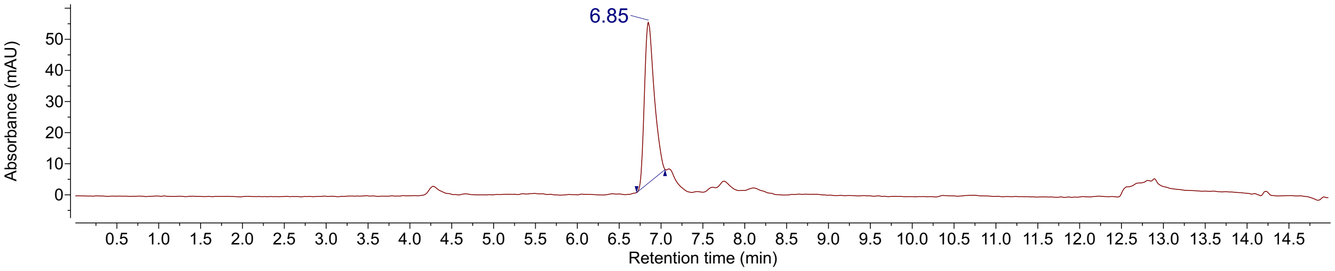

Figure S10. HPLC chromatogram of PadCTP-PEG$_4$-DOTA **19**.

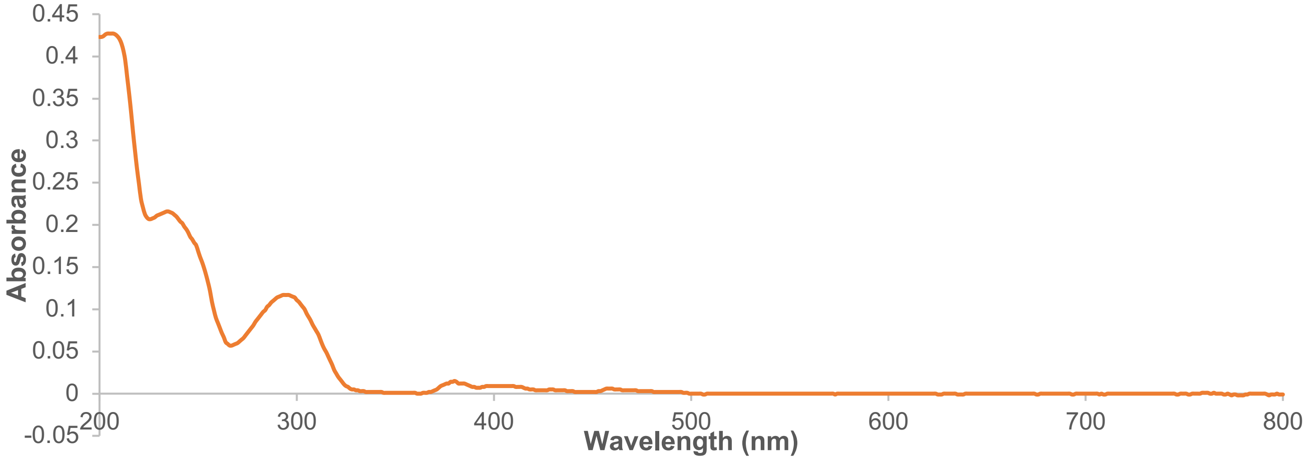

Figure S11. UV-Vis spectrum of PadCTP-PEG$_4$-DOTA **19**.

PadCTP-PEG$_{12}$-DOTA ***20***

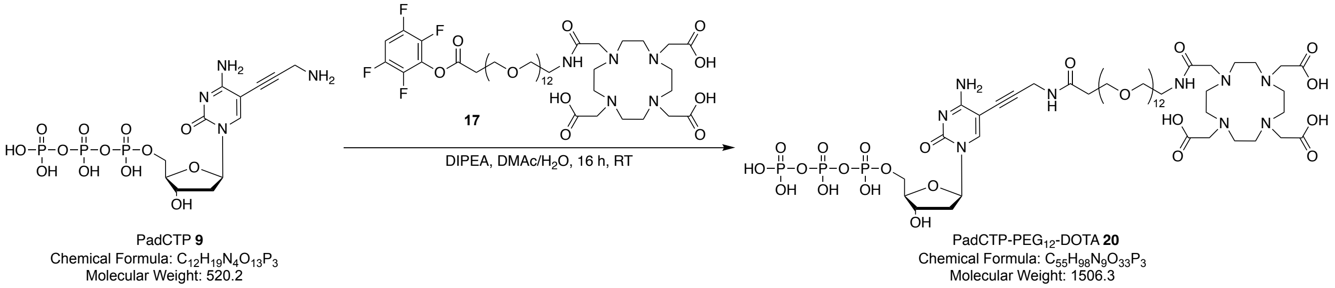

Scheme S17. Synthesis of PadCTP-PEG$_{12}$-DOTA **20**.

PadCTP (**9**, 500 nmol) and DOTA-PEG$_{12}$-TFP (**17**, 850 nmol) were the starting materials of this reaction, yielding 323 nmol product (**20**, 65%). t_R = 4.2 min. λ_{max} = 294 nm.

HPLC Method **M-5**: Solvent A, 50 mM triethylammonium bicarbonate (TEAB) in milliQ-filtered H_2O; solvent B, MeCN; 0.0–10.0 min, 0–80% B; 10.0–10.5 min, 80–80% B; 10.5–12.5 min, 80% B; 12.5–13.0 min, 80–0% B; 13.0–15.0 min, 0% B; flow rate, 2.0 mL/min; column temperature, 19ºC – 21ºC.
HPLC Column: Eclipse XDB-C18 5 µm, 250 × 9.6 mm.

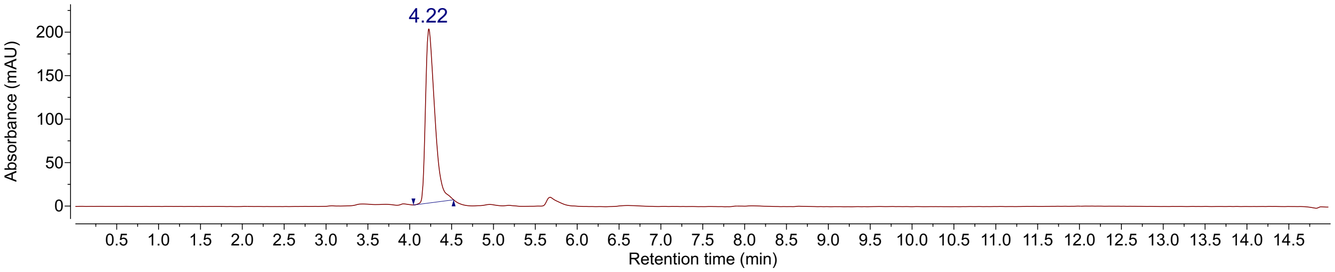

Figure S12. HPLC chromatogram of PadCTP-PEG$_{12}$-DOTA **20**.

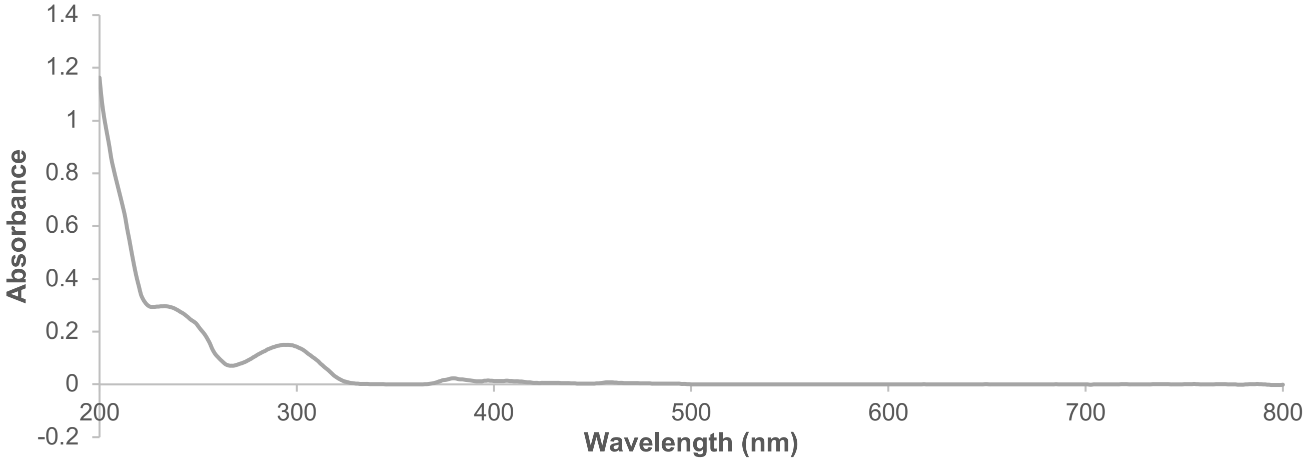

Figure S13. UV-Vis spectrum of PadCTP-PEG$_{12}$-DOTA **20**.

PadCTP-PEG$_{24}$-DOTA ***21***

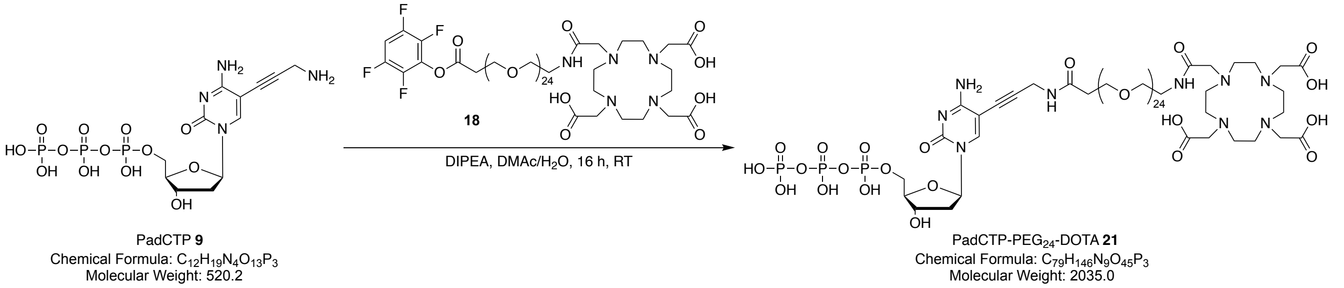

Scheme S18. Synthesis of PadCTP-PEG$_{24}$-DOTA **21**.

PadCTP (**9**, 500 nmol) and DOTA-PEG$_{24}$-TFP (**18**, 850 nmol) were the starting materials of this reaction, yielding 329 nmol product (**21**, 66%). t_R = 4.9 min. λ_{max} = 294 nm.

HPLC Method **M-5**: Solvent A, 50 mM triethylammonium bicarbonate (TEAB) in milliQ-filtered H_2O; solvent B, MeCN; 0.0–10.0 min, 0–80% B; 10.0–10.5 min, 80–80% B; 10.5–12.5 min, 80% B; 12.5–13.0 min, 80–0% B; 13.0–15.0 min, 0% B; flow rate, 2.0 mL/min; column temperature, 19ºC – 21ºC.
HPLC Column: Eclipse XDB-C18 5 µm, 250 × 9.6 mm.

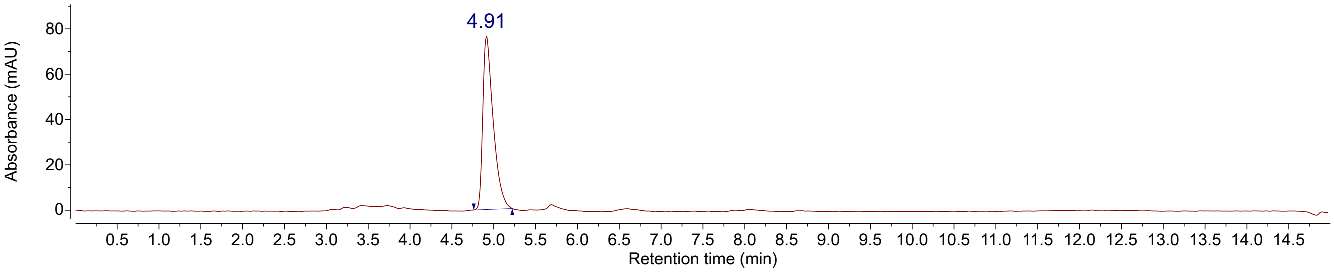

Figure S14. HPLC chromatogram of PadCTP-PEG$_{24}$-DOTA **21**.

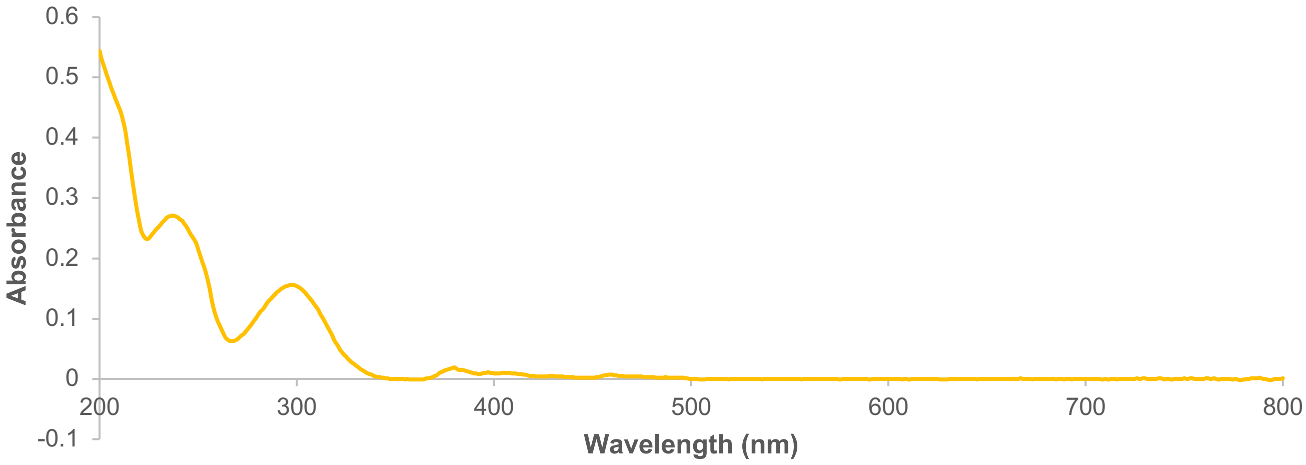

Figure S15. UV-Vis spectrum of PadCTP-PEG$_{24}$-DOTA **21**.

AadUTP-PEG$_4$-DOTA **22**

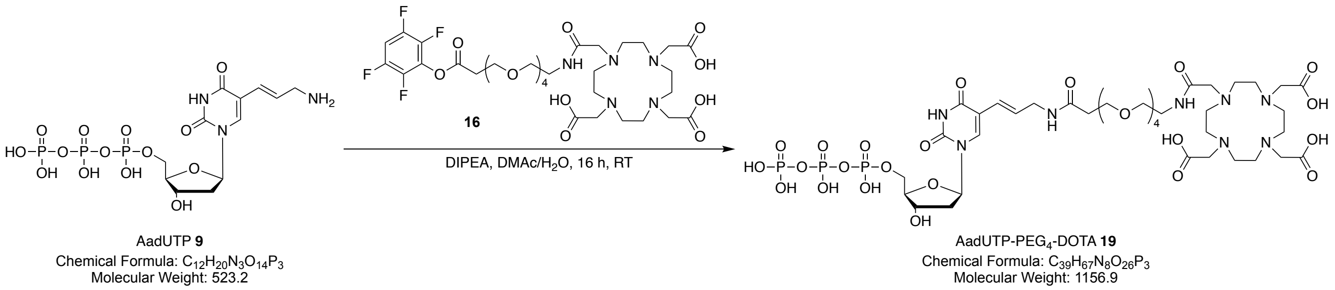

Scheme S19. Synthesis of AadUTP-PEG$_4$-DOTA **22**.

AadUTP (**10**, 500 nmol) and DOTA-PEG$_4$-TFP (**16**, 850 nmol) were the starting materials of this reaction, yielding 490 nmol product (**22**, quant.). t_R = 6.6 min. λ_{max} = 289 nm.

HPLC Method **M-4**: Solvent A, 50 mM triethylammonium bicarbonate (TEAB) in milliQ-filtered H_2O; solvent B, MeCN; 0.0–10.0 min, 0–15% B; 10.0–10.5 min, 15–80% B; 10.5–12.5 min, 80% B; 12.5–13.0 min, 80–0% B; 13.0–15.0 min, 0% B; flow rate, 2.0 mL/min; column temperature, 19ºC – 21ºC.
HPLC Column: Eclipse XDB-C18 5 µm, 250 × 9.6 mm.

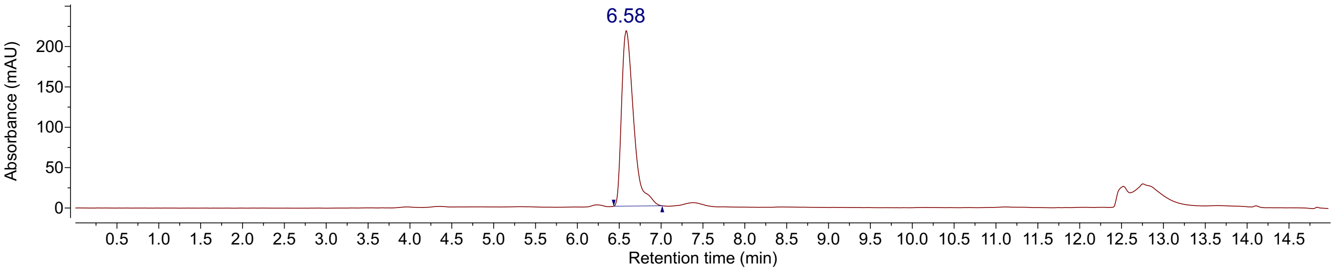

Figure S16. HPLC chromatogram of AadUTP-PEG$_4$-DOTA **22**.

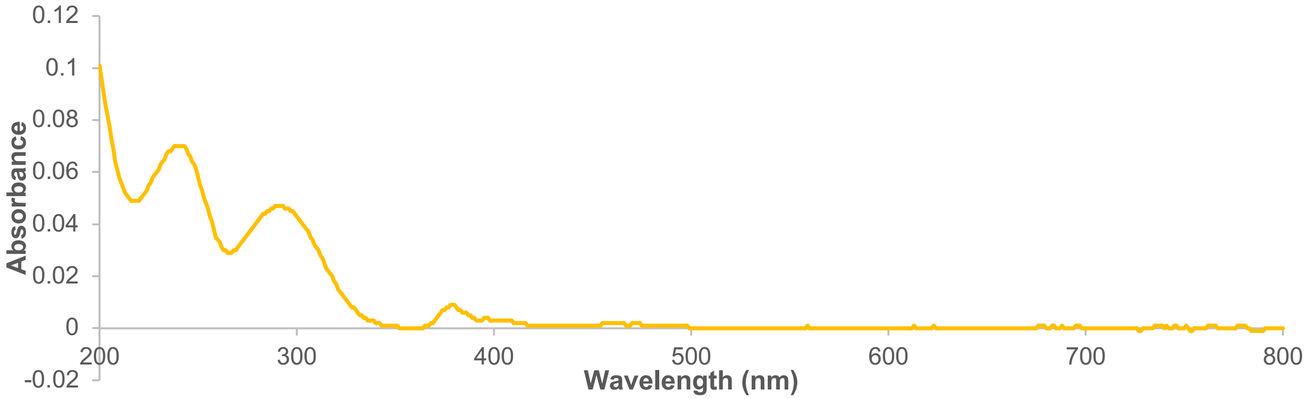

Figure S17. UV-Vis spectrum of AadUTP-PEG$_4$-DOTA **22**.

AadUTP-PEG$_{12}$-DOTA ***23***

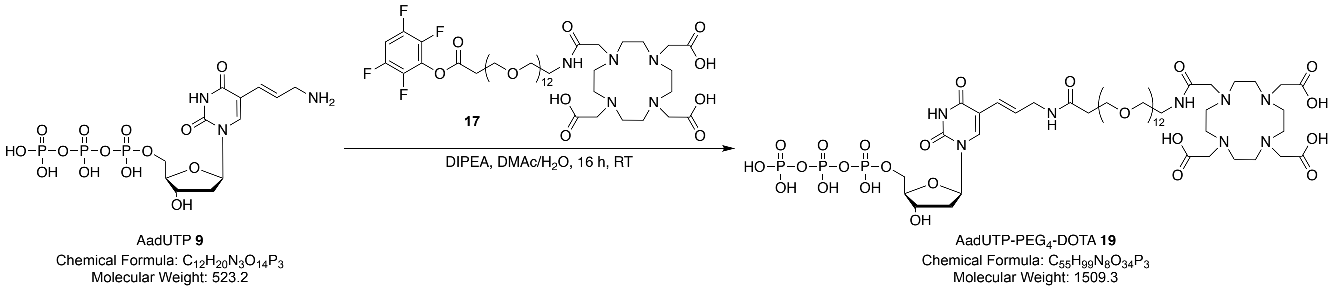

Scheme S20. Synthesis of AadUTP-PEG$_{12}$-DOTA **23**.

AadUTP (**10**, 500 nmol) and DOTA-PEG$_{12}$-TFP (**17**, 850 nmol) were the starting materials of this reaction, yielding 290 nmol product (**23**, 58%). t_R = 4.2 min. λ_{max} = 289 nm.

HPLC Method **M-5**: Solvent A, 50 mM triethylammonium bicarbonate (TEAB) in milliQ-filtered H_2O; solvent B, MeCN; 0.0–10.0 min, 0–80% B; 10.0–10.5 min, 80–80% B; 10.5–12.5 min, 80% B; 12.5–13.0 min, 80–0% B; 13.0–15.0 min, 0% B; flow rate, 2.0 mL/min; column temperature, 19ºC – 21ºC.
HPLC Column: Eclipse XDB-C18 5 µm, 250 × 9.6 mm.

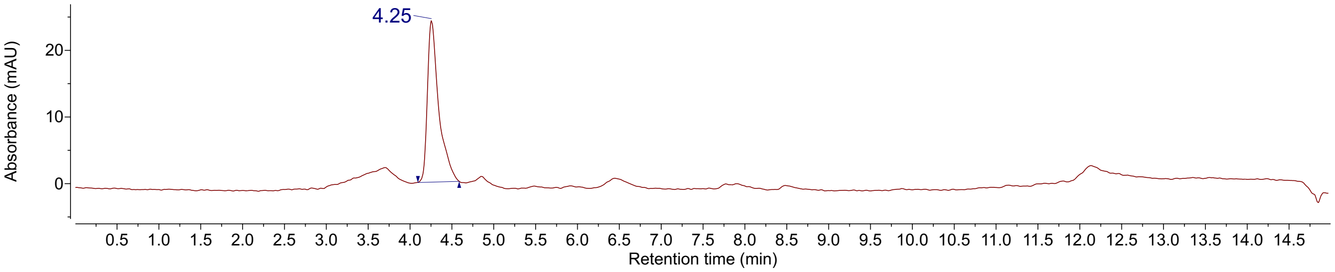

Figure S18. HPLC chromatogram of AadUTP-PEG$_{12}$-DOTA **23**.

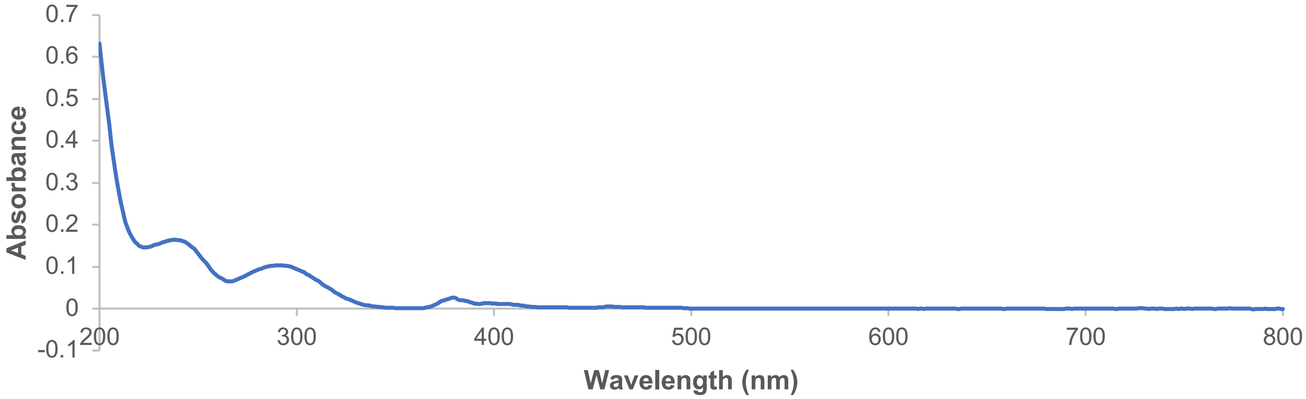

Figure S19. UV-Vis spectrum of AadUTP-PEG$_{12}$-DOTA **23**.

AadUTP-PEG$_{24}$-DOTA ***24***

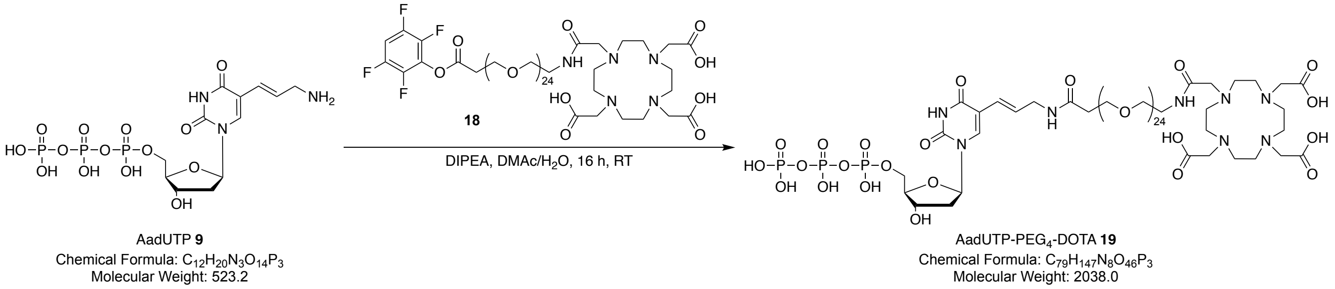

Scheme S21. Synthesis of AadUTP-PEG$_{24}$-DOTA **24**.

AadUTP (**10**, 500 nmol) and DOTA-PEG$_{24}$-TFP (**18**, 850 nmol) were the starting materials of this reaction, yielding 287 nmol product (**24**, 57%). t_R = 4.8 min. λ_{max} = 289 nm.

HPLC Method **M-5**: Solvent A, 50 mM triethylammonium bicarbonate (TEAB) in milliQ-filtered H_2O; solvent B, MeCN; 0.0–10.0 min, 0–80% B; 10.0–10.5 min, 80–80% B; 10.5–12.5 min, 80% B; 12.5–13.0 min, 80–0% B; 13.0–15.0 min, 0% B; flow rate, 2.0 mL/min; column temperature, 19ºC – 21ºC.
HPLC Column: Eclipse XDB-C18 5 µm, 250 × 9.6 mm.

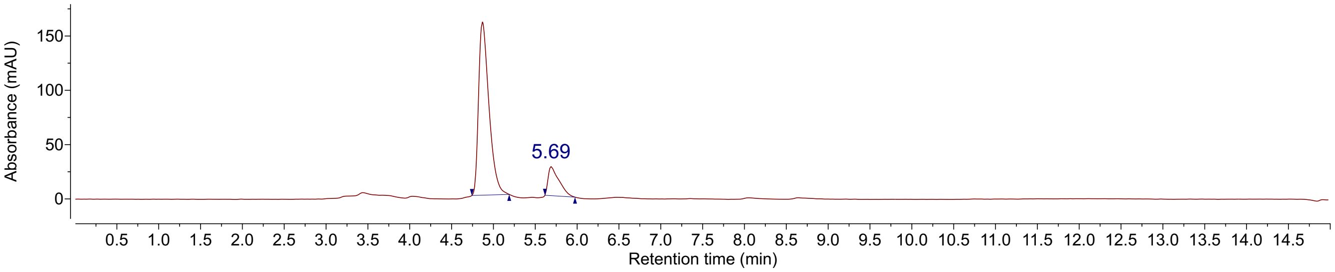

Figure S20. HPLC chromatogram of AadUTP-PEG$_{24}$-DOTA **24**. The minor peak t_R = 5.7 min was 2,3,5,6-tetrafluorophenol, which was inert in further reactions.

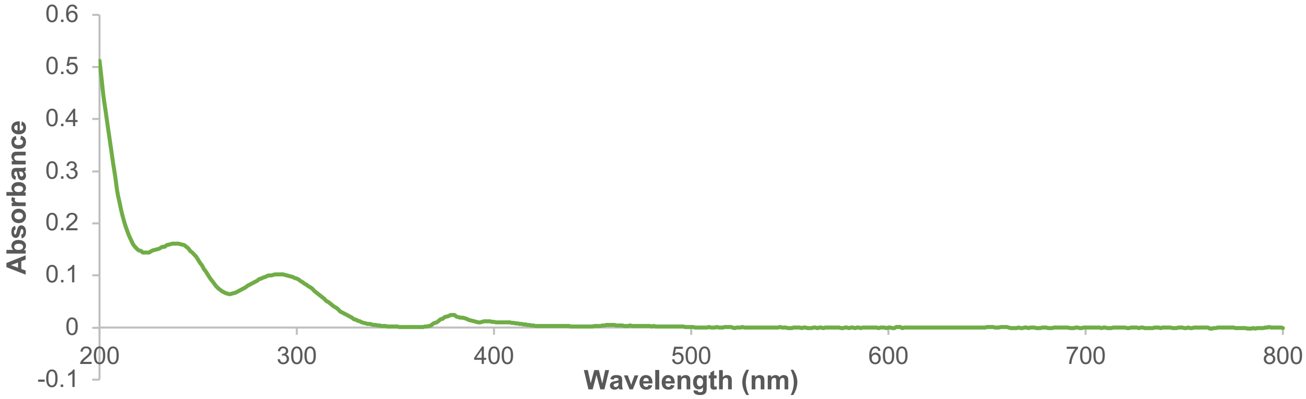

Figure S21. UV-Vis spectrum of AadUTP-PEG$_{24}$-DOTA **24**.

C. Synthesis of XadYTP-Linker-DTPA series

DTPA-Tetrazine **25** and byproduct DTPA-Bis-tetrazine **26**

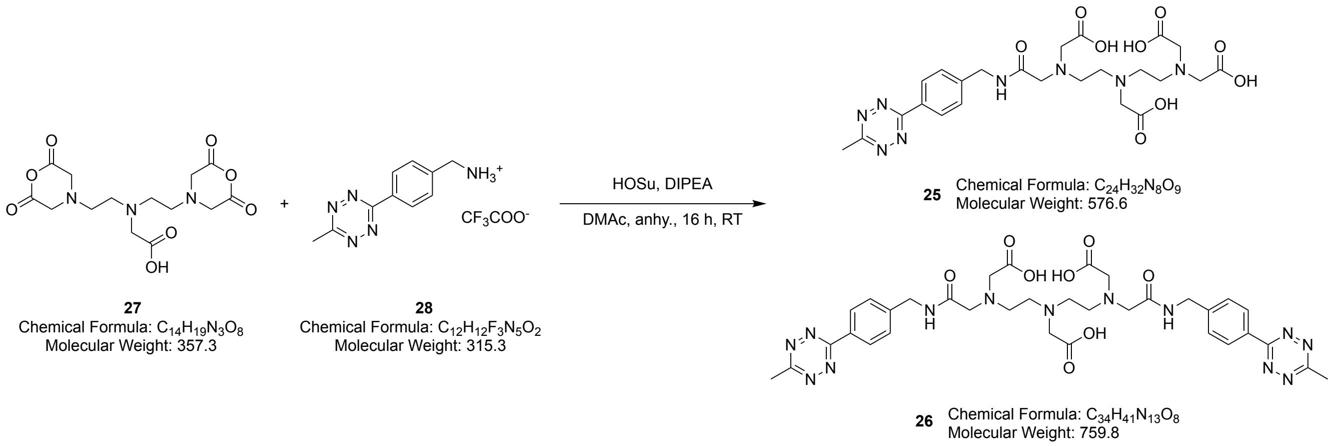

Scheme S22. Synthesis of DTPA-tetrazine **25** and by-product **26**.

To a 15 mL Conical tube with a stir bar, DTPA-dianhydride (**27**, 188 mg, 526 μmol, 11.3 eq.), *N*-hydroxysuccinimide (HOSu, 118 mg, 1.1 mmol, 23.3 eq.) was dissolved in *N*,*N*-dimethylacetamide (4 mL) in 75ºC for 5 minutes to dissolve all reagents to a light yellow solution. Upon cooling to RT, tetrazine-NH_2 trifluoroacetic acid salt (**28**, 20 mg, 500 μL, 93 mM in DMAc, 47 μmol) was added to afford a brightly magenta solution. DIPEA (560 μL, 3.2 mmol, 69 eq.) was added to basify the reaction to pH 8, and reacted in RT for 16 h. The reaction mixture was then concentrated to 0.5 mL solvent and diluted in ddH_2O (60 mL), then loaded onto a pre-activated SepPAK (Waters, WAT036925, 5 g C_{18} sorbent) for purification (20% MeCN). Separation was visibly observable, where the first peak contains the desired product, DTPA-tetrazine (**25**, 24 mg, 47 μmol, 88%) after solvent removal as a magenta oil. ^{1}H NMR (300 MHz, MeOD) δ 8.54 – 8.45 (m, 2H), 7.62 – 7.53 (m, 2H), 4.53 (s, 2H), 3.34 (s, 2H), 3.22 (s, 2H), 3.05 (s, 4H), 3.03 (s, 3H), 3.00 (s, 2H), 2.74 (t, *J* = 5.8 Hz, 2H), 2.58 – 2.47 (m, 6H). ^{13}C NMR (75 MHz, MeOD) δ 179.8, 179.5, 179.4, 174.0, 168.7, 165.3, 145.2, 132.3, 129.5, 129.0, 61.7, 61.1, 60.2, 58.7, 55.5, 55.3, 54.7, 43.6, 21.0. ESI-TOF $[C_{24}H_{32}N_8O_9 + H]^+$ calcd. 577.2, measured 577.4. t_R = 2.3 min. λ_{max} = 268, 520 nm.

The byproduct, DPTA-Bis-tetrazine (**26**), was eluted in later column volumes, and was collected for characterization. ESI-TOF $[C_{34}H_{41}N_{13}O_8 + H]^+$ calcd. 760.3, measured 760.6.

HPLC Method **M-6**: Solvent A, 0.1 % formic acid (% FA) in milliQ-filtered H_2O; solvent B, MeCN with 0.1% FA; 0.0–3.0 min, 0–60% B; 3.0–3.5 min, 60–100% B; 3.5–5.0 min, 100% B; flow rate, 15.0 mL/min; column temperature, 19ºC – 21ºC.
HPLC Column: Agilent Prep 100Å 5 μm, C18, 21.2 × 50 mm.

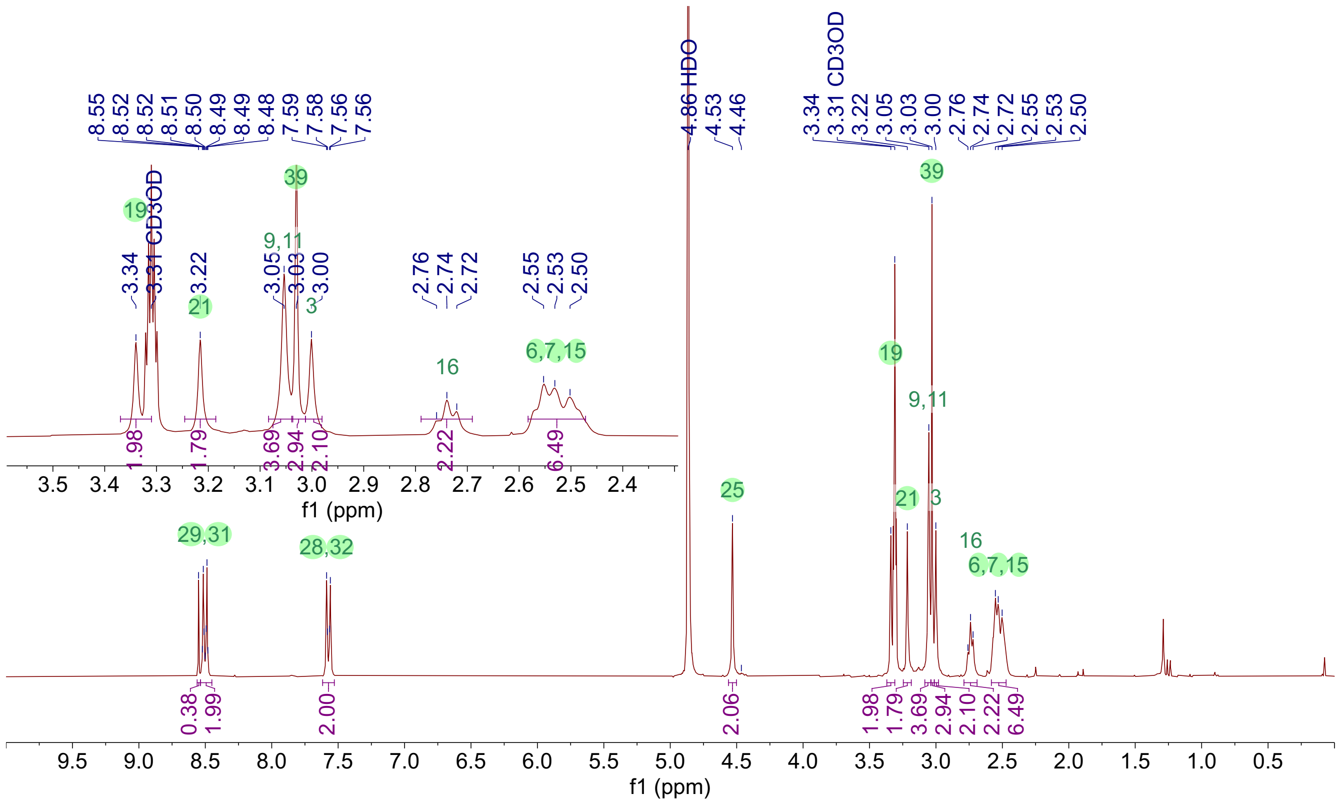

Figure S22. ^{1}H NMR spectrum of DTPA-Tetrazine **25**.

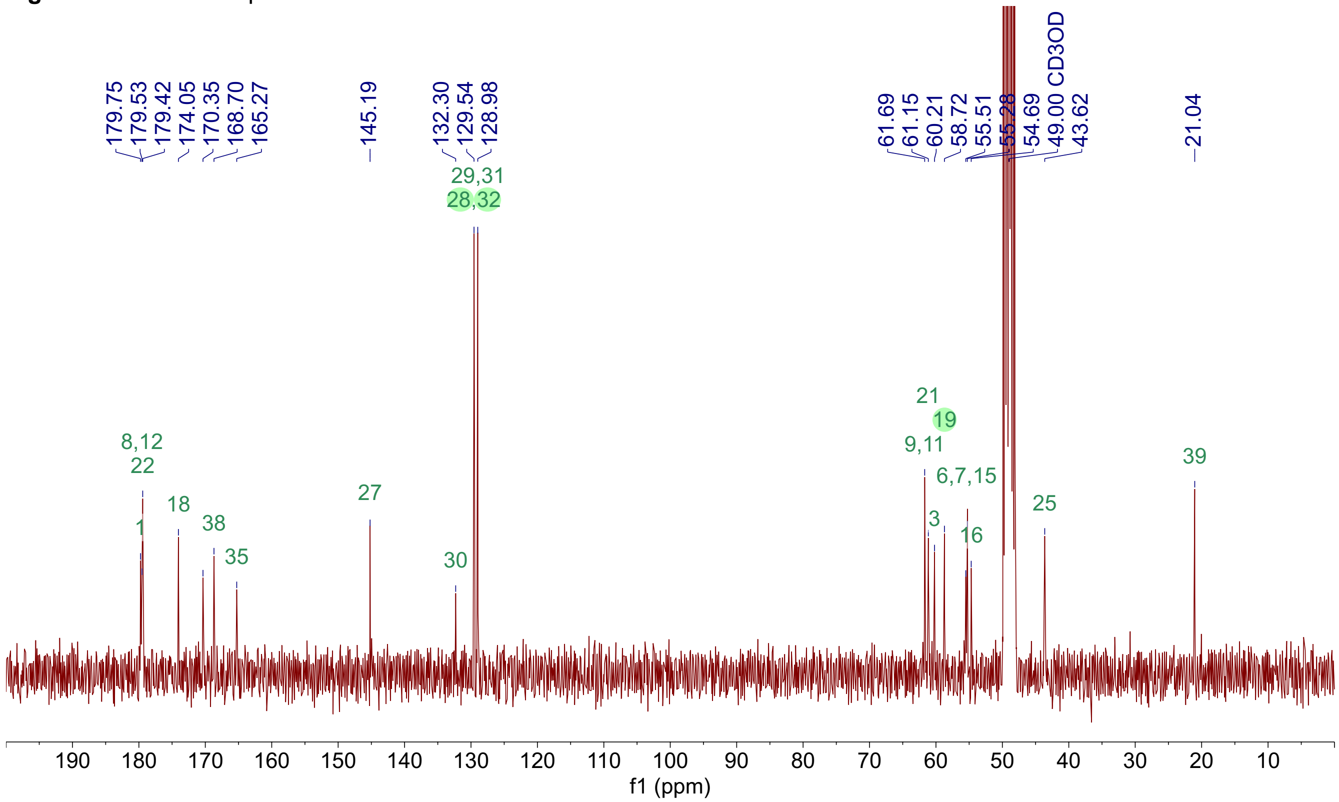

Figure S23. ^{13}C NMR spectrum of DTPA-Tetrazine **25**.

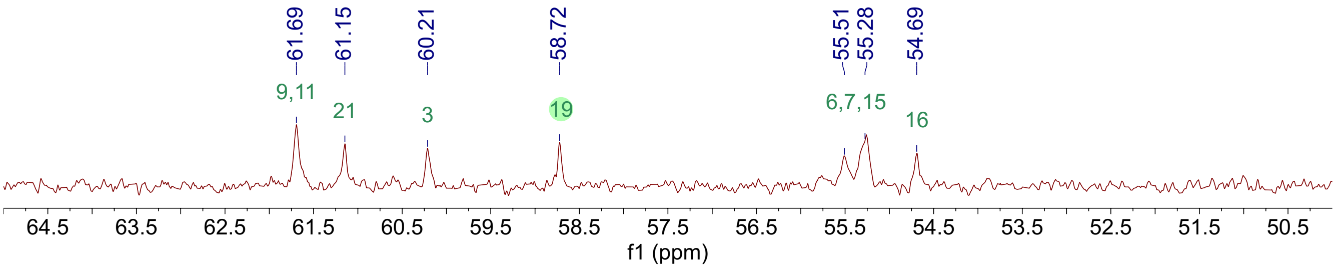

Figure S24. ^{13}C NMR spectrum of DTPA-Tetrazine **25**, zoomed in f_1 50.0–65.0 ppm region.

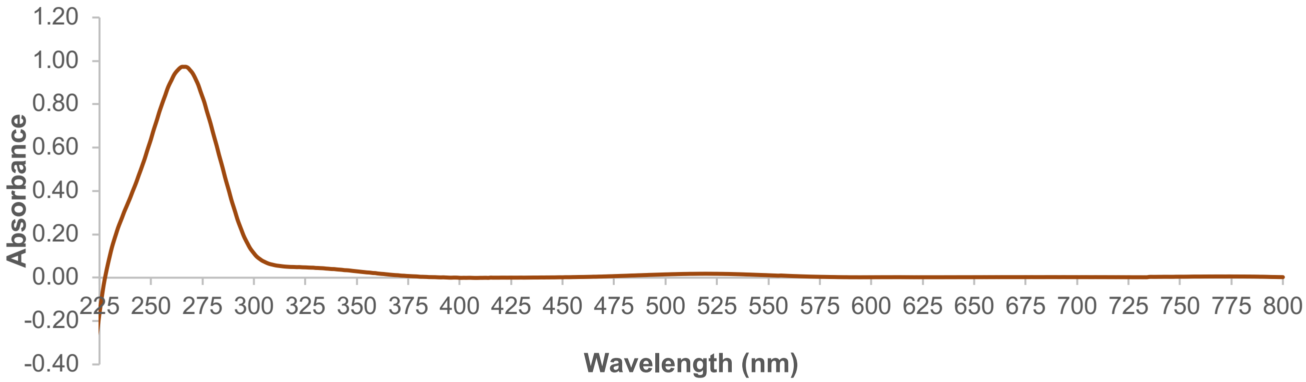

Figure S25. UV-Vis spectrum of DTPA-Tetrazine **25**.

General acylation steps with DTPA-Bis-anhydride **27**

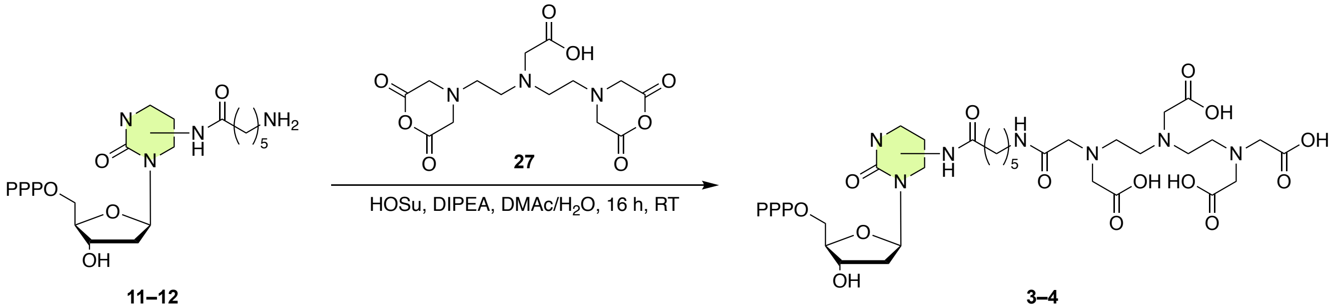

11–12

3–4

Scheme S23. General acylation reaction with DTPA-dianhydride **27**.

To a 1.5 mL microcentrifuge tube, DTPA-dianhydride (**27**, 1.8 mg, 5.0 µmol, 20 eq.), HOSu (0.63 mg, 5.5 µmol, 22 eq.) was dissolved in 18 µL DMAc, and then supplemented with 2.63 µL DIPEA. For sake of accuracy and reproducibility, this recipe was scaled up linearly 5 times in practice, and was aliquoted for preparation of multiple modified nucleotides on the same day. To this aliquot, an appropriate nucleotide triphosphate (either PadCTP-AHX-NH_2 **11** or PadCTP-AHX-NH_2 **12**, 250 nmol, 10 mM in ddH_2O, 25 µL) was added to react in RT for 16 h. The solvent was removed in vacuo, resuspended in ddH_2O (100 µL), and the products **3**, **4** were separated by HPLC.

HPLC Method **M-7**: Solvent A, 50 mM triethylammonium bicarbonate (TEAB) in milliQ-filtered H_2O; solvent B, MeCN; 0.0–20.0 min, 5–20% B; 20.0–20.5 min, 20–100% B; 20.5–22.5 min, 100% B; 22.5–23.0 min, 100–5% B; 23.0–27.0 min, 5% B; flow rate, 1.0 mL/min; column temperature, 19ºC – 21ºC
HPLC Column: Jupiter 10 µm C18 300A, 250 × 4.6 mm.

PadCTP-AHX-DTPA **3**

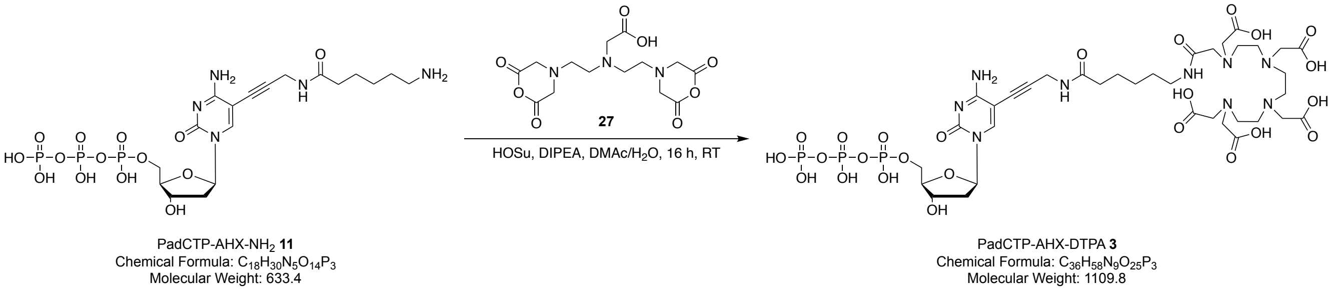

Scheme S24. Synthesis of PadCTP-AHX-DTPA **3**.

PadCTP-AHX-NH_2 (**11**, 250 nmol) was the starting material of this reaction, yielding 207 nmol product (**3**, 83%). t_R = 14.1 min. λ_{max} = 294 nm. ^{31}P NMR (202 MHz, D_2O) δ -9.68 (d), -10.54 (d, *J* = 20.6 Hz), -22.23 (t). ESI-TOF $[C_{32}H_{51}N_8O_{23}P_3 + H]^+$ calcd. 1009.2, measured 1009.2.

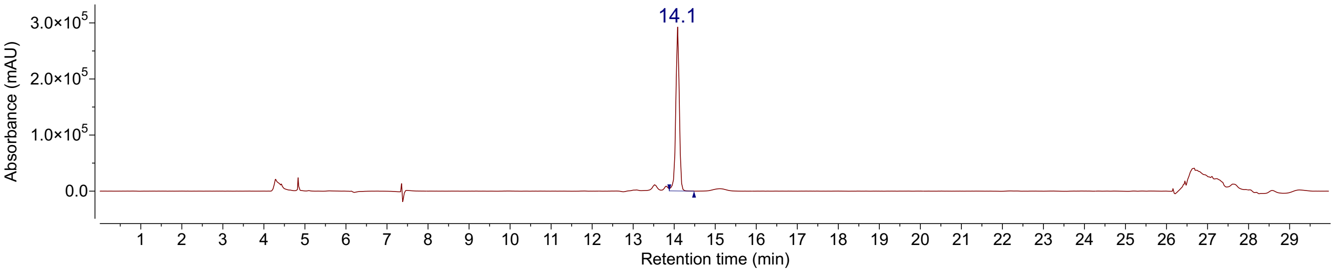

Figure S26. HPLC chromatogram of PadCTP-AHX-DTPA **3**.

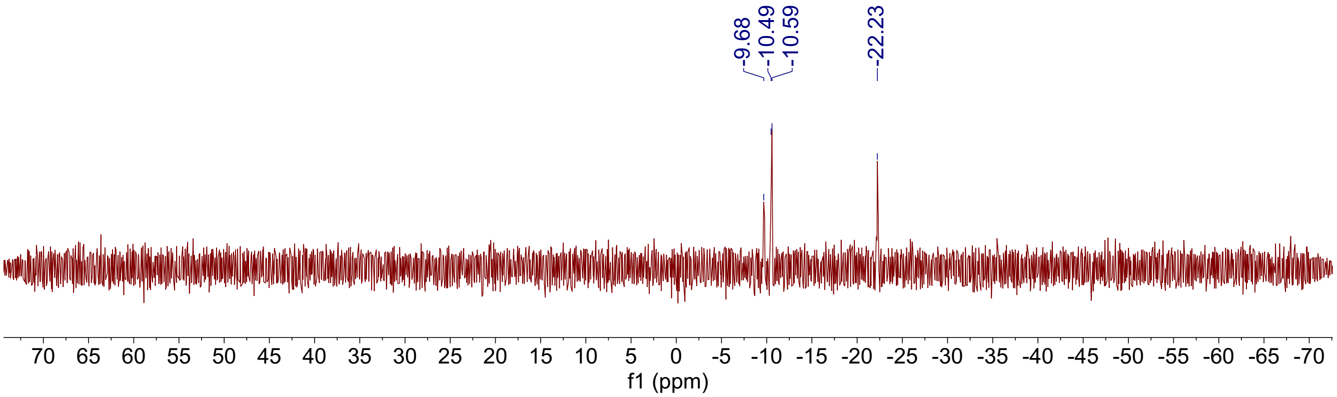

Figure S27. ^{31}P NMR spectrum of PadCTP-AHX-DTPA **3**.

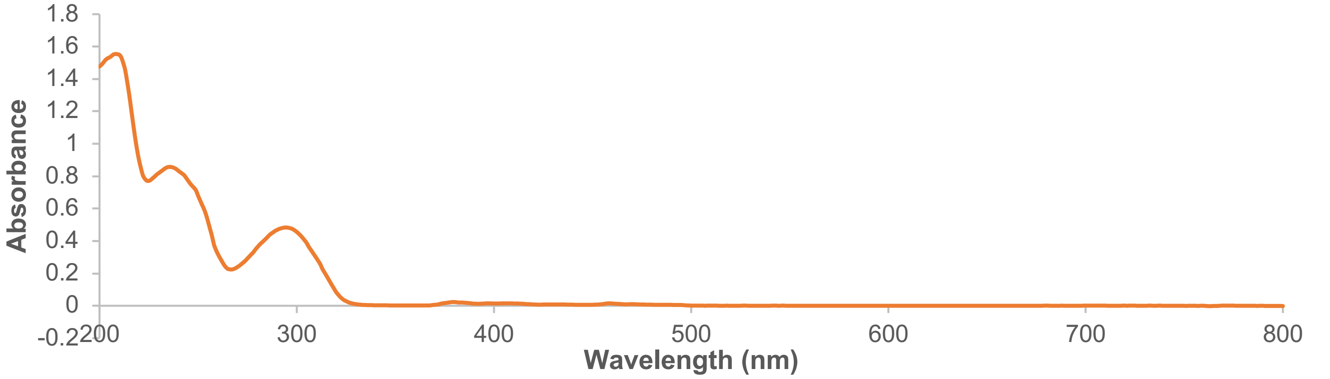

Figure S28. UV-Vis spectrum of PadCTP-AHX-DTPA **3**.

AadUTP-AHX-DTPA **4**

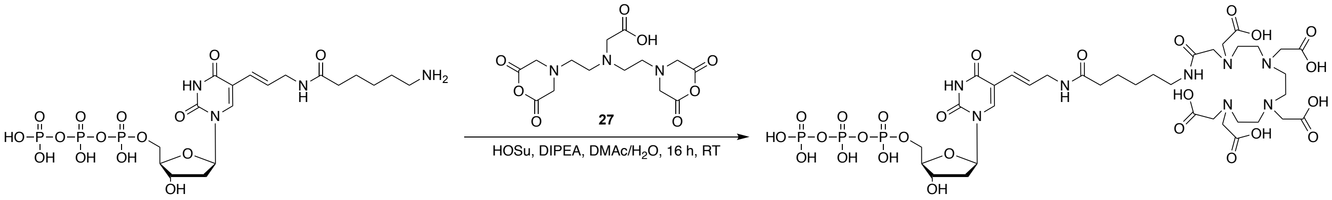

AadUTP-AHX-NH_2 **11**
Chemical Formula: $C_{18}H_{31}N_4O_{15}P_3$
Molecular Weight: 636.4

AadUTP-AHX-DTPA **4**
Chemical Formula: $C_{36}H_{59}N_8O_{26}P_3$
Molecular Weight: 1112.8

Scheme S25. Synthesis of AadUTP-AHX-DTPA **4**.

AadUTP-AHX-NH_2 (**12**, 250 nmol) was the starting material of this reaction, yielding 214 nmol product (**4**, 86%). t_R = 13.6 min. λ_{max} = 289 nm. ^{31}P NMR (202 MHz, D_2O) δ -10.48 (d), -11.52 (d), -23.01 (t). ESI-TOF $[C_{32}H_{52}N_7O_{24}P_3 + H]^+$ calcd. 1012.2, measured 1012.3.

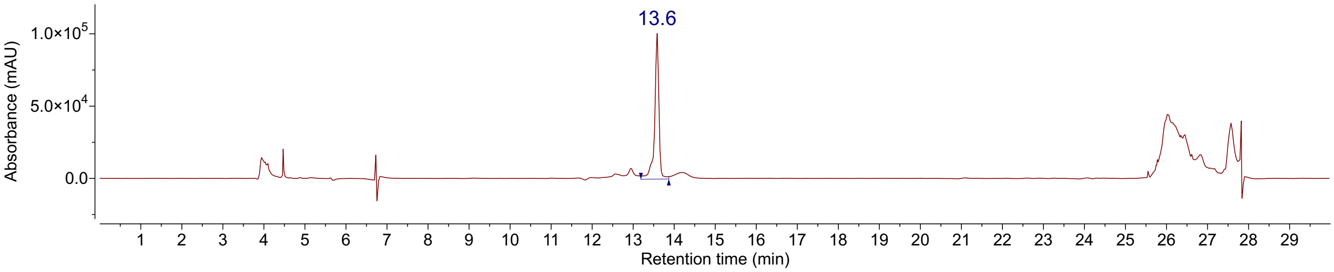

Figure S29. HPLC chromatogram of AadUTP-AHX-DTPA **4**.

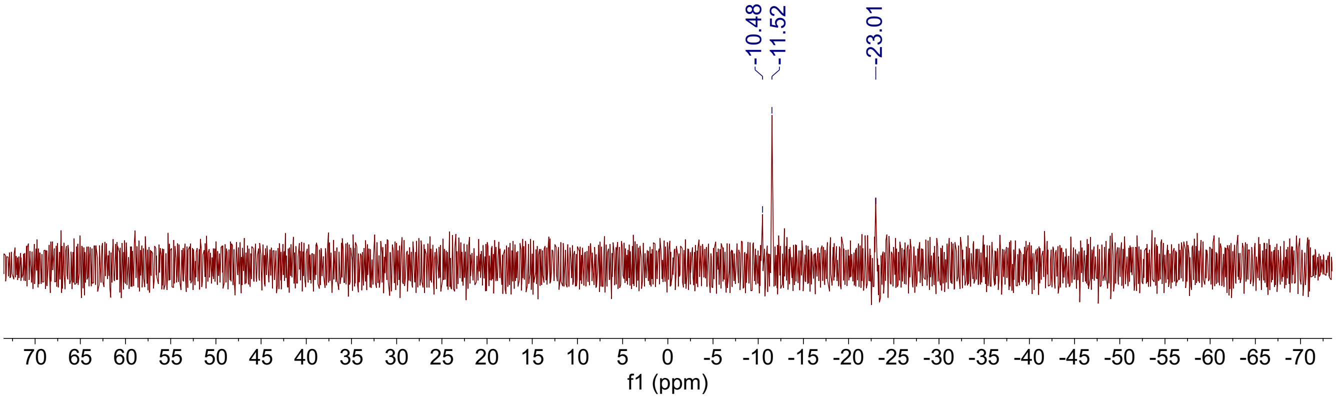

Figure S30. ^{31}P NMR spectrum of AadUTP-AHX-DTPA **4**.

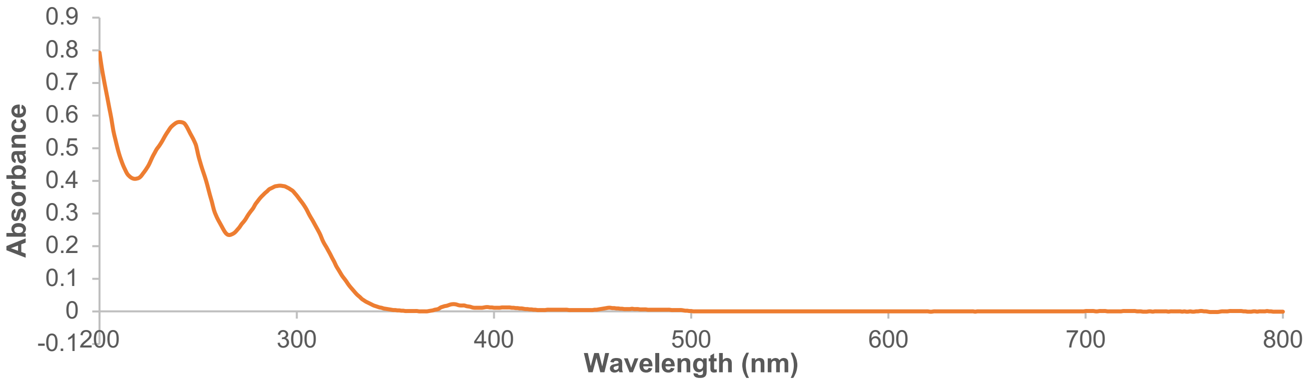

Figure S31. UV-Vis spectrum of AadUTP-AHX-DTPA **4**.

General acylation steps with TCO-PEG$_4$-OSu **29** and TCO-PEG$_{12}$-OSu **30**

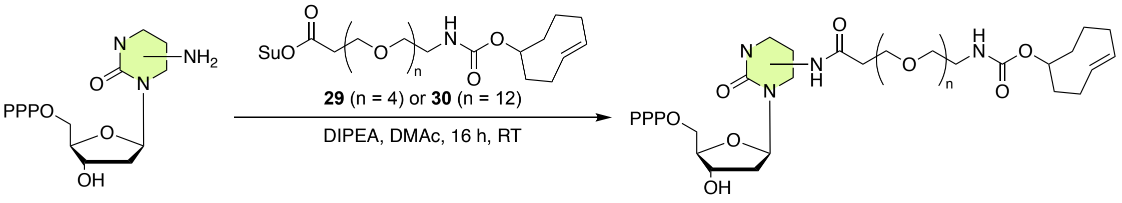

9–10 **31–34**

Scheme S26. General acylation reaction with TCO-PEG$_4$-OSu **29** and TCO-PEG$_{12}$-OSu **30.**

To a 1.5 mL microcentrifuge tube, the appropriate TCO-PEG$_n$-OSu (**29** for n = 4, **30** for n = 12, 519 mM in DMAc, 16.4 µL, 8.5 µmol, 1.7 eq.) was added to a solution of nucleotide triphosphate (**9** or **10**, 100 mM in ddH$_2$O, 50 µL, 5 µmol), and then supplemented with DIPEA (2 µL). If pH ≤ 7, extra DIPEA was added to basify the reaction mixture until pH 8-9 was reached. After 16 µL, the solvent was removed *in vacuo*, resuspended in ddH$_2$O, and separated by HPLC.

HPLC Method **M-8**: Solvent A, 50 mM triethylammonium bicarbonate (TEAB) in milliQ-filtered H$_2$O; solvent B, MeCN; 0.0–10.0 min, 5–75% B; 10.0–10.5 min, 75–100% B; 10.5–12.5 min, 100% B; 12.5–13.0 min, 100–5% B; 13.0–16.0 min, 5% B; flow rate, 2.0 mL/min; column temperature, 19ºC – 21ºC
HPLC Column: Jupiter 10 µm C18 300A, 250 × 4.6 mm

*PadCTP-PEG$_4$-TCO **31***

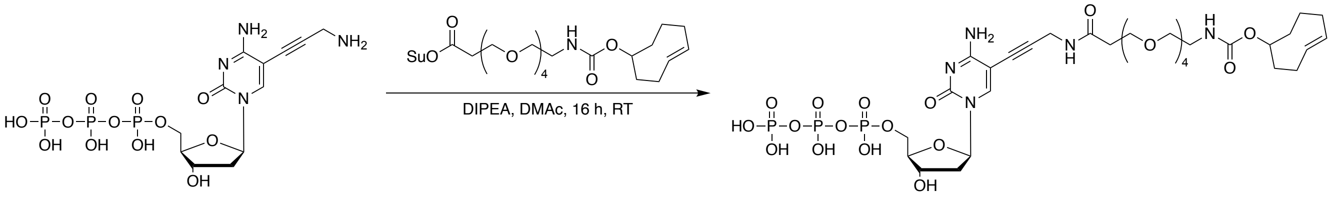

PadCTP **9**
Chemical Formula: $C_{12}H_{19}N_4O_{13}P_3$
Molecular Weight: 520.2

PadCTP-PEG$_4$-TCO **31**
Chemical Formula: $C_{32}H_{52}N_5O_{20}P_3$
Molecular Weight: 919.7

Scheme S27. Synthesis of PadCTP-PEG$_4$-TCO **31**.

PadCTP (**9**, 5.0 µmol) was the starting material of this reaction, yielding 1.6 µmol product (**31**, 32%). t_R = 6.5 min. λ_{max} = 294 nm.

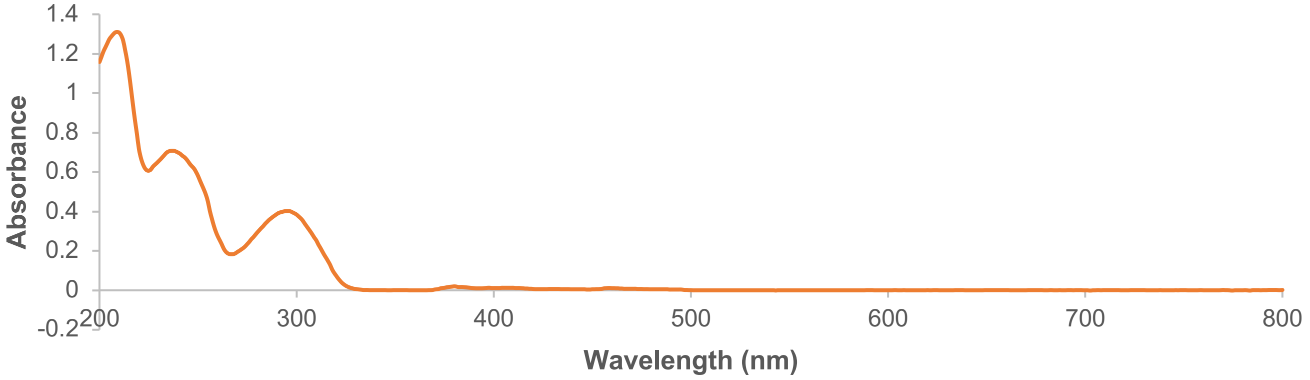

Figure S32. UV-Vis spectrum of PadCTP-PEG$_4$-TCO **31**.

PadCTP-PEG$_{12}$-TCO ***32***

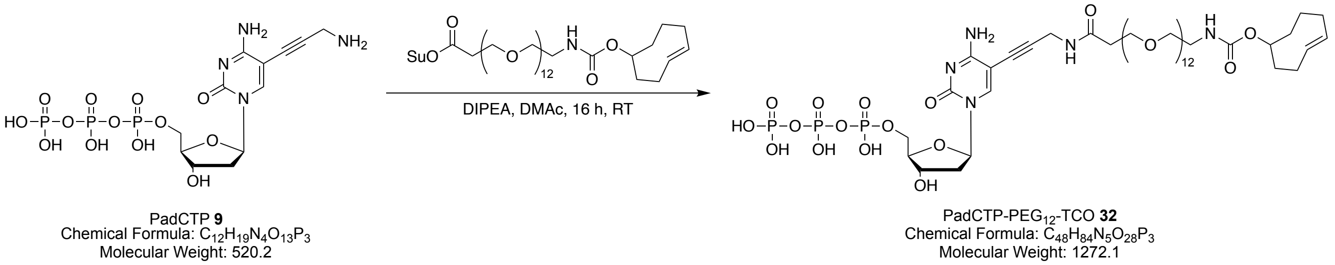

Scheme S28. Synthesis of PadCTP-PEG$_{12}$-TCO **32**.

PadCTP (**9**, 5.0 µmol) was the starting material of this reaction, yielding 1.5 µmol product (**32**, 31%). t_R = 7.2 min. λ_{max} = 294 nm.

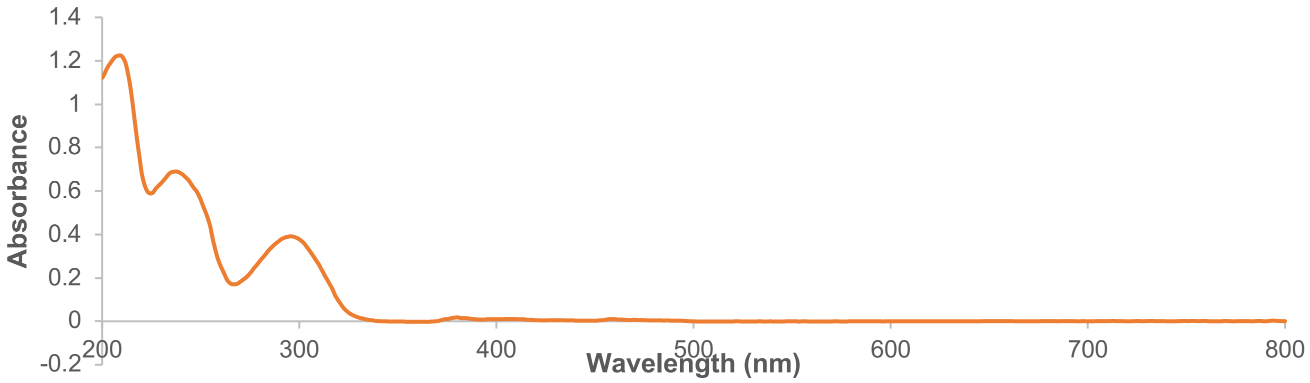

Figure S33. UV-Vis spectrum of PadCTP-PEG$_{12}$-TCO **32**.

AadUTP-PEG$_4$-TCO ***33***

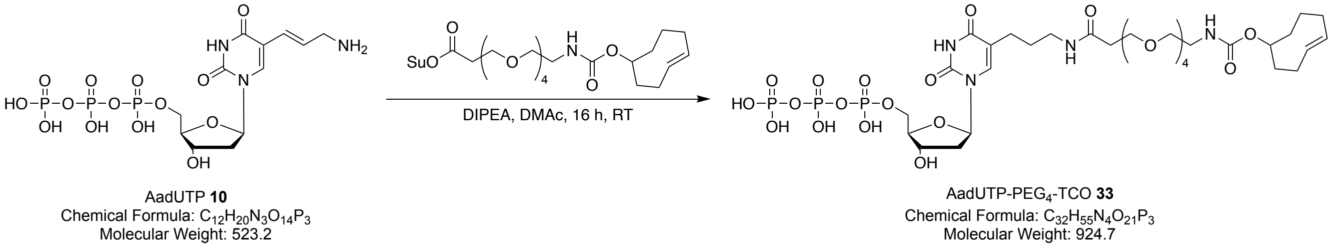

Scheme S29. Synthesis of AadUTP-PEG$_4$-TCO **33**.

AadUTP (**10**, 5.0 µmol) was the starting material of this reaction, yielding 496 nmol product (**33**, 10%). t_R = 6.6 min. λ_{max} = 289 nm.

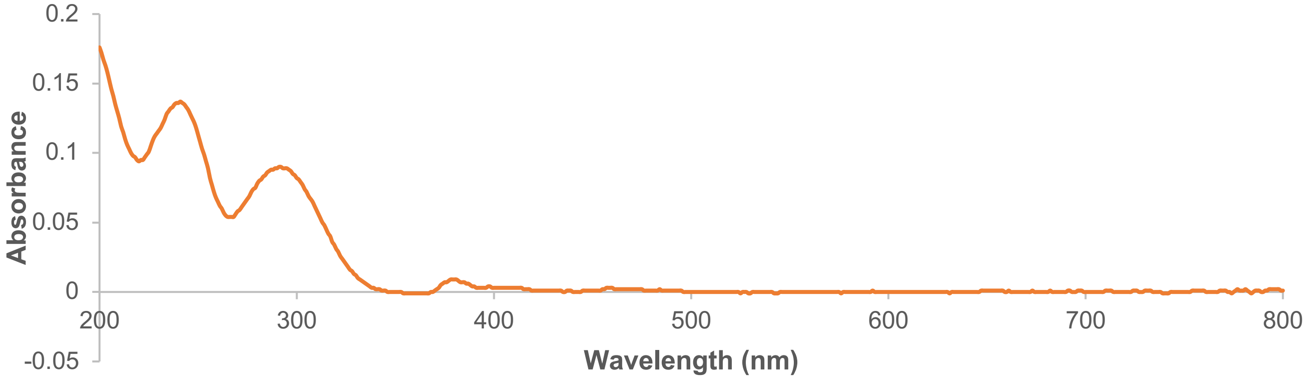

Figure S34. UV-Vis spectrum of AadUTP-PEG$_4$-TCO **33**.

AadUTP-PEG$_{12}$-TCO ***34***

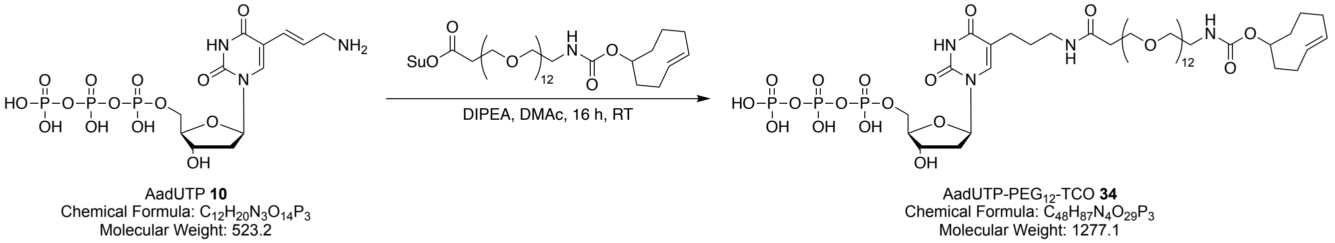

Scheme S30. Synthesis of AadUTP-PEG$_{12}$-TCO **34**.

AadUTP (**10**, 5.0 µmol) was the starting material of this reaction, yielding 766 nmol product (**34**, 15%). t_R = 7.3 min. λ_{max} = 289 nm.

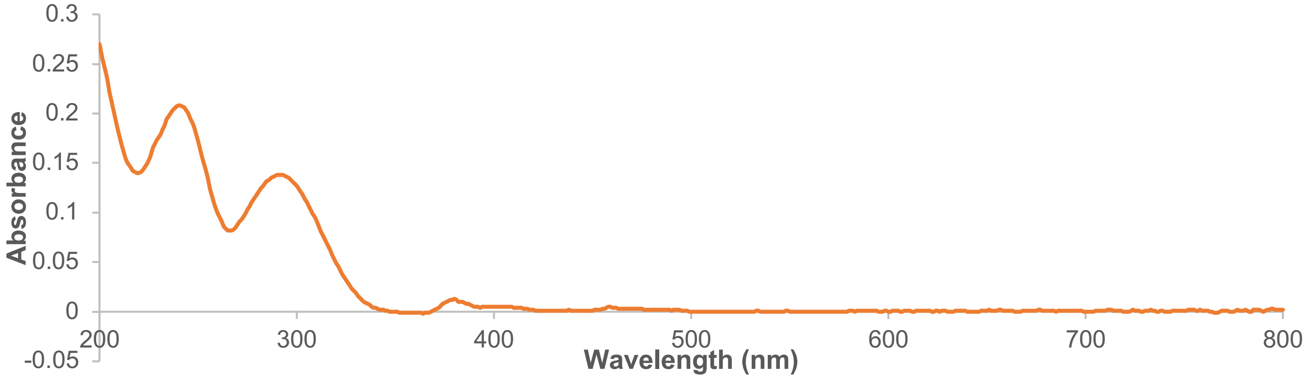

Figure S35. UV-Vis spectrum of AadUTP-PEG$_{12}$-TCO **34**.

General SPAAC steps with DTPA-Tetrazine **25**

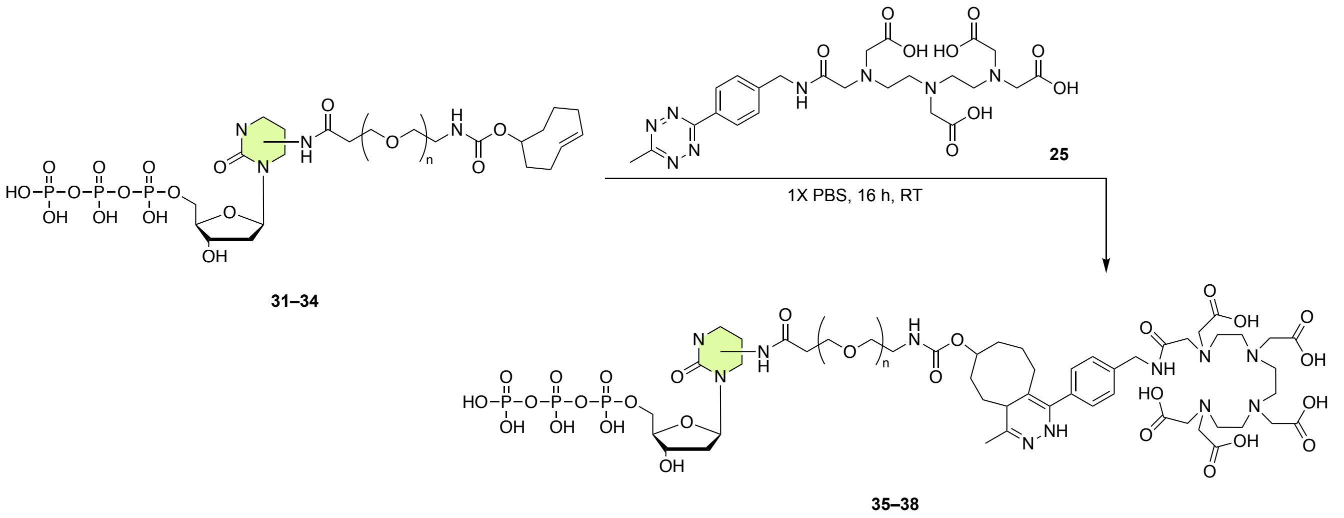

Scheme S31. General strain-promoted alkyne-azide cycloaddition (SPAAC) reaction with DTPA-Tetrazine **25**.

To a 1.5 mL microcentrifuge tube, DTPA-tetrazine (**25**, 14 mM in ddH_2O, 57 µL, 800 nmol) was added to a solution of nucleotide triphosphate (5 mM in ddH_2O, 250–800 nmol). In case of incomplete dissolution, MeCN (up to 50 µL) was added to completely solubilize the mixture. The reaction was allowed to react in RT for 16 h, following solvent removal and analysis by HPLC.

HPLC Method **M-8**: Solvent A, 50 mM triethylammonium bicarbonate (TEAB) in milliQ-filtered H_2O; solvent B, MeCN; 0.0–10.0 min, 5–75% B; 10.0–10.5 min, 75–100% B; 10.5–12.5 min, 100% B; 12.5–13.0 min, 100–5% B; 13.0–16.0 min, 5% B; flow rate, 2.0 mL/min; column temperature, 19ºC – 21ºC
HPLC Column: Jupiter 10 µm C18 300A, 250 × 4.6 mm

PadCTP-PEG$_4$-DTPA **35**

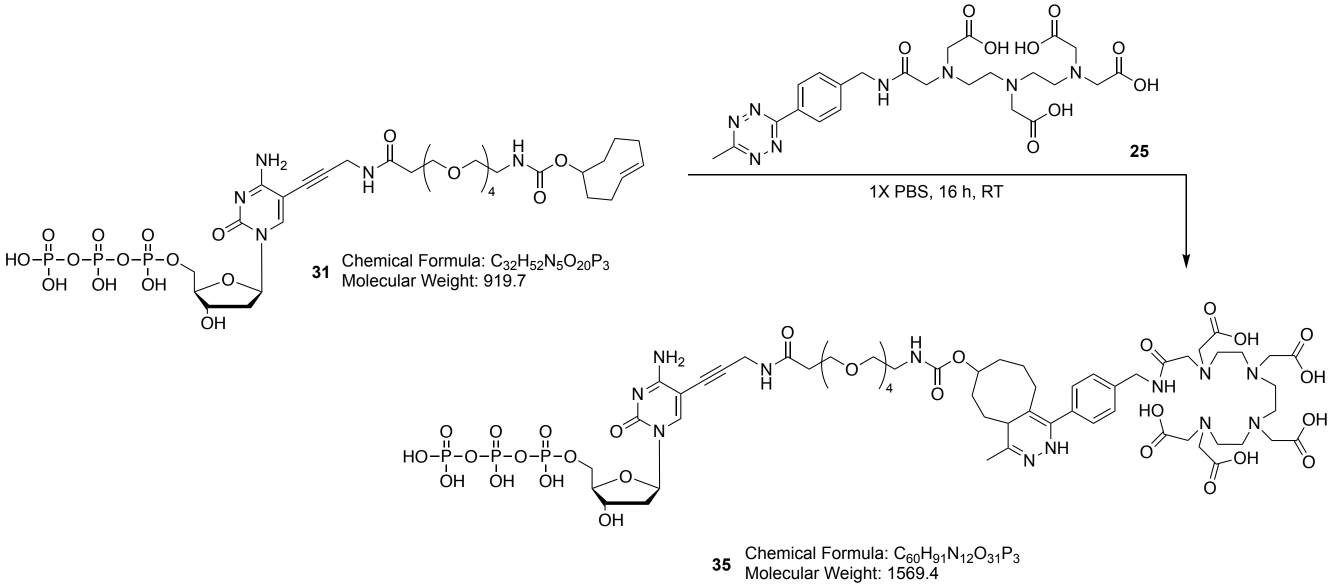

Scheme S32. Synthesis of PadCTP-PEG$_4$-DTPA **35**.

PadCTP-PEG$_4$-TCO (**31**, 800 nmol) was the starting material of this reaction, yielding 293 nmol product (**35**, 37%). t_R = 4.7 min. λ_{max} = 294 nm.

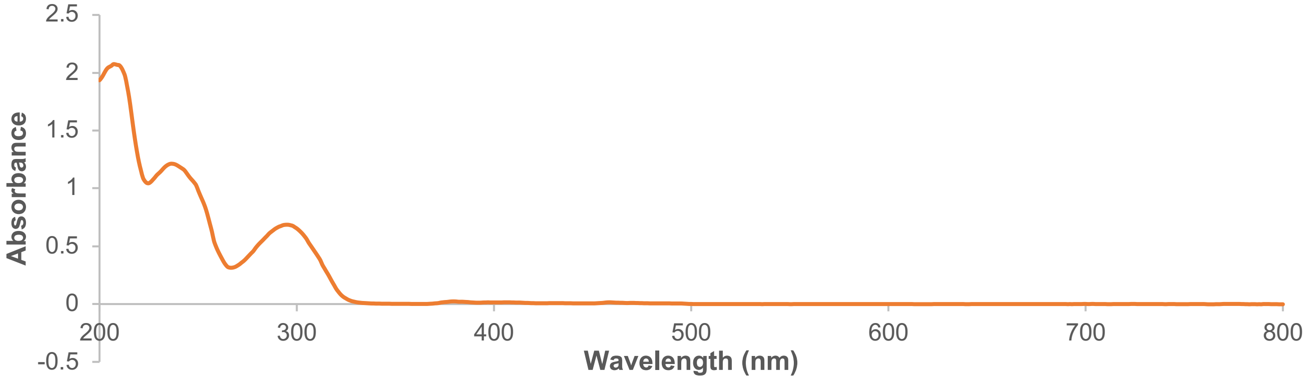

Figure S36. UV-Vis spectrum of PadCTP-PEG$_4$-DTPA **35**.

PadCTP-PEG$_{12}$-DTPA ***36***

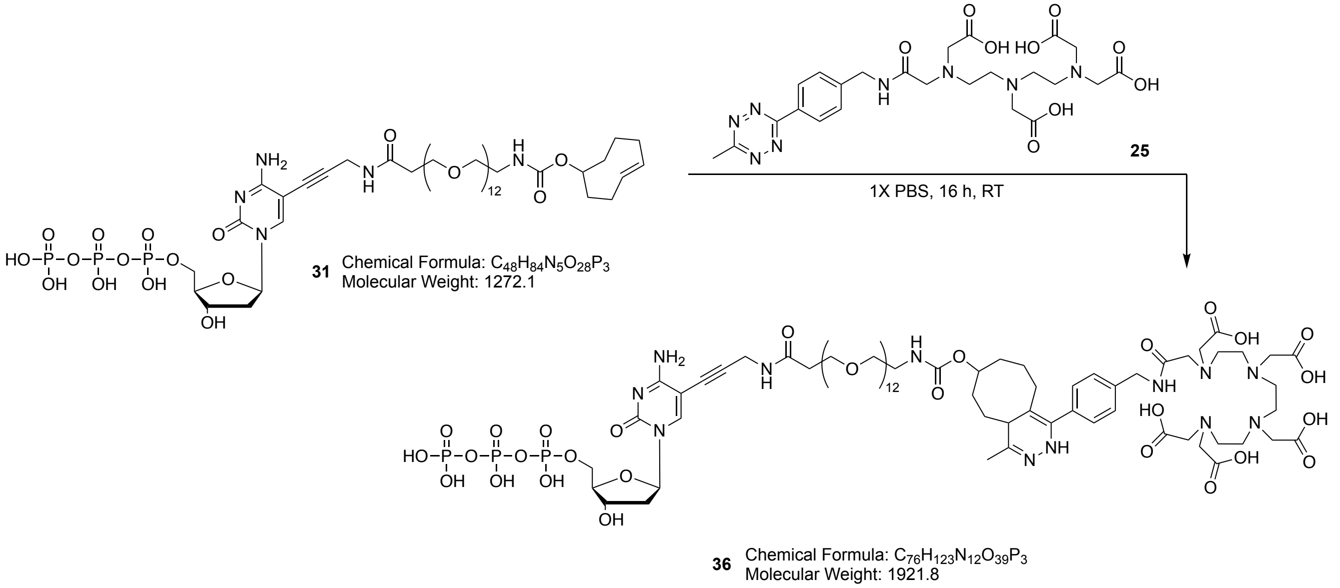

Scheme S33. Synthesis of PadCTP-PEG$_{12}$-DTPA **36**.

PadCTP-PEG$_{12}$-TCO (**32**, 800 nmol) was the starting material of this reaction, yielding 264 nmol product (**36**, 33%). t_R = 4.6 min. λ_{max} = 294 nm.

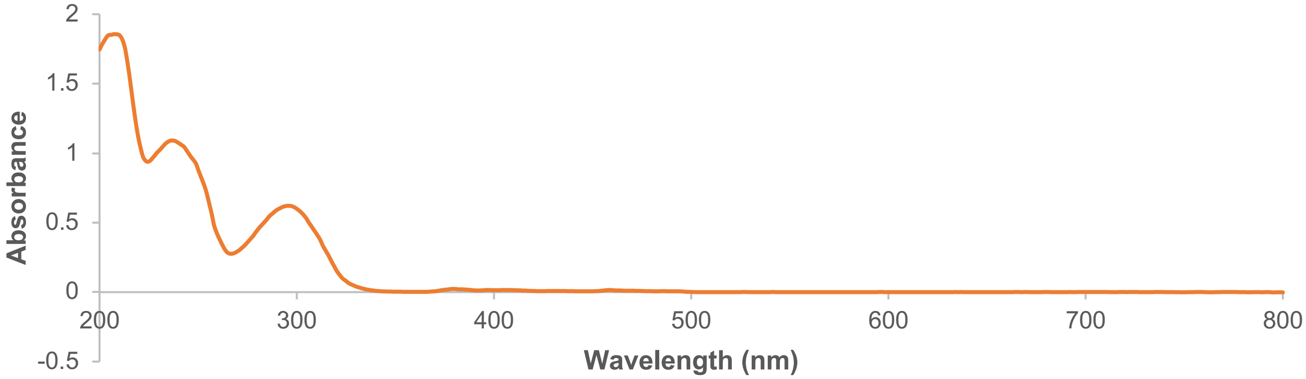

Figure S37. UV-Vis spectrum of PadCTP-PEG$_{12}$-DTPA **36**.

AadUTP-PEG$_4$-DTPA ***37***

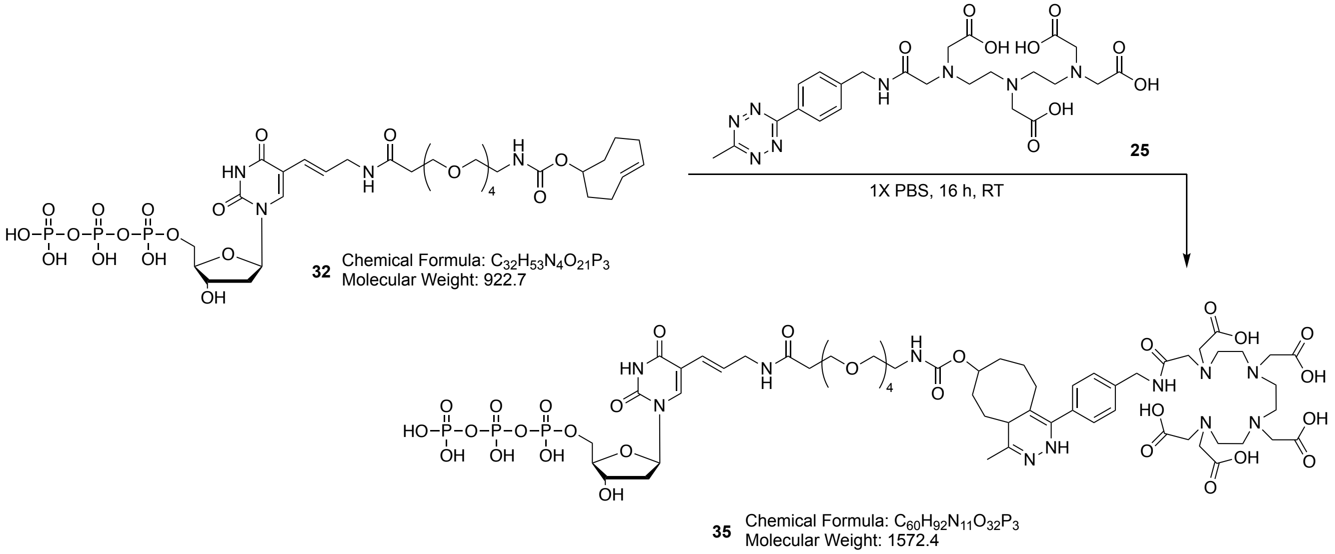

Scheme S34. Synthesis of AadUTP-PEG$_{12}$-DTPA **37**.

AadUTP-PEG$_4$-TCO (**33**, 250 nmol) was the starting material of this reaction, yielding 35 nmol product (**37**, 14%). t_R = 4.7 min. λ_{max} = 289 nm.

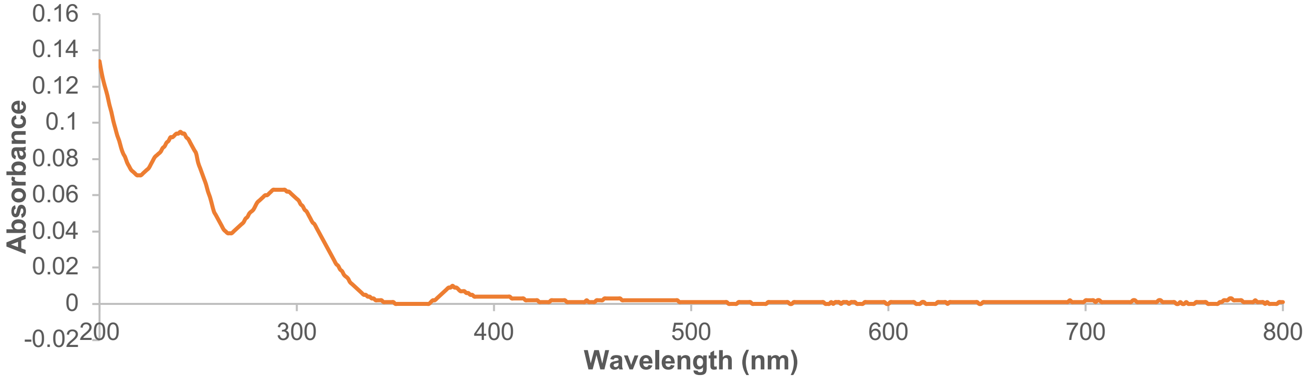

Figure S38. UV-Vis spectrum of AadUTP-PEG$_4$-DTPA **37**.

AadUTP-PEG$_{12}$-DTPA **38**

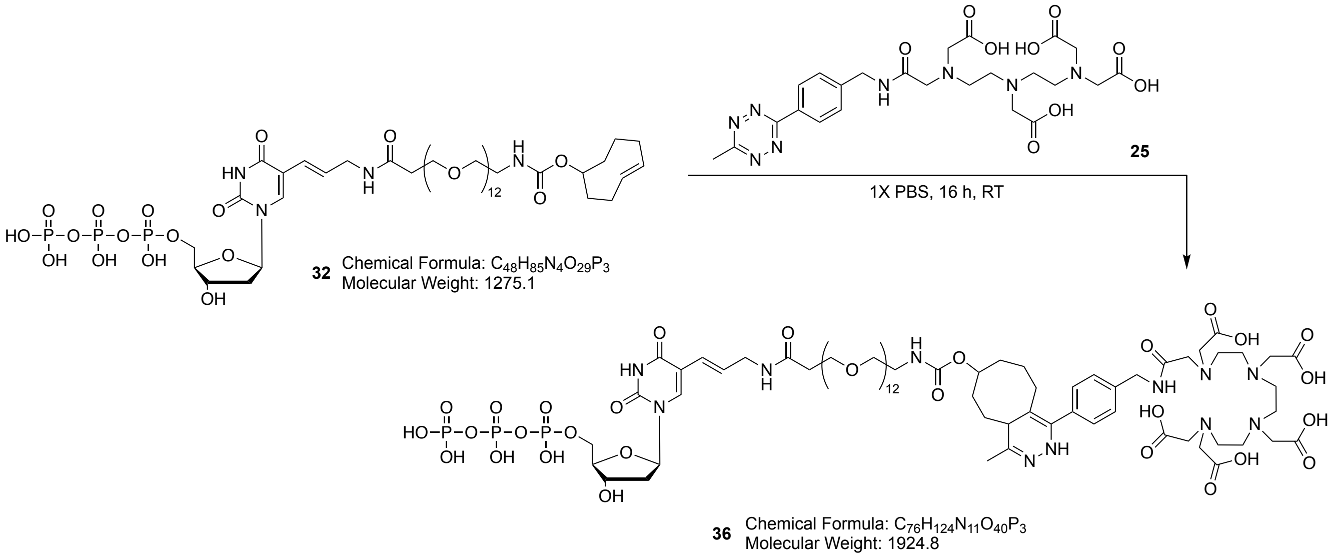

Scheme S35. Synthesis of AadUTP-PEG$_{12}$-DTPA **38**.

AadUTP-PEG$_{12}$-TCO (**34**, 250 nmol) was the starting material of this reaction, yielding 45 nmol product (**38**, 14%). t_R = 4.7 min. λ_{max} = 289 nm.

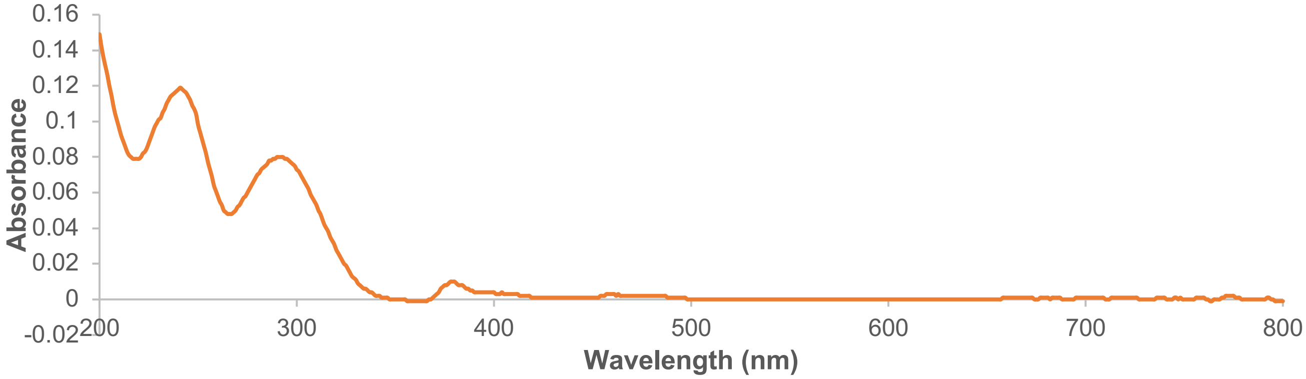

Figure S39. UV-Vis spectrum of AadUTP-PEG$_{12}$-DTPA **38**.

D. Non-radioactive synthesis of XadYTP-AHX-DOTA(M) **1a–d**, **2a–d** and XadYTP-AHX-DTPA(In) **3a**, **4a**

General non-radioactive labeling

Briefly, chelator modified nucleotide triphosphates (**1–4**, **Figure S40**, 10 nmol) was introduced to naturally abundant metal trichloride salt (10.5 nmol, 1.05 eq.) to final reaction volume (100 µL) in corresponding aqueous buffer at the specified temperature for the specified period, following HPLC separation with method **M-9**. Alternatively, purification by SepPAK cartridges were acceptable with the following protocol: to an EtOH-preactivated SepPAK C_{18} Plus Light cartridges (130 mg sorbent), the reaction crude (100 µL) was loaded, followed by an H_2O wash (1.0 mL), and finally the desired metal complexes were eluted in EtOH (500 µL). Metalated nucleotide triphosphates were pooled, dried (lyophilized for aqueous mixtures, evaporated *in vacuo* for ethanolic mixtures), and quantified via UV-Vis Spectroscopy. The conditions employed for each non-radioactive standards mirrors that of the radioactive version in **Table S5**.

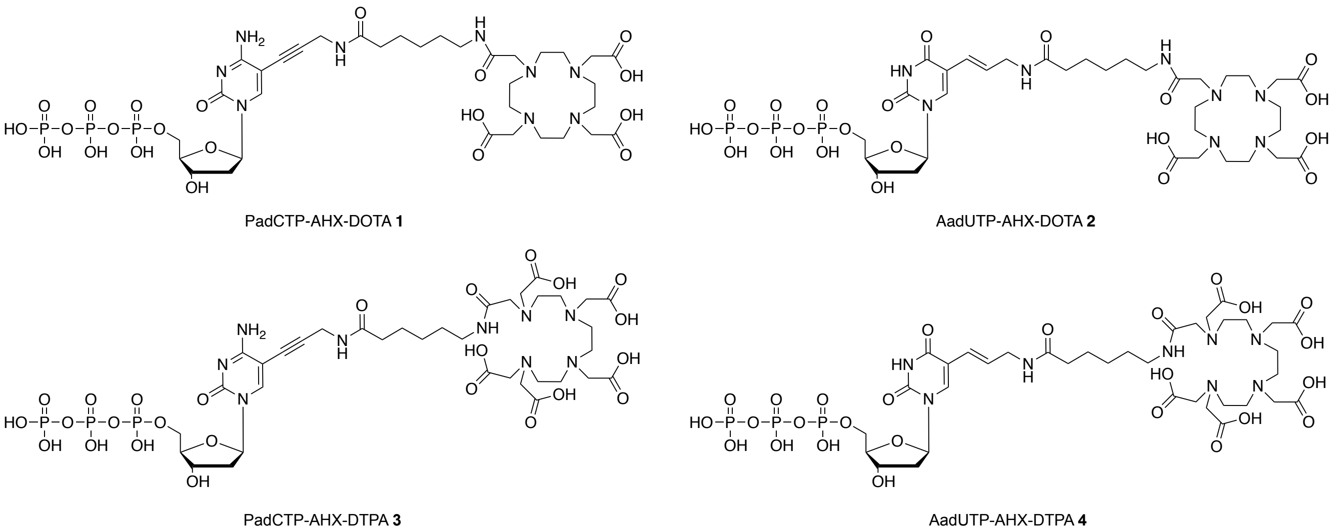

Figure S40. Structures of precursors **1–4**.

PadCTP-AHX-DOTA(Ga) ***1a***

PadCTP-AHX-DOTA (**1**, 40 nmol) was the starting material of this reaction, yielding 20 nmol product (**1a**, 50%). t_R = 9.2 min. λ_{max} = 289 nm.

HPLC Method **M-3**: Solvent A, 50 mM triethylammonium bicarbonate (TEAB) in milliQ-filtered H_2O; solvent B, MeCN; 0.0–20.0 min, 0–15% B; 20.0–20.5 min, 15–80% B; 20.5–22.5 min, 80% B; 22.5–23.0 min, 80–0% B; 23.0–25.0 min, 0% B; flow rate, 2.0 mL/min; column temperature, 19ºC – 21ºC.
HPLC Column: Eclipse XDB-C18 5 µm, 250 × 9.6 mm.

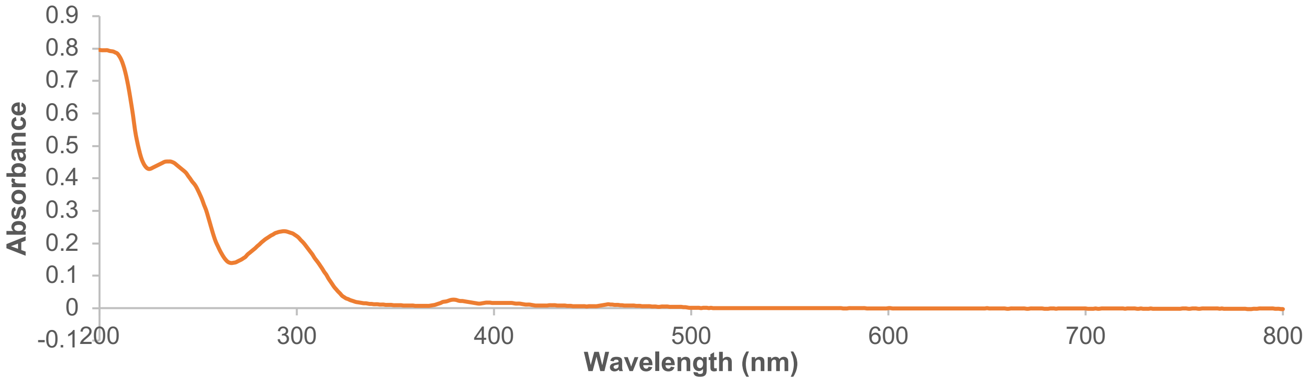

Figure S41. UV-Vis spectrum of PadCTP-AHX-DOTA(Ga) **1a**.

PadCTP-AHX-DOTA(Y) ***1b***

PadCTP-AHX-DOTA (**1**, 20 nmol) was the starting material of this reaction, yielding 14 nmol product (**1b**, 70%). t_R = 9.3 min. λ_{max} = 294 nm.

HPLC Method **M-9**: Solvent A, 50 mM triethylammonium bicarbonate (TEAB) in milliQ-filtered H_2O; solvent B, MeCN; 0.0–10.0 min, 5–20% B; 10.0–10.5 min, 20–100% B; 10.5–12.5 min,

100% B; 12.5–13.0 min, 100–5% B; 13.0–16.0 min, 5% B; flow rate, 2.0 mL/min; column temperature, 19ºC – 21ºC.
HPLC Column: Jupiter 10 µm C18 300A, 250 × 4.6 mm.

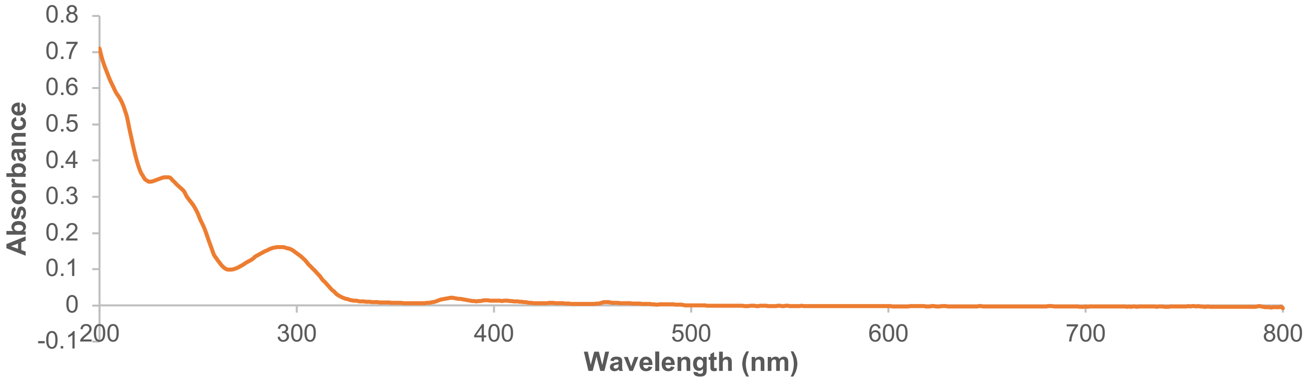

Figure S42. UV-Vis spectrum of PadCTP-AHX-DOTA(Y) **1b**.

PadCTP-AHX-DOTA(Tb) ***1c***

PadCTP-AHX-DOTA (**1**, 10 nmol) was the starting material of this reaction, yielding 2.1 nmol product (**1c**, 21%). t_R = 8.3 min. λ_{max} = 294 nm.

HPLC Method **M-9**: Solvent A, 50 mM triethylammonium bicarbonate (TEAB) in milliQ-filtered H_2O; solvent B, MeCN; 0.0–10.0 min, 5–20% B; 10.0–10.5 min, 20–100% B; 10.5–12.5 min, 100% B; 12.5–13.0 min, 100–5% B; 13.0–16.0 min, 5% B; flow rate, 2.0 mL/min; column temperature, 19ºC – 21ºC.
HPLC Column: Jupiter 10 µm C18 300A, 250 × 4.6 mm.

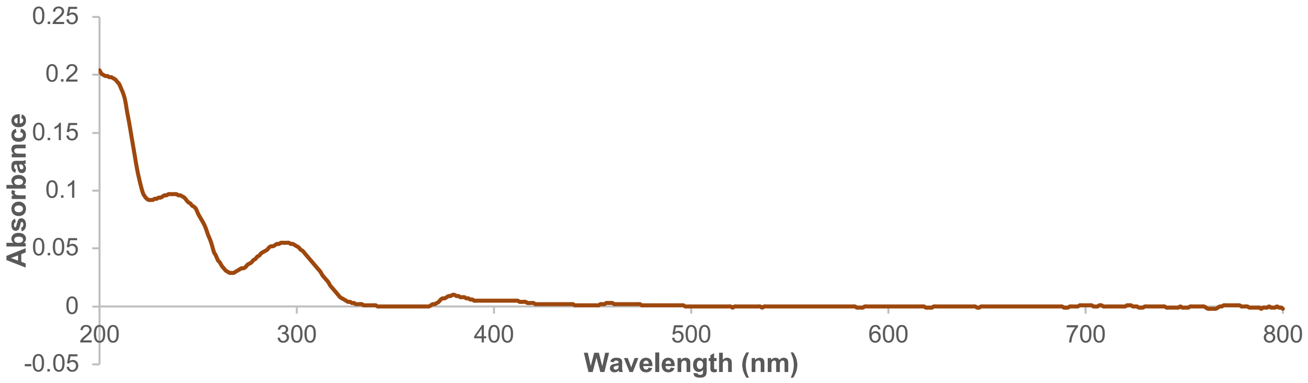

Figure S43. UV-Vis spectrum of PadCTP-AHX-DOTA(Y) **1c**.

PadCTP-AHX-DOTA(Lu) ***1d***

PadCTP-AHX-DOTA (**1**, 10 nmol) was the starting material of this reaction, yielding 7.0 nmol product (**1d**, 70%). t_R = 9.1 min. λ_{max} = 294 nm.

HPLC Method **M-9**: Solvent A, 50 mM triethylammonium bicarbonate (TEAB) in milliQ-filtered H_2O; solvent B, MeCN; 0.0–10.0 min, 5–20% B; 10.0–10.5 min, 20–100% B; 10.5–12.5 min, 100% B; 12.5–13.0 min, 100–5% B; 13.0–16.0 min, 5% B; flow rate, 2.0 mL/min; column temperature, 19ºC – 21ºC.

HPLC Column: Jupiter 10 µm C18 300A, 250 × 4.6 mm.

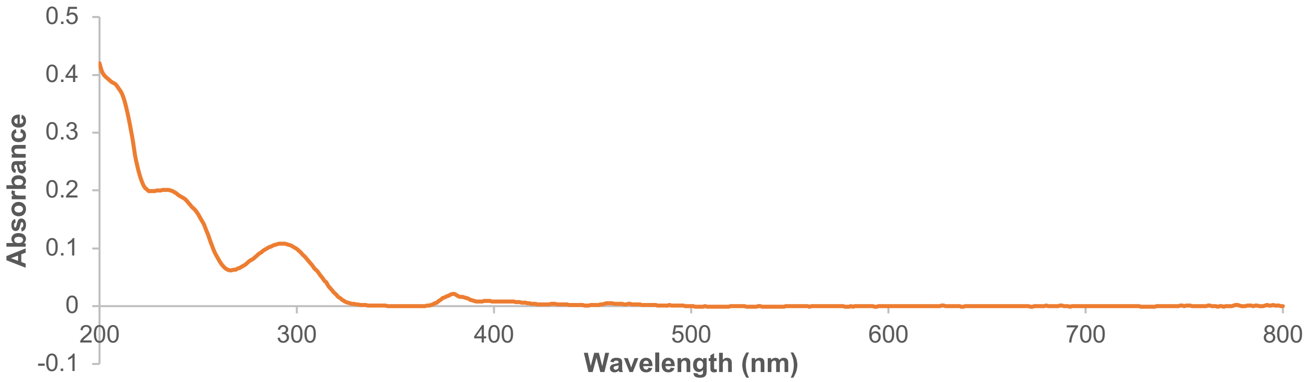

Figure S44. UV-Vis spectrum of PadCTP-AHX-DOTA(Lu) **1d**.

AadUTP-AHX-DOTA(Ga) ***2a***AadUTP-AHX-DOTA (**2**, 20 nmol) was the starting material of this reaction, yielding 8 nmol product (**2a**, 40%). t_R = 12.4 min. λ_{max} = 289 nm.

HPLC Method **M-9**: Solvent A, 50 mM triethylammonium bicarbonate (TEAB) in milliQ-filtered H_2O; solvent B, MeCN; 0.0–10.0 min, 5–20% B; 10.0–10.5 min, 20–100% B; 10.5–12.5 min, 100% B; 12.5–13.0 min, 100–5% B; 13.0–16.0 min, 5% B; flow rate, 2.0 mL/min; column temperature, 19ºC – 21ºC.
HPLC Column: Jupiter 10 µm C18 300A, 250 × 4.6 mm.

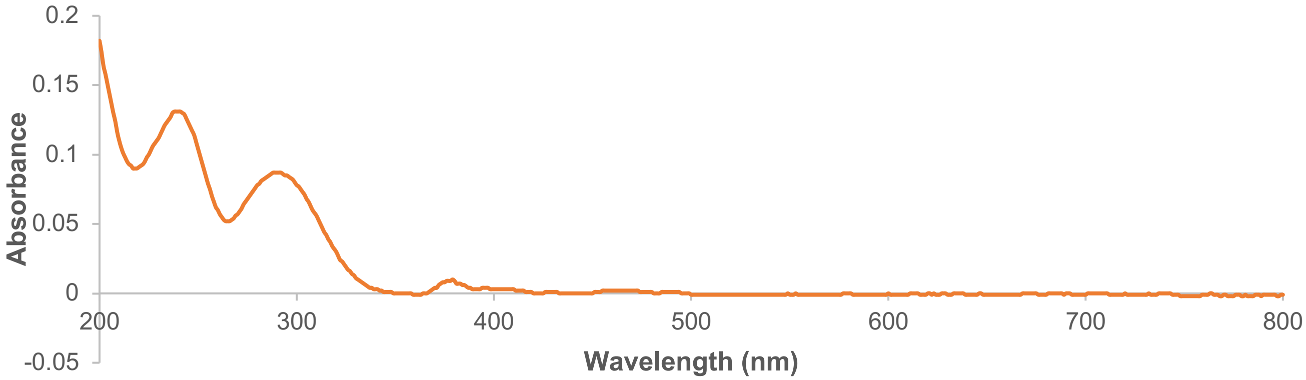

Figure S45. UV-Vis spectrum of AadUYP-AHX-DOTA(Ga) **2a**.

AadUTP-AHX-DOTA(Y) ***2b***
AadUTP-AHX-DOTA (**2**, 20 nmol) was the starting material of this reaction, yielding 16 nmol product (**2b**, 78%). t_R = 9.6 min. λ_{max} = 289 nm.

HPLC Method **M-9**: Solvent A, 50 mM triethylammonium bicarbonate (TEAB) in milliQ-filtered H_2O; solvent B, MeCN; 0.0–10.0 min, 5–20% B; 10.0–10.5 min, 20–100% B; 10.5–12.5 min, 100% B; 12.5–13.0 min, 100–5% B; 13.0–16.0 min, 5% B; flow rate, 2.0 mL/min; column temperature, 19ºC – 21ºC.
HPLC Column: Jupiter 10 µm C18 300A, 250 × 4.6 mm.

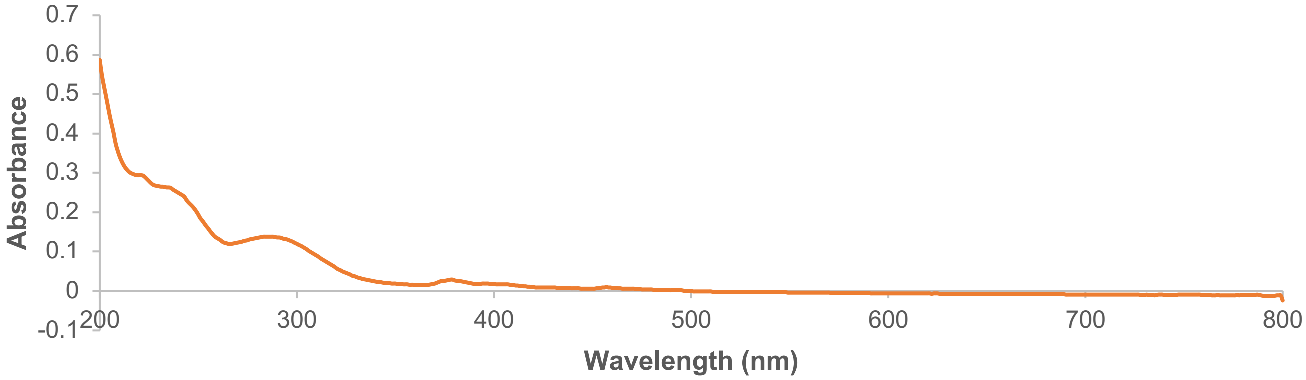

Figure S46. UV-Vis spectrum of AadUTP-AHX-DOTA(Y) **2b**.

AadUTP-AHX-DOTA(Tb) ***2c***

AadUTP-AHX-DOTA (**2**, 10 nmol) was the starting material of this reaction, yielding 3.6 nmol product (**2c**, 36%). t_R = 9.2 min. λ_{max} = 289 nm.

HPLC Method **M-9**: Solvent A, 50 mM triethylammonium bicarbonate (TEAB) in milliQ-filtered H_2O; solvent B, MeCN; 0.0–10.0 min, 5–20% B; 10.0–10.5 min, 20–100% B; 10.5–12.5 min, 100% B; 12.5–13.0 min, 100–5% B; 13.0–16.0 min, 5% B; flow rate, 2.0 mL/min; column temperature, 19ºC – 21ºC.
HPLC Column: Jupiter 10 µm C18 300A, 250 × 4.6 mm.

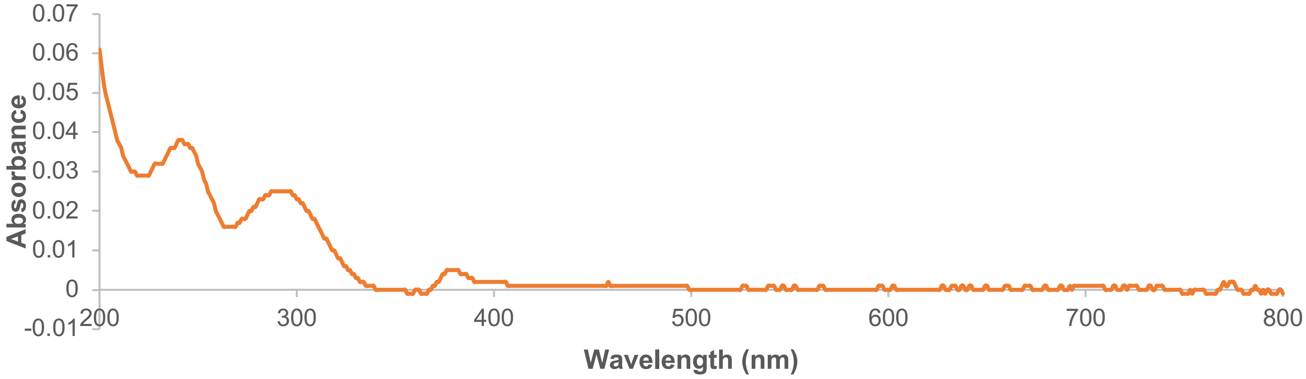

Figure S47. UV-Vis spectrum of AadUTP-AHX-DOTA(Tb) **2c**.

AadUTP-AHX-DOTA(Lu) ***2d***

AadUTP-AHX-DOTA (**2**, 10 nmol) was the starting material of this reaction, yielding 6.6 nmol product (**2d**, 66%). t_R = 8.0 min. λ_{max} = 289 nm.

HPLC Method **M-9**: Solvent A, 50 mM triethylammonium bicarbonate (TEAB) in milliQ-filtered H_2O; solvent B, MeCN; 0.0–10.0 min, 5–20% B; 10.0–10.5 min, 20–100% B; 10.5–12.5 min, 100% B; 12.5–13.0 min, 100–5% B; 13.0–16.0 min, 5% B; flow rate, 2.0 mL/min; column temperature, 19ºC – 21ºC.
HPLC Column: Jupiter 10 µm C18 300A, 250 × 4.6 mm.

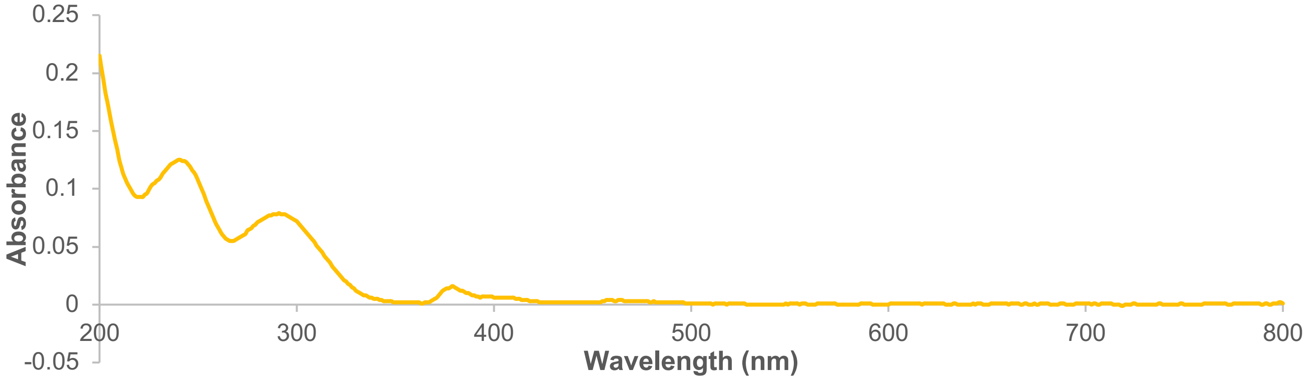

Figure S48. UV-Vis spectrum of AadUTP-AHX-DOTA(Lu) **2d**.

PadCTP-AHX-DTPA(In) ***3a***

PadCTP-AHX-DTPA (**3**, 10 nmol) was the starting material of this reaction, yielding 2.7 nmol product (**3a**, 27%). t_R = 10.3 min. λ_{max} = 294 nm.

HPLC Method **M-9**: Solvent A, 50 mM triethylammonium bicarbonate (TEAB) in milliQ-filtered H_2O; solvent B, MeCN; 0.0–10.0 min, 5–20% B; 10.0–10.5 min, 20–100% B; 10.5–12.5 min, 100% B; 12.5–13.0 min, 100–5% B; 13.0–16.0 min, 5% B; flow rate, 2.0 mL/min; column temperature, 19ºC – 21ºC.
HPLC Column: Jupiter 10 µm C18 300A, 250 × 4.6 mm.

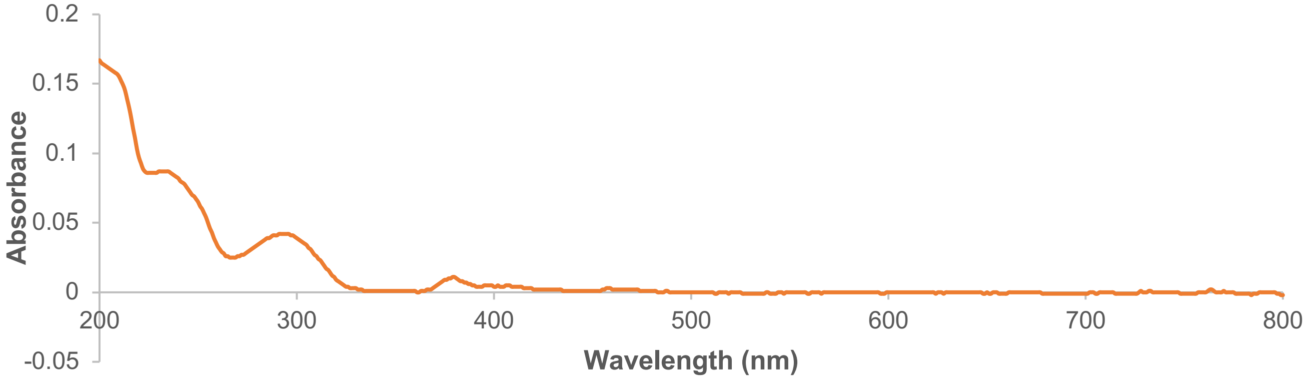

Figure S49. UV-Vis spectrum of PadCTP-AHX-DTPA(In) **3a**.

AadUTP-AHX-DTPA(In) ***4a***

AadUTP-AHX-DTPA (**4**, 10 nmol) was the starting material of this reaction, yielding 4.9 nmol product (**4a**, 49%). t_R = 10.3 min. λ_{max} = 289 nm.

HPLC Method **M-9**: Solvent A, 50 mM triethylammonium bicarbonate (TEAB) in milliQ-filtered H_2O; solvent B, MeCN; 0.0–10.0 min, 5–20% B; 10.0–10.5 min, 20–100% B; 10.5–12.5 min, 100% B; 12.5–13.0 min, 100–5% B; 13.0–16.0 min, 5% B; flow rate, 2.0 mL/min; column temperature, 19ºC – 21ºC.
HPLC Column: Jupiter 10 µm C18 300A, 250 × 4.6 mm.

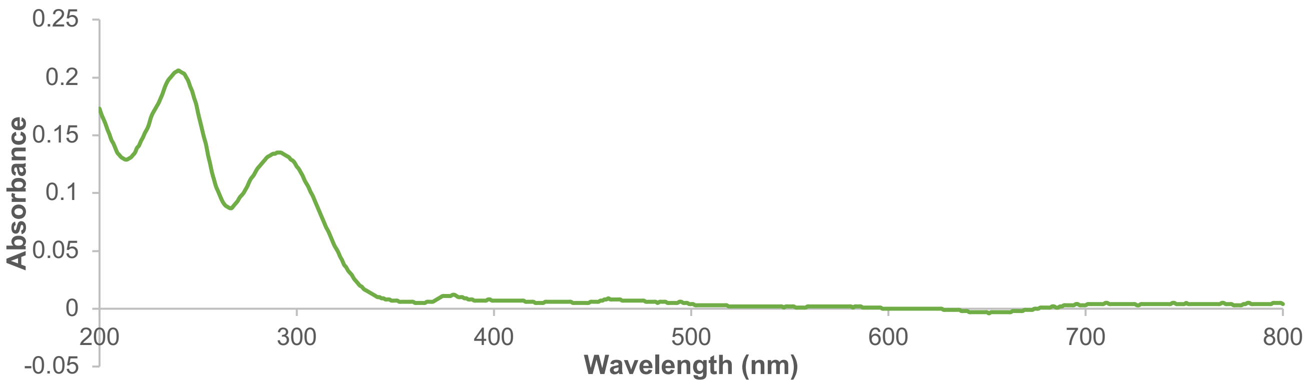

Figure S50. UV-Vis spectrum of AadUTP-AHX-DTPA(In) **4a**.

NMR Chelation Test

To first approximation, a chelator-modified triphosphate has two possible sites of metal binding. The first choice agrees with the major premise of this manuscript, where the functional groups linked at C^5 position of a nucleobase acts as the chelator, in line with other radiopharmaceuticals in human use, such as Netspot and Lutathera. However, the alternative choice of the triphosphate group is also reasonable, as evidenced by well-known Mg^{2+}:ATP interactions. To demonstrate proof-of-concept, Lu^{3+} was incubated with dCTP and DOTA in order to study the preference of metal binding to either groups via ^{31}P NMR Spectroscopy.

Entry	$[Lu^{3+}]$ (mM)	[dCTP] (mM)	[DOTA] (mM)	PPT[1]
1	3	3	3	-
2	3	6	-	+
3	3	3	-	++
4	-	3	-	-

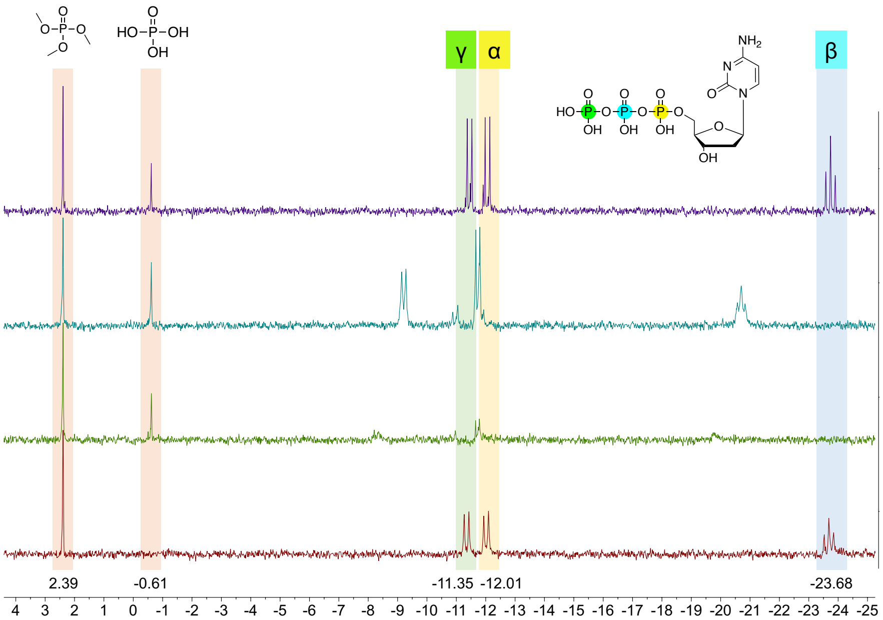

Figure S51. Coordination competition between the triphosphate and DOTA moieties. [1] PPT refers to tendencies of white precipitate formation prior to 95ºC heating. The precipitates did not solubilize during heating.

To final volume of 500 µL in a 1.5 mL microcentrifuge tube, $^{nat}LuCl_3 \cdot 6H_2O$ (1.5 µmol, 3 mM), dCTP (1.5–3.0 µmol, 3–6 mM, 1–2 eq.), DOTA (0–1.5 µmol, 0–3 mM, 0–1 eq.), trimethylphosphate (1.5 µmol, 3 mM, 1 eq.) was dissolved in NaOAc (100 mM), and then heated at 95ºC for 5 min to mirror radiolabeling conditions. Contents of each tube was transferred to NMR tubes, and ^{31}P {^1H} NMR spectra was acquired on a 300 MHz spectrometer at 256 scans, with trimethylphosphate serving as internal standard at 2.39 ppm.

Entry 4 (**Figure S51**) demonstrate a typical $^{31}P\ \{^{1}H\}$ NMR spectra of dCTP (121 MHz, H_2O) δ -11.35 (d, *J* = 19.8 Hz, P_{γ}), -12.01 (d, *J* = 19.6 Hz, P_{α}), -23.68 (t, *J* = 19.5 Hz, P_{β}). Without the presence of DOTA, precipitation occurred when dCTP was incubated with Lu^{3+} at stoichiometric parity (entry 2). Interestingly, with super-stoichiometric quantities of Lu^{3+}, the extent of precipitation was reduced (entry 3). Nonetheless, when a choice of DOTA and triphosphates was provided, the metal ion prefers chelation with DOTA, as evidenced by the lack of NMR shift perturbation in entry 1.

HPLC Stability Test

One methodological hurdle surmounted by this work was to develop a gel-based method to demonstrate successful radiosynthesis of metalated DNA. Inspired by dideoxy sequencing that enabled the human genome project, our initial attempts to directly incorporate radioactive nucleotide triphosphates following denaturing PAGE and autoradiography failed to materialize images that has concordant radioactive and fluorescent signals. On deeper examination, the samples with radiolabeled DNA appears identical in migration signatures as unincorporated radioactive nucleotide triphosphates. It led us to hypothesize that dechelation occurred during manipulative steps between primer extension and image visualization. To test this hypothesis, a previous described PSMA-binding radioconjugate [^{68}Ga]Ga-HTK03149 was exposed to the three steps under scrutiny in **Figure S52**:[31] (A) DTT in NEBuffer 2 at 37ºC for 1 h, encountered during primer extension; (B) NaOH, 1 min, RT, as denaturant in standard DNA gel loading buffer; and (C) 0.5 X TBE, 45 min, 65 ºC, during gel electrophoresis. (D) Water served as negative control for the purpose of this experiment. Radio-HPLC revealed that conditions (B) and (C) leads to degradation of the desired radio-complex. Interestingly, work by Roivainen *et al*.[8a] demonstrated technical feasibility of visualizing DNA in a non-denaturing fashion. Therefore, we concluded that both basic conditions, and EDTA challenge during electrophoresis, led to this artifact.

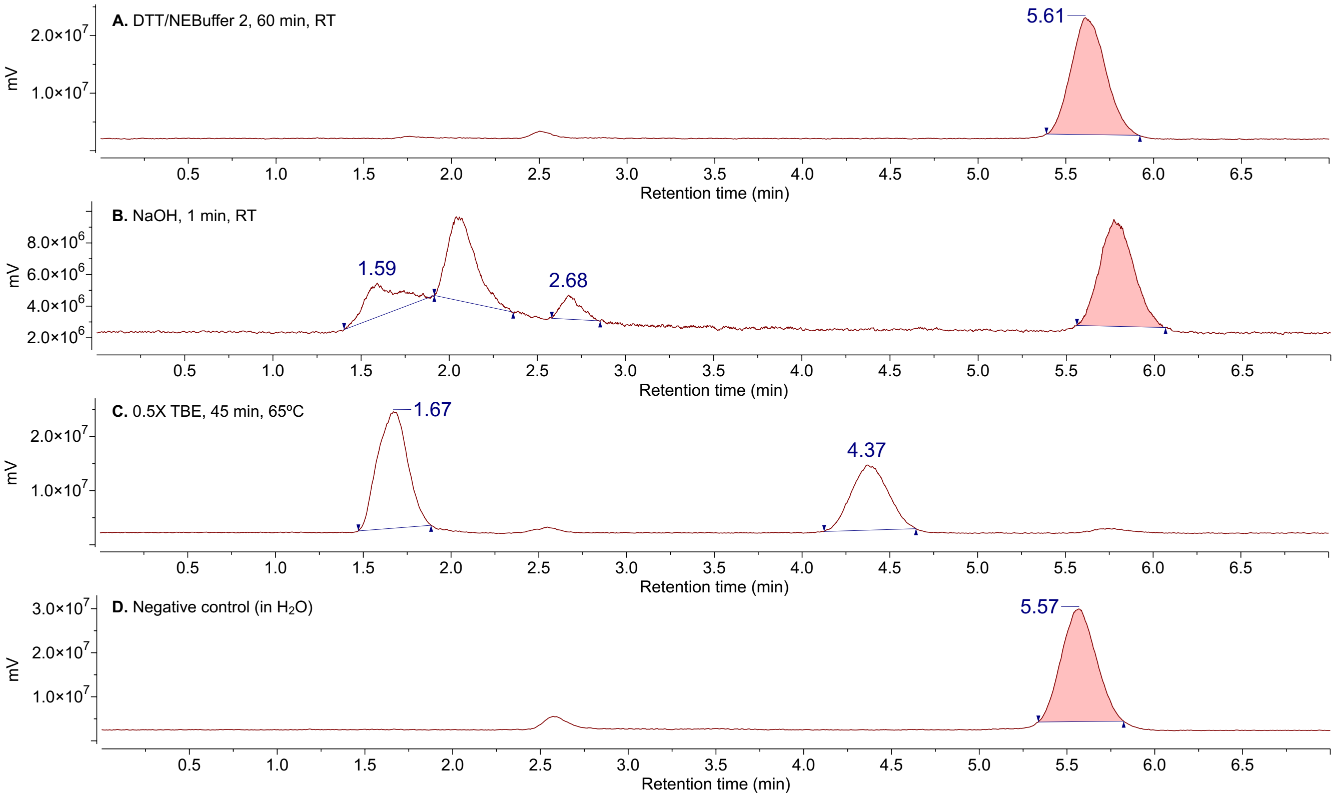

Figure S52. [^{68}Ga]Ga-HTK03149 stability study via radio-HPLC in selected conditions. (**A**) NEBuffer 2, 1X, 60 min, incubated in RT; (**B**), NaOH (10 mM), 1 min, incubated in RT; (**C**) TBE, 0.5X, 45 min, incubated in 65ºC; (**D**) H_2O as negative control, 60 min, incubated in RT.

Aliquots of [^{68}Ga]Ga-HTK03149 (1 MBq) was dissolved in 4 different solutions for stability testing, namely (A) NEBuffer 2 (1X contains 10 mM $MgCl_2$ and 1 mM DTT), (B) NaOH (10 mM), (C) TBE electrophoresis buffer (0.5 X contains 1.25 mM EDTA) and (D) H_2O serving as negative control. These mixtures were incubated as indicated in **Figure S52**; samples of 500 kBq was analyzed by radio-HPLC.

Radiosynthesis

E. Radiosynthesis of XadYTP-AHX-DOTA(M) and XadYTP-AHX-DTPA(In) series

General radioactive labeling

No carrier added [^{68}Ga]-$GaCl_3$ was obtained following elution from a [68]Ge generator (ITM, Munich, Germany), and purified via a DGA resin column from Eichrom Technologies LLC (Lisle, IL, USA) per manufacturer instructions. Otherwise, all other commercially purchased radioactive metal salts (0.2–400 MBq) was first added with corresponding carrier (10.5 nmol) to reduce operator exposure to radiation. Radiosynthetic precursors (10 nmol) was introduced reaction buffer, and radioactivity was finally added to a total reaction volume of 100 µL, following HPLC separation. The fraction containing the desired radioactive complex was diluted in 100 mL milliQ-grade H_2O and loaded to an EtOH-preactivated SepPAK Cartridge (C_{18}, 130 mg sorbent, Waters WAT023501, Framingham, MA, USA). The cartridge was eluted with EtOH (100 µL) to obtain a working solution of the radioactive nucleotide triphosphate. The radiolabeling conditions mirror that of non-radioactive syntheses, and are summarized in **Table S5**.

Table S5. Summary of radiolabeling conditions.

Molecule		Temp.	Time	pH	[Buffer]	Buffer
[^{68}Ga]-PadCTP-AHX-DOTA(Ga)	**^{68}Ga-1a**	95ºC	15 min	4.5	2.0 M	NaOAc
[^{161}Tb]-PadCTP-AHX-DOTA(Tb)	**^{161}Tb-1c**	95ºC	15 min	6.0	100 mM	NH_4OAc
[^{177}Lu]-PadCTP-AHX-DOTA(Lu)	**^{177}Lu-1d**	95ºC	15 min	4.5	100 mM	NaOAc
[^{161}Tb]-AadUTP-AHX-DOTA(Tb)	**^{161}Tb-2c**	95ºC	15 min	6.0	100 mM	NH_4OAc
[^{177}Lu]-AadUTP-AHX-DOTA(Lu)	**^{177}Lu-2d**	95ºC	15 min	4.5	100 mM	NaOAc
[^{111}In]-PadCTP-AHX-DTPA(In)	**^{111}In-3a**	RT	30 min	7.4	1X	PBS
[^{111}In]-AadUTP-AHX-DTPA(In)	**^{111}In-4a**	RT	30 min	7.4	1X	PBS

[^{68}Ga]-PadCTP-AHX-DOTA(Ga) **^{68}Ga-1a**

Purified NCA [^{68}Ga]-$GaCl_3$ was added to PadCTP-AHX-DOTA **1** (10 nmol) to afford [^{68}Ga]-PadCTP-AHX-DOTA(Ga) **^{68}Ga-1a**. t_R = 10.6 min.

HPLC Method **M-7**: Solvent A, 50 mM triethylammonium bicarbonate (TEAB) in milliQ-filtered H_2O; solvent B, MeCN; 0.0–20.0 min, 5–20% B; 20.0–20.5 min, 20–100% B; 20.5–22.5 min, 100% B; 22.5–23.0 min, 100–5% B; 23.0–27.0 min, 5% B; flow rate, 1.0 mL/min; column temperature, 19ºC – 21ºC.
HPLC Column: Jupiter 10 µm C18 300A, 250 × 4.6 mm.

[^{161}Tb]-PadCTP-AHX-DOTA(Tb) **^{161}Tb-1c**

Carrier added [^{161}Tb]-$TbCl_3$ was added to PadCTP-AHX-DOTA **1** (10 nmol) to afford [^{161}Tb]-PadCTP-AHX-DOTA(Tb) **^{161}Tb-1c**. t_R = 9.4 min.

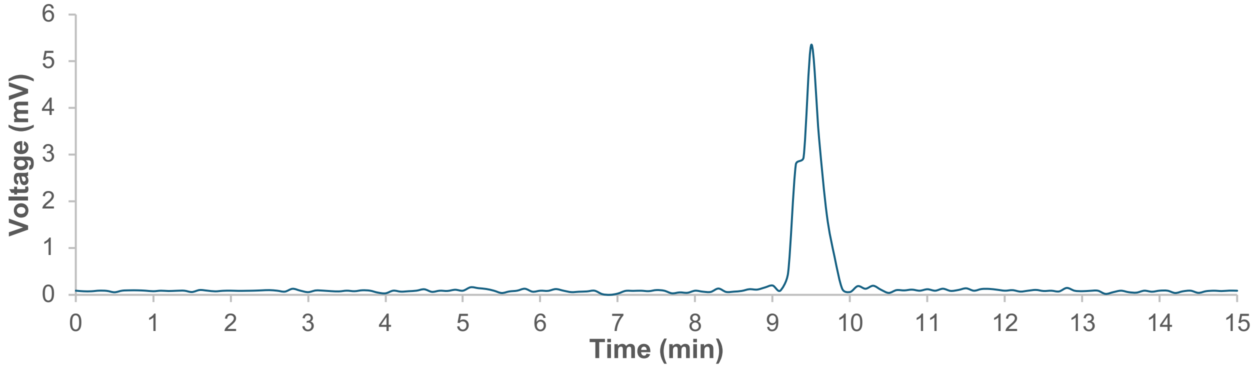

Figure S53. Radioactive HPLC trace of [^{161}Tb]-PadCTP-AHX-DOTA(Tb) **^{161}Tb-1c**.

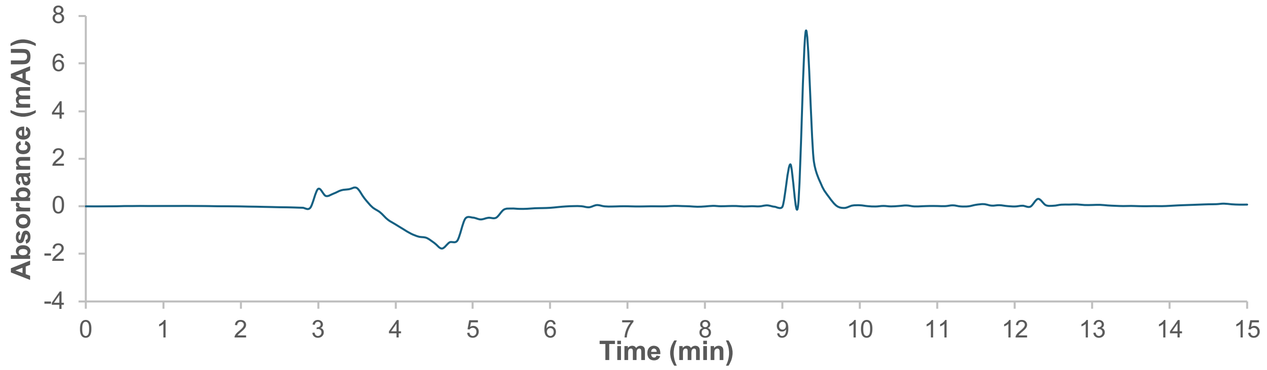

Figure S54. UV (λ = 289 nm) HPLC trace of [^{161}Tb]-PadCTP-AHX-DOTA(Tb) **^{161}Tb-1c**.

HPLC Method **M-7**: Solvent A, 50 mM triethylammonium bicarbonate (TEAB) in milliQ-filtered H_2O; solvent B, MeCN; 0.0–20.0 min, 5–20% B; 20.0–20.5 min, 20–100% B; 20.5–22.5 min, 100% B; 22.5–23.0 min, 100–5% B; 23.0–27.0 min, 5% B; flow rate, 1.0 mL/min; column temperature, 19ºC – 21ºC.
HPLC Column: Jupiter 10 µm C18 300A, 250 × 4.6 mm.

[^{177}Lu]-PadCTP-AHX-DOTA(Lu) **^{177}Lu-1d**

Carrier added [^{177}Lu]-$LuCl_3$ was added to PadCTP-AHX-DOTA **1** (10 nmol) to afford [^{177}Lu]-PadCTP-AHX-DOTA(Lu) **^{177}Lu-1d**. t_R = 7.9 min.

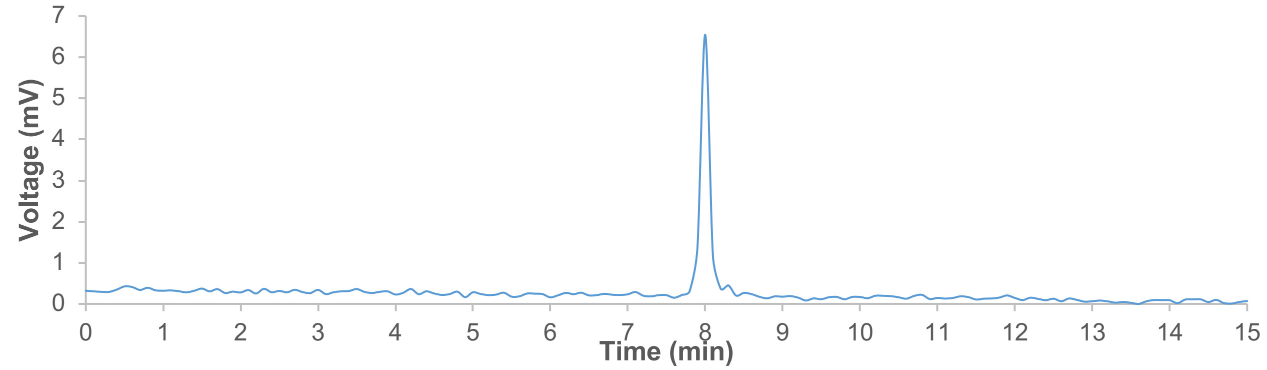

Figure S55. Radioactive HPLC trace of [^{177}Lu]-PadCTP-AHX-DOTA(Lu) **^{177}Lu-1d**.

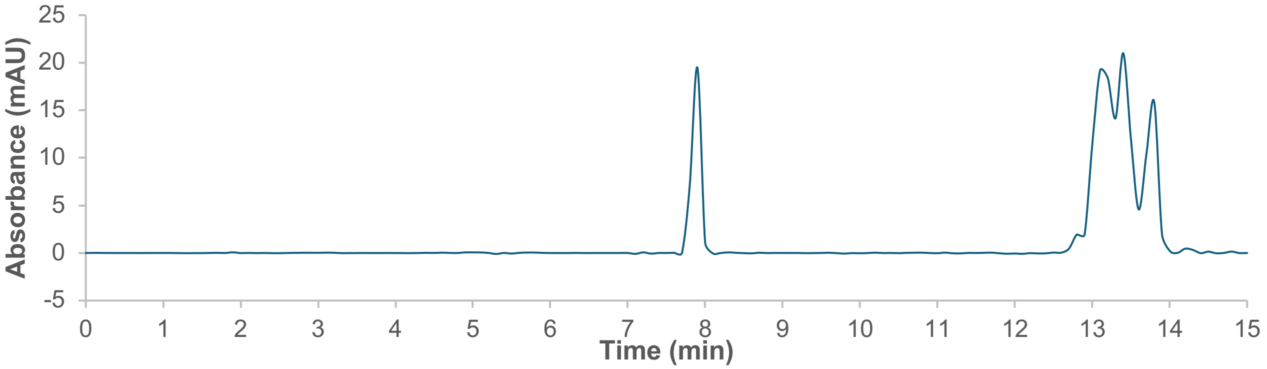

Figure S56. UV (λ = 289 nm) HPLC trace of [^{177}Lu]-PadCTP-AHX-DOTA(Lu) **^{177}Lu-1d**.

HPLC Method **M-10**: Solvent A, 50 mM triethylammonium bicarbonate (TEAB) in milliQ-filtered H_2O; solvent B, MeCN; 0.0–10.0 min, 0–15% B; 10.0–10.5 min, 15–80% B; 10.5–12.5 min, 80% B; 12.5–13.0 min, 80–0% B; 13.0–15.0 min, 0% B; flow rate, 2.0 mL/min; column temperature, 19ºC – 21ºC.
HPLC Column: Jupiter 10 µm C18 300A, 250 × 4.6 mm.

[^{161}Tb]-AadUTP-AHX-DOTA(Tb) ^{161}**Tb-2c**

Carrier added [^{161}Tb]-$TbCl_3$ was added to AadUTP-AHX-DOTA **2** (10 nmol) to afford [^{161}Tb]-AadUTP-AHX-DOTA(Lu) ^{161}**Tb-2c**. t_R = 10.4 min.

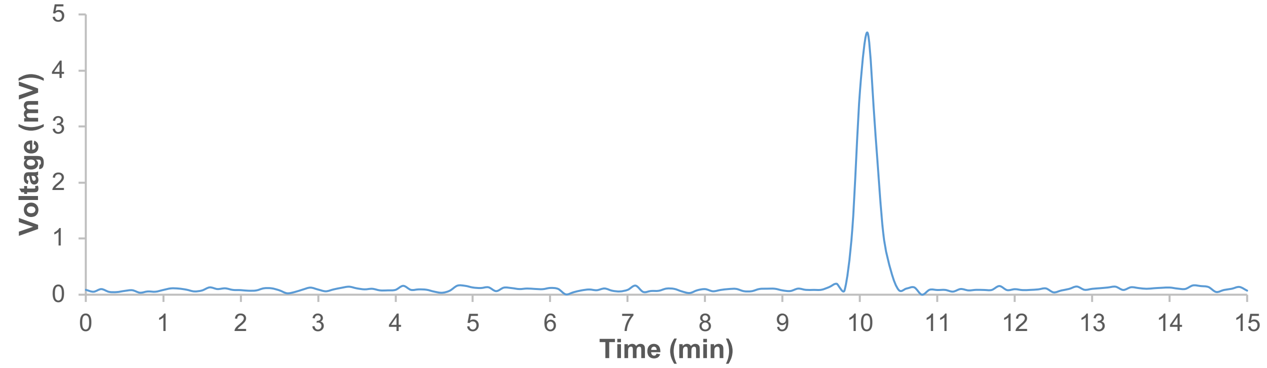

Figure S57. Radioactive HPLC trace of [^{161}Tb]-AadUTP-AHX-DOTA(Tb) ^{161}**Tb-2c**.

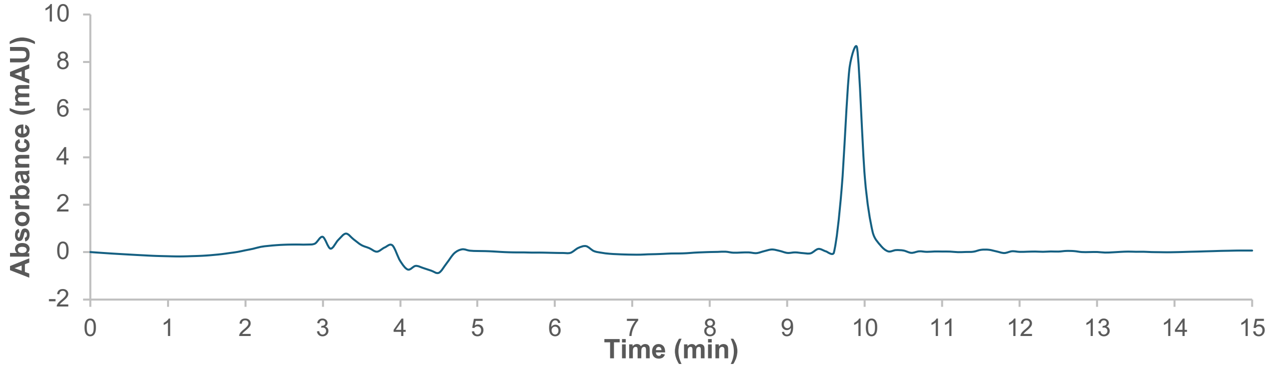

Figure S58. UV (λ = 289 nm) HPLC trace of [^{161}Tb]-AadUTP-AHX-DOTA(Tb) ^{161}**Tb-2c**.

HPLC Method **M-7**: Solvent A, 50 mM triethylammonium bicarbonate (TEAB) in milliQ-filtered H_2O; solvent B, MeCN; 0.0–20.0 min, 5–20% B; 20.0–20.5 min, 20–100% B; 20.5–22.5 min, 100% B; 22.5–23.0 min, 100–5% B; 23.0–27.0 min, 5% B; flow rate, 1.0 mL/min; column temperature, 19ºC – 21ºC.
HPLC Column: Jupiter 10 µm C18 300A, 250 × 4.6 mm.

[^{177}Lu]-AadUTP-AHX-DOTA(Lu) ^{177}**Lu-2d**

Carrier added [^{177}Lu]-$LuCl_3$ was added to AadUTP-AHX-DOTA **2** (10 nmol) to afford [^{177}Lu]-AadUTP-AHX-DOTA(Lu) ^{177}**Lu-2d**. t_R = 8.1 min.

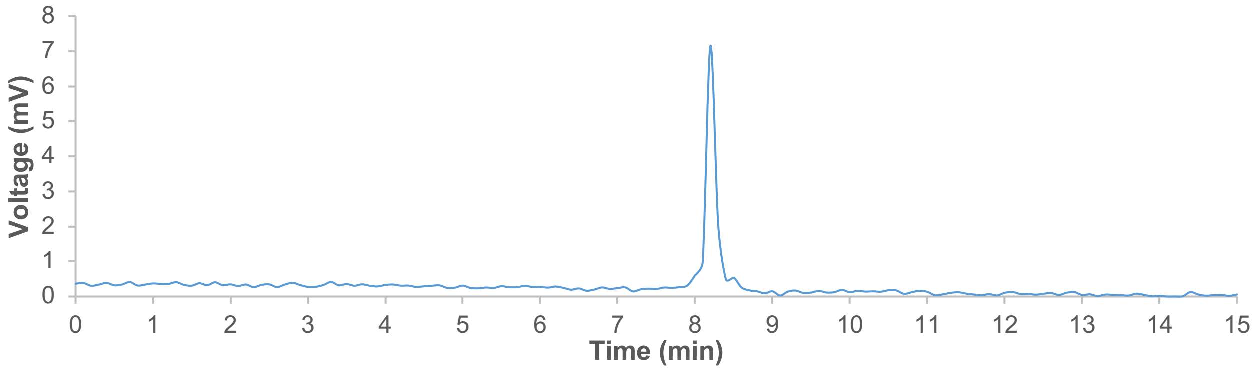

Figure S59. Radioactive HPLC trace of [^{177}Lu]-AadUTP-AHX-DOTA(Lu) **^{177}Lu-2d**.

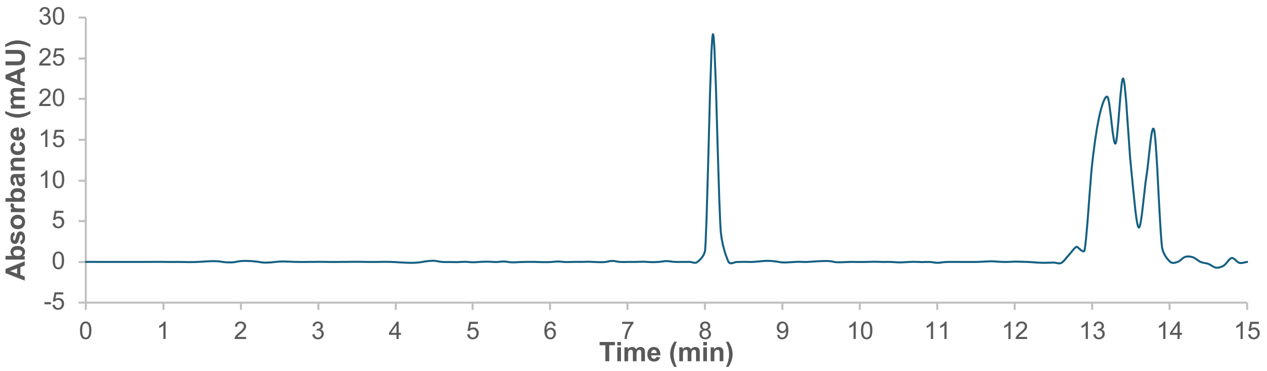

Figure S60. UV (λ = 289 nm) HPLC trace of [^{177}Lu]-AadUTP-AHX-DOTA(Lu) **^{177}Lu-2d**.

HPLC Method **M-10**: Solvent A, 50 mM triethylammonium bicarbonate (TEAB) in milliQ-filtered H_2O; solvent B, MeCN; 0.0–10.0 min, 0–15% B; 10.0–10.5 min, 15–80% B; 10.5–12.5 min, 80% B; 12.5–13.0 min, 80–0% B; 13.0–15.0 min, 0% B; flow rate, 2.0 mL/min; column temperature, 19ºC – 21ºC.
HPLC Column: Jupiter 10 µm C18 300A, 250 × 4.6 mm.

[^{111}In]-PadCTP-AHX-DTPA(In) **^{111}In-3a**

Carrier added [^{111}In]-$InCl_3$ was added to PadCTP-AHX-DTPA **3** (10 nmol) to afford [^{111}In]-PadCTP-AHX-DTPA(In) **^{111}In-3a**. t_R = 10.2 min.

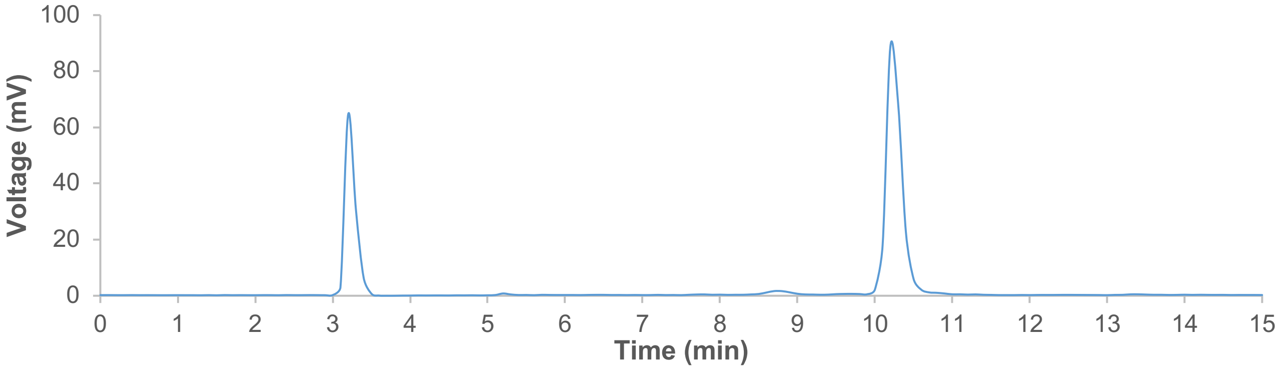

Figure S61. Radioactive HPLC trace of [^{111}In]-PadCTP-AHX-DTPA(In) **^{111}In-3a**.

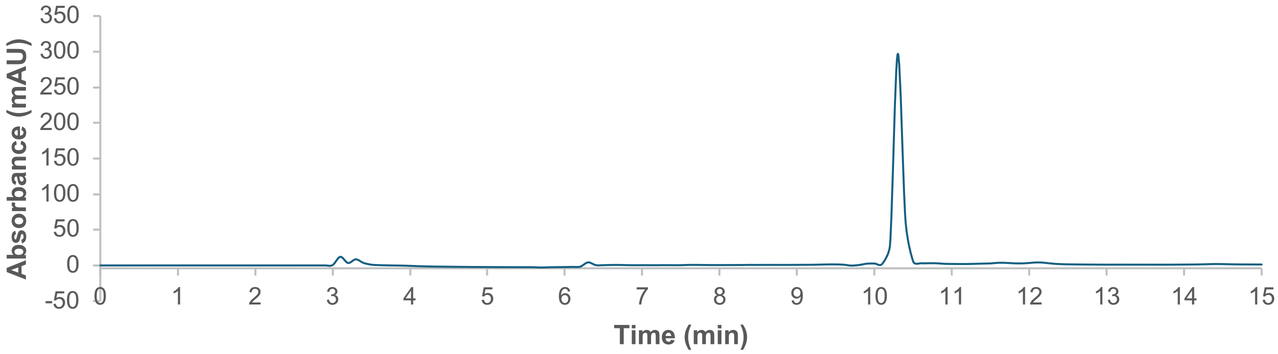

Figure S62. UV (λ = 289 nm) HPLC trace of [^{111}In]-PadCTP-AHX-DTPA(In) **^{111}In-3a**.

HPLC Method **M-7**: Solvent A, 50 mM triethylammonium bicarbonate (TEAB) in milliQ-filtered H_2O; solvent B, MeCN; 0.0–20.0 min, 5–20% B; 20.0–20.5 min, 20–100% B; 20.5–22.5 min, 100% B; 22.5–23.0 min, 100–5% B; 23.0–27.0 min, 5% B; flow rate, 1.0 mL/min; column temperature, 19ºC – 21ºC.
HPLC Column: Jupiter 10 μm C18 300A, 250 × 4.6 mm.

[^{111}In]-AadUTP-AHX-DTPA(In) **^{111}In-4a**Carrier added [^{111}In]-$InCl_3$ was added to AadUTP-AHX-DTPA **4** (10 nmol) to afford [^{111}In]-AadUTP-AHX-DTPA(In) **^{111}In-4a**. t_R = 10.3 min.

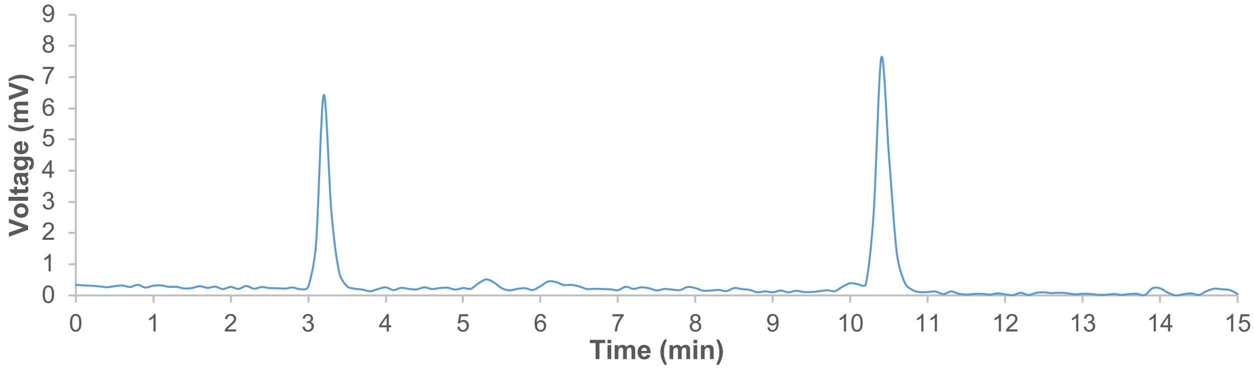

Figure S63. Radioactive HPLC trace of [^{111}In]-AadUTP-AHX-DTPA(In) **^{111}In-4a**.

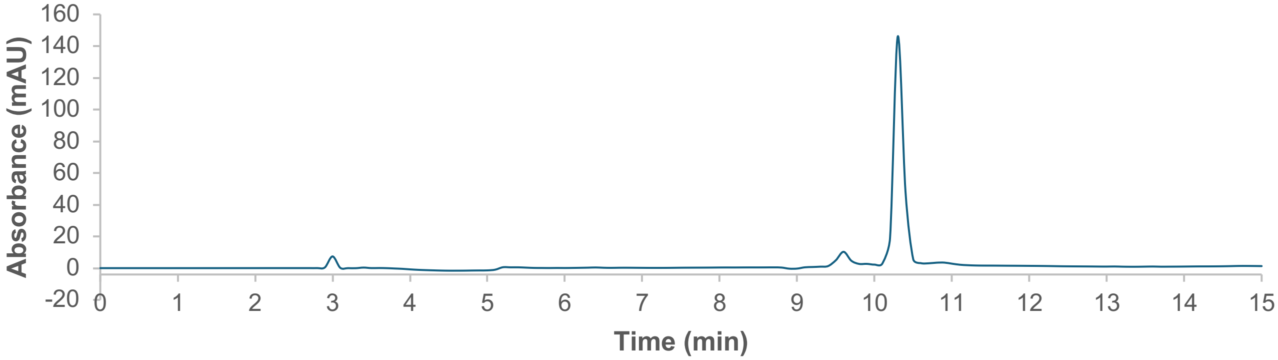

Figure S64. UV (λ = 289 nm) HPLC trace of [^{111}Tb]-PadCTP-AHX-DOTA(Tb) **^{111}In-1c**.

HPLC Method **M-7**: Solvent A, 50 mM triethylammonium bicarbonate (TEAB) in milliQ-filtered H_2O; solvent B, MeCN; 0.0–20.0 min, 5–20% B; 20.0–20.5 min, 20–100% B; 20.5–22.5 min,

100% B; 22.5–23.0 min, 100–5% B; 23.0–27.0 min, 5% B; flow rate, 1.0 mL/min; column temperature, 19ºC – 21ºC.
HPLC Column: Jupiter 10 µm C18 300A, 250 × 4.6 mm.

DNA Sequences and Gel Electrophoresis

Oligonucleotide Sequences

Table S6. Oligonucleotide sequences used in this study. All oligomers were purchased from Integrated DNA Technologies (IDT).

Index	5′-modification	Sequence (5′ → 3′)<br>1 5 10 15 20 25 28
**ON-1**	Phosphate	TCATCCCTTGCTAACAAAGCTTGCACGC
**ON-2**	Phosphate	TCATCGCATCCTAACAAAGCTTGCACGC
**ON-3**	Phosphate	TCATCGCTTGCTAACAAAGCTTGCACGC
**ON-4**	Phosphate	TCATCGCATGCTAACAAAGCTTGCACGC
**ON-5**	Cy5	GCGTGCAAGCTTTGTT
**ON-6**	6-FAM	AGCCTGTTGTGAGCCTCCTAAC
**ON-7**	Phosphate	TCATCGGGGGCTAACAAAGAAAACACGC
**ON-8**	Phosphate	TCATCGCATGCTAACAAAGCTTGCACGCT_{30}
**ON-9**	Biotin	TCATCGCATGCTAACAAAGCTTGCACGC
**ON-10**	OH	GAACAAAGCTTGCACGC
**ON-11**	OH	AAACAAAGCTTGCACGC

Polyacrylamide Gel Electrophoresis (PAGE)

Purification of Oligonucleotides by Denaturing PAGE

All samples for denaturing analysis were first formulated in DNA Denaturing Loading Buffer **B-5** and heated at 95ºC for 5 min prior to analysis. Denaturing gels were casted in 29:1 acrylamide: bisacrylamide, 6 M urea, TEMED, and APS in 1X TBE **B-6**. Gel sizes and running conditions depended on the length of oligonucleotide purified.

Primers of length shorter than or equal to 24 nt were purchased from Integrated DNA Technologies (IDT, Newark, NJ, USA) with standard desalting, followed by excising a large 20% denaturing polyacrylamide gel (420 × 330 × 0.4 mm, 40 W, 2300–3000 V, 5 h). Oligonucleotides of 25–59 nt were purchased and purified similarly on a small 20% denaturing polyacrylamide gel (170 × 165 × 0.75 mm, 17 W, 650 V, 1 h). Bands were exposed by UV shadowing and excised with a sterile blade. The gel slabs were disintegrated with a sterile pipette tip to increase surface area following elution in Gel Elution Buffer **B-7** (1 mL × 3). The pooled solutions were concentrated to less than 100 µL, followed by acidification with NaOAc (3.0 M, 10 µL) and precipitated in ice cold EtOH (100%). The pellets were further washed by EtOH (70%, aq.) and air-dried. Quantification was completed on a QuickDrop UV-Vis spectrophotometer. Oligonucleotides exceeding or equal to 60 nt in length were purchased from IDT with standard desalting, followed by EtOH/ NaOAc precipitation prior to use.

Analysis of Denatured DNA Strands

Denaturing urea gels and samples were prepared similarly as PAGE purification at 15% polyacrylamide, in gel plates that are compatible with mini-PROTEAN® cassettes (83 × 73 ×

0.75 mm, 400 V, 20–22 min). As a fiduciary marker, xylene cyanol FF was used together with bromophenol blue to form a surrogate ladder. Gel visualization was completed using the Typhoon™ 5 Biomolecular Imager (Cytiva, Marlborough, MA, USA). For fluorescein and Cy5 detection, Cy3 and Cy5 modes were chosen respectively. For autoradiography, the manufacturer's default settings for phosphorimaging were used directly without modifications.

Analysis of Native DNA Strands

For the purpose of this manuscript, native gels were casted with urea, but the samples were loaded in native loading buffer **B-1**, and separated in native running buffer **B-8**, both EDTA-free.

Analysis of Denatured Protein Samples (SDS-PAGE)

Separating gel of 8% polyacrylamide was first poured in separating buffer **B-9**, APS, SDS and TEMED in mini-PROTEAN® glass plates (83 × 73 × 0.75 mm). Stacking gel of 5% polyacrylamide was poured afterwards similarly except in stacking buffer **B-10**. Proteins for analysis were incubated in loading buffer **B-11** at 95ºC for 5 min, and centrifuged for 5 min. The supernatant was recovered for electrophoresis (250 V, 40 min) in running buffer **B-12**. Protein bands were stained with Coomassie Brilliant Blue buffer **B-13**, following destaining in destaining buffer **B-14** prior to gel imaging.

Cloning of Klenow Exo⁻

Plasmid for Klenow Exo⁻ Expression

A Klenow Exo⁻ polymerase gene fragment (GenBank Accession Number V00317.1), with D355A, E357A substitutions that abolishes bidirectional exonuclease activities, was synthesized and cloned directionally into pET-28a(+) via restriction sites *EcoRI* and *NcoI* (Twist BioScience Expression Genes Service, San Francisco, CA, USA). The resulting polymerase contains in-frame His_6 sequences at both *N*- and *C*-termini. The resulting expression vector, **pET-28a(+)-KlenowExo⁻** (**Figure S65**).

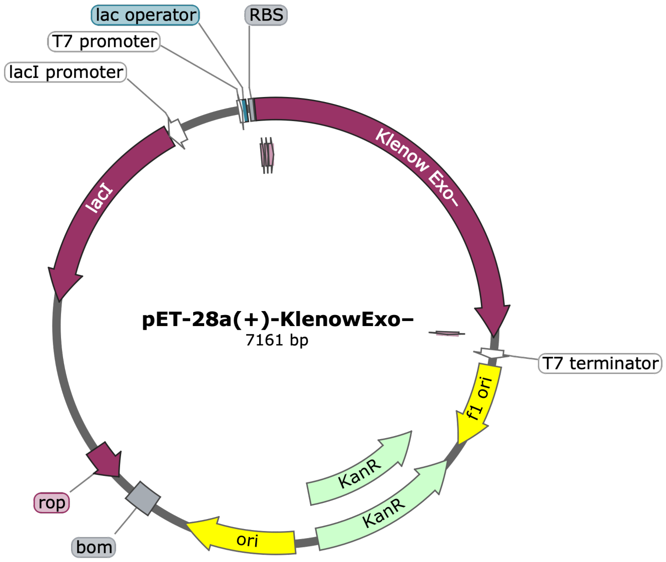

Figure S65. Plasmid Map of **pET-28(+)-KlenowExo^{-}**. The plasmid contains a kanamycin resistance (*KanR*) gene for antimicrobial selection, and is inducible via the *lac* operon. The gene of interest containing Klenow Exo^{-} is flanked by polyhistidine sequences for downstream purification by immobilized metal affinity chromatography (IMAC).

A FASTA representation of the plasmid is shown below. The open reading frame encoding for the polymerase is underlined.

```
> pET-28a(+)-KlenowExo- (7671 bp)
GTGATTTCTTATGACAACTACGTCACCATCCTTGATGAAGAAACACTGAAAGCGTGGATTGCGAAGCTG
GAAAAAGCGCCGGTATTTGCATTTGCTACCGCAACCGACAGCCTTGATAACATCTCTGCTAACCTGGTC
GGGCTTTCTTTTGCTATCGAGCCAGGCGTAGCGGCATATATTCCGGTTGCTCATGATTATCTTGATGCG
CCCGATCAAATCTCTCGCGAGCGTGCACTCGAGTTGCTAAAACCGCTGCTGGAAGATGAAAAGGCGCTG
AAGGTCGGGCAAAACCTGAAATACGATCGCGGTATTCTGGCGAACTACGGCATTGAACTGCGTGGGATT
GCGTTTGATACCATGCTGGAGTCCTACATTCTCAATAGCGTTGCCGGGCGTCACGATATGGACAGCCTC
GCGGAACGTTGGTTGAAGCACAAAACCATCACTTTTGAAGAGATTGCTGGTAAAGGCAAAAATCAACTG
ACCTTTAACCAGATTGCCCTCGAAGAAGCCGGACGTTACGCCGCCGAAGATGCAGATGTCACCTTGCAG
TTGCATCTGAAAATGTGGCCGGATCTGCAAAAACACAAAGGGCCGTTGAACGTCTTCGAGAATATCGAA
ATGCCGCTGGTGCCGGTGCTTTCACGCATTGAACGTAACGGTGTGAAGATCGATCCGAAAGTGCTGCAC
AATCATTCTGAAGAGCTCACCCTTCGTCTGGCTGAGCTGGAAAAGAAAGCGCATGAAATTGCAGGTGAG
GAATTTAACCTTTCTTCCACCAAGCAGTTACAAACCATTCTCTTTGAAAAACAGGGCATTAAACCGCTG
AAGAAAACGCCGGGTGGCGCGCCGTCAACGTCGGAAGAGGTACTGGAAGAACTGGCGCTGGACTATCCG
TTGCCAAAAGTGATTCTGGAGTATCGTGGTCTGGCGAAGCTGAAATCGACCTACACCGACAAGCTGCCG
CTGATGATCAACCCGAAAACCGGGCGTGTGCATACCTCTTATCACCAGGCAGTAACTGCAACGGGACGT
TTATCGTCAACCGATCCTAACCTGCAAAACATTCCGGTGCGTAACGAAGAAGGTCGTCGTATCCGCCAG
GCGTTTATTGCGCCAGAGGATTATGTGATTGTCTCAGCGGACTACTCGCAGATTGAACTGCGCATTATG
GCGCATCTTTCGCGTGACAAAGGCTTGCTGACCGCATTCGCGGAAGGAAAAGATATCCACCGGGCAACG
GCGGCAGAAGTGTTTGGTTTGCCACTGGAAACCGTCACCAGCGAGCAACGCCGTAGCGCGAAAGCGATC
AACTTTGGTCTGATTTATGGCATGAGTGCTTTCGGTCTGGCGCGGCAATTGAACATTCCACGTAAAGAA
GCGCAGAAGTACATGGACCTTTACTTCGAACGCTACCCTGGCGTGCTGGAGTATATGGAACGCACCCGT
GCTCAGGCGAAAGAGCAGGGCTACGTTGAAACGCTGGACGGACGCCGTCTGTATCTGCCGGATATCAAA
TCCAGCAATGGTGCTCGTCGTGCAGCGGCTGAACGTGCAGCCATTAACGCGCCAATGCAGGGAACCGCC
GCCGACATTATCAAACGGGCGATGATTGCCGTTGATGCGTGGTTACAGGCTGAGCAACCGCGTGTACGT
ATGATCATGCAGGTACACGATGAACTGGTATTTGAAGTTCATAAAGATGATGTTGATGCCGTCGCGAAG
CAGATTCATCAACTGATGGAAAACTGTACCCGTCTGGATGTGCCGTTGCTGGTGGAAGTGGGGAGTGGC
GAAAACTGGGATCAGGCGCACGCGGCCGCACTCGAGCACCACCACCACCACCACTGAGATCCGGCTGCT
AACAAAGCCCGAAAGGAAGCTGAGTTGGCTGCTGCCACCGCTGAGCAATAACTAGCATAACCCCTTGGG
GCCTCTAAACGGGTCTTGAGGGGTTTTTTGCTGAAAGGAGGAACTATATCCGGATTGGCGAATGGGACG
CGCCCTGTAGCGGCGCATTAAGCGCGGCGGGTGTGGTGGTTACGCGCAGCGTGACCGCTACACTTGCCA
GCGCCCTAGCGCCCGCTCCTTTCGCTTTCTTCCCTTCCTTTCTCGCCACGTTCGCCGGCTTTCCCCGTC
AAGCTCTAAATCGGGGGCTCCCTTTAGGGTTCCGATTTAGTGCTTTACGGCACCTCGACCCCAAAAAAC
TTGATTAGGGTGATGGTTCACGTAGTGGGCCATCGCCCTGATAGACGGTTTTTCGCCCTTTGACGTTGG
AGTCCACGTTCTTTAATAGTGGACTCTTGTTCCAAACTGGAACAACACTCAACCCTATCTCGGTCTATT
CTTTTGATTTATAAGGGATTTTGCCGATTTCGGCCTATTGGTTAAAAAATGAGCTGATTTAACAAAAAT
TTAACGCGAATTTTAACAAAATATTAACGCTTACAATTTAGGTGGCACTTTTCGGGGAAATGTGCGCGG
AACCCCTATTTGTTTATTTTTCTAAATACATTCAAATATGTATCCGCTCATGAATTAATTCTTAGAAAA
ACTCATCGAGCATCAAATGAAACTGCAATTTATTCATATCAGGATTATCAATACCATATTTTTGAAAAA
GCCGTTTCTGTAATGAAGGAGAAAACTCACCGAGGCAGTTCCATAGGATGGCAAGATCCTGGTATCGGT
CTGCGATTCCGACTCGTCCAACATCAATACAACCTATTAATTTCCCCTCGTCAAAAATAAGGTTATCAA
GTGAGAAATCACCATGAGTGACGACTGAATCCGGTGAGAATGGCAAAAGTTTATGCATTTCTTTCCAGA
CTTGTTCAACAGGCCAGCCATTACGCTCGTCATCAAAATCACTCGCATCAACCAAACCGTTATTCATTC
```

GTGATTGCGCCTGAGCGAGACGAAATACGCGATCGCTGTTAAAAGGACAATTACAAACAGGAATCGAAT
GCAACCGGCGCAGGAACACTGCCAGCGCATCAACAATATTTTCACCTGAATCAGGATATTCTTCTAATA
CCTGGAATGCTGTTTTCCCGGGGATCGCAGTGGTGAGTAACCATGCATCATCAGGAGTACGGATAAAAT
GCTTGATGGTCGGAAGAGGCATAAATTCCGTCAGCCAGTTTAGTCTGACCATCTCATCTGTAACATCAT
TGGCAACGCTACCTTTGCCATGTTTCAGAAACAACTCTGGCGCATCGGGCTTCCCATACAATCGATAGA
TTGTCGCACCTGATTGCCCGACATTATCGCGAGCCCATTTATACCCATATAAATCAGCATCCATGTTGG
AATTTAATCGCGGCCTAGAGCAAGACGTTTCCCGTTGAATATGGCTCATAACACCCCTTGTATTACTGT
TTATGTAAGCAGACAGTTTTATTGTTCATGACCAAAATCCCTTAACGTGAGTTTTCGTTCCACTGAGCG
TCAGACCCCGTAGAAAAGATCAAAGGATCTTCTTGAGATCCTTTTTTTCTGCGCGTAATCTGCTGCTTG
CAAACAAAAAAACCACCGCTACCAGCGGTGGTTTGTTTGCCGGATCAAGAGCTACCAACTCTTTTTCCG
AAGGTAACTGGCTTCAGCAGAGCGCAGATACCAAATACTGTCCTTCTAGTGTAGCCGTAGTTAGGCCAC
CACTTCAAGAACTCTGTAGCACCGCCTACATACCTCGCTCTGCTAATCCTGTTACCAGTGGCTGCTGCC
AGTGGCGATAAGTCGTGTCTTACCGGGTTGGACTCAAGACGATAGTTACCGGATAAGGCGCAGCGGTCG
GGCTGAACGGGGGGTTCGTGCACACAGCCCAGCTTGGAGCGAACGACCTACACCGAACTGAGATACCTA
CAGCGTGAGCTATGAGAAAGCGCCACGCTTCCCGAAGGGAGAAAGGCGGACAGGTATCCGGTAAGCGGC
AGGGTCGGAACAGGAGAGCGCACGAGGGAGCTTCCAGGGGGAAACGCCTGGTATCTTTATAGTCCTGTC
GGGTTTCGCCACCTCTGACTTGAGCGTCGATTTTTGTGATGCTCGTCAGGGGGGCGGAGCCTATGGAAA
AACGCCAGCAACGCGGCCTTTTTACGGTTCCTGGCCTTTTGCTGGCCTTTTGCTCACATGTTCTTTCCT
GCGTTATCCCCTGATTCTGTGGATAACCGTATTACCGCCTTTGAGTGAGCTGATACCGCTCGCCGCAGC
CGAACGACCGAGCGCAGCGAGTCAGTGAGCGAGGAAGCGGAAGAGCGCCTGATGCGGTATTTTCTCCTT
ACGCATCTGTGCGGTATTTCACACCGCAATGGTGCACTCTCAGTACAATCTGCTCTGATGCCGCATAGT
TAAGCCAGTATACACTCCGCTATCGCTACGTGACTGGGTCATGGCTGCGCCCCGACACCCGCCAACACC
CGCTGACGCGCCCTGACGGGCTTGTCTGCTCCCGGCATCCGCTTACAGACAAGCTGTGACCGTCTCCGG
GAGCTGCATGTGTCAGAGGTTTTCACCGTCATCACCGAAACGCGCGAGGCAGCTGCGGTAAAGCTCATC
AGCGTGGTCGTGAAGCGATTCACAGATGTCTGCCTGTTCATCCGCGTCCAGCTCGTTGAGTTTCTCCAG
AAGCGTTAATGTCTGGCTTCTGATAAAGCGGGCCATGTTAAGGGCGGTTTTTTCCTGTTTGGTCACTGA
TGCCTCCGTGTAAGGGGGATTTCTGTTCATGGGGGTAATGATACCGATGAAACGAGAGAGGATGCTCAC
GATACGGGTTACTGATGATGAACATGCCCGGTTACTGGAACGTTGTGAGGGTAAACAACTGGCGGTATG
GATGCGGCGGGACCAGAGAAAAATCACTCAGGGTCAATGCCAGCGCTTCGTTAATACAGATGTAGGTGT
TCCACAGGGTAGCCAGCAGCATCCTGCGATGCAGATCCGGAACATAATGGTGCAGGGCGCTGACTTCCG
CGTTTCCAGACTTTACGAAACACGGAAACCGAAGACCATTCATGTTGTTGCTCAGGTCGCAGACGTTTT
GCAGCAGCAGTCGCTTCACGTTCGCTCGCGTATCGGTGATTCATTCTGCTAACCAGTAAGGCAACCCCG
CCAGCCTAGCCGGGTCCTCAACGACAGGAGCACGATCATGCGCACCCGTGGGGCCGCCATGCCGGCGAT
AATGGCCTGCTTCTCGCCGAAACGTTTGGTGGCGGGACCAGTGACGAAGGCTTGAGCGAGGGCGTGCAA
GATTCCGAATACCGCAAGCGACAGGCCGATCATCGTCGCGCTCCAGCGAAAGCGGTCCTCGCCGAAAAT
GACCCAGAGCGCTGCCGGCACCTGTCCTACGAGTTGCATGATAAAGAAGACAGTCATAAGTGCGGCGAC
GATAGTCATGCCCCGCGCCCACCGGAAGGAGCTGACTGGGTTGAAGGCTCTCAAGGGCATCGGTCGAGA
TCCCGGTGCCTAATGAGTGAGCTAACTTACATTAATTGCGTTGCGCTCACTGCCCGCTTTCCAGTCGGG
AAACCTGTCGTGCCAGCTGCATTAATGAATCGGCCAACGCGCGGGGAGAGGCGGTTTGCGTATTGGGCG
CCAGGGTGGTTTTTCTTTTCACCAGTGAGACGGGCAACAGCTGATTGCCCTTCACCGCCTGGCCCTGAG
AGAGTTGCAGCAAGCGGTCCACGCTGGTTTGCCCCAGCAGGCGAAAATCCTGTTTGATGGTGGTTAACG
GCGGGATATAACATGAGCTGTCTTCGGTATCGTCGTATCCCACTACCGAGATATCCGCACCAACGCGCA
GCCCGGACTCGGTAATGGCGCGCATTGCGCCCAGCGCCATCTGATCGTTGGCAACCAGCATCGCAGTGG
GAACGATGCCCTCATTCAGCATTTGCATGGTTTGTTGAAAACCGGACATGGCACTCCAGTCGCCTTCCC
GTTCCGCTATCGGCTGAATTTGATTGCGAGTGAGATATTTATGCCAGCCAGCCAGACGCAGACGCGCCG
AGACAGAACTTAATGGGCCCGCTAACAGCGCGATTTGCTGGTGACCCAATGCGACCAGATGCTCCACGC
CCAGTCGCGTACCGTCTTCATGGGAGAAAATAATACTGTTGATGGGTGTCTGGTCAGAGACATCAAGAA
ATAACGCCGGAACATTAGTGCAGGCAGCTTCCACAGCAATGGCATCCTGGTCATCCAGCGGATAGTTAA
TGATCAGCCCACTGACGCGTTGCGCGAGAAGATTGTGCACCGCCGCTTTACAGGCTTCGACGCCGCTTC

GTTCTACCATCGACACCACCACGCTGGCACCCAGTTGATCGGCGCGAGATTTAATCGCCGCGACAATTT
GCGACGGCGCGTGCAGGGCCAGACTGGAGGTGGCAACGCCAATCAGCAACGACTGTTTGCCCGCCAGTT
GTTGTGCCACGCGGTTGGGAATGTAATTCAGCTCCGCCATCGCCGCTTCCACTTTTTCCCGCGTTTTCG
CAGAAACGTGGCTGGCCTGGTTCACCACGCGGGAAACGGTCTGATAAGAGACACCGGCATACTCTGCGA
CATCGTATAACGTTACTGGTTTCACATTCACCACCCTGAATTGACTCTCTTCCGGGCGCTATCATGCCA
TACCGCGAAAGGTTTTGCGCCATTCGATGGTGTCCGGGATCTCGACGCTCTCCCTTATGCGACTCCTGC
ATTAGGAAGCAGCCCAGTAGTAGGTTGAGGCCGTTGAGCACCGCCGCCGCAAGGAATGGTGCATGCAAG
GAGATGGCGCCCAACAGTCCCCCGGCCACGGGGCCTGCCACCATACCCACGCCGAAACAAGCGCTCATG
AGCCCGAAGTGGCGAGCCCGATCTTCCCCATCGGTGATGTCGGCGATATAGGCGCCAGCAACCGCACCT
GTGGCGCCGGTGATGCCGGCCACGATGCGTCCGGCGTAGAGGATCGAGATCTCGATCCCGCGAAATTAA
TACGACTCACTATAGGGGAATTGTGAGCGGATAACAATTCCCCTCTAGAAATAATTTTGTTTAACTTTA
AGAAGGAGATATACCATGGGCAGCAGCCATCATCATCATCATCACAGCAGCGGCCTGGTGCCGCGCGGC
AGCCATATGGCTAGCATGACTGGTGGACAGCAAATGGGTCGCGGATCCGAATTC

Cloning, Expression, Purification, and Characterization of Klenow Exo⁻ Polymerase

Chemically competent *E. coli* strain BL21 was a generous gift from Kathryn Lyle, Paris Salamon, Naniko Mirotadze, Dr. Katherine Ryan (Department of Chemistry, UBC), which was used to transform BL21 with plasmid **pET-28a(+)-KlenowExo⁻** in 1X LB supplemented with kanamycin sulfate (30 µg/mL final).

For protein production, a flask of transformed BL21 (300 mL) was grown in 37ºC in 5% CO_2 atmosphere. Following induction with IPTG (500 µM final added at OD_{600} = 0.8), the cells were supplemented with PMSF (100 µM final), and sonicated to generate cell lysates for immobilized metal affinity chromatography (IMAC) using a Ni Sepharose High Performance column (Cytiva, 17526801) per manufacturer instructions. Briefly, 10–500 mM imidazole in elution buffer **B-15** was employed to isolate the polyhistidine-tagged Klenow Exo⁻ polymerase. Desired fractions were identified by SDS-PAGE, and then pooled into a cellulose dialysis membrane (Millipore Sigma D9777-100FT) parcel for dialysis in buffer **B-16** (100 mM TRIS-HCl, 400 µM EDTA, pH 7.4) at 4ºC for 16 h, yielding pure in-house Klenow Exo⁻ polymerase (1.6 mg, 4.5 mL, 362 µg/mL, 73 kDa, 22 nmol, 5.0 µM, quantified by Pierce™ Bradford Protein Assay Kit, ThermoFisher 23200) for subsequent biological activity validation. In a standard assay of final volume 10 µL, 0.5 µM **ON-5**, 0.55 µM **ON-4**, 1X CutSmart Buffer, 10 µM dNTP, 2.5 ng/µL in-house Klenow Exo⁻ was incubated in 37 ºC for 30 min obtain full length primer extension product as demonstrated by denaturing PAGE (**Figure S66**, 15%, 400 V, 23 min).

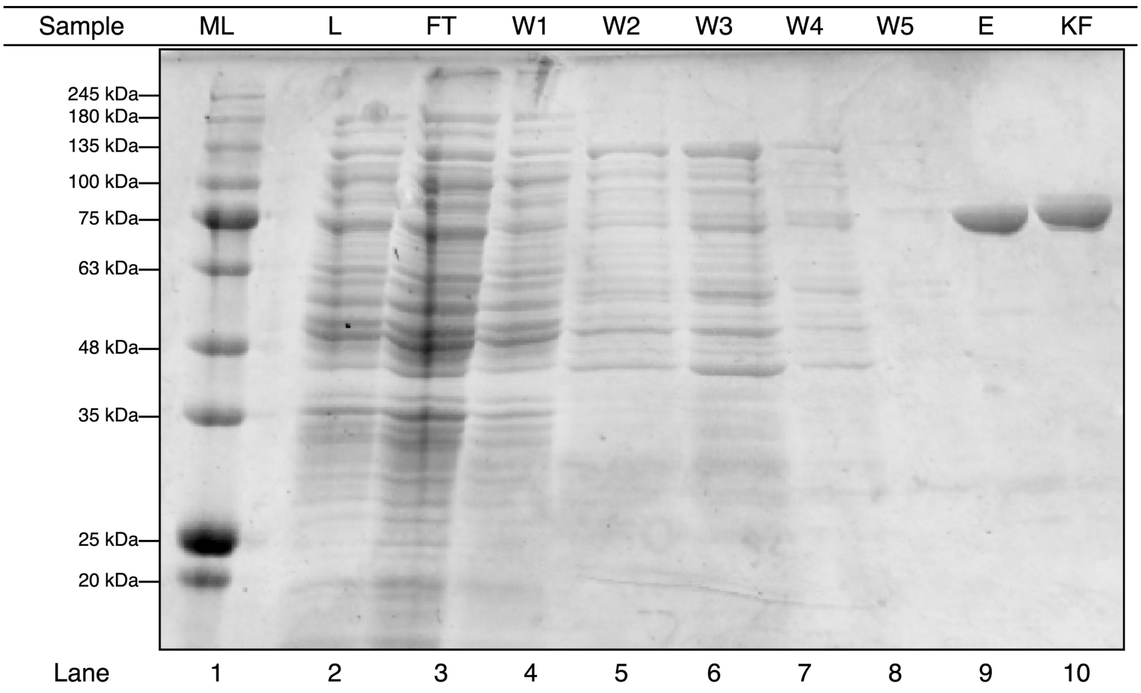

Figure S66. Purification of Klenow Exo⁻ polymerase following IMAC. Aliquots of Klenow Exo⁻ polymerase at different stages of purification were separated by SDS-PAGE following Coomassie Brilliant Blue staining. Lane 1, Molecular ladder (ML); Lane 2, Lysate (L); Lane 3, Sample flow through (FT); Lane 4, Wash 1 (W1) with 10 mM imidazole in **B-15**; Lane 5, Wash 2 (W2); Lane 6, Wash 3 (W3) with 50 mM imidazole in **B-15**; Lane 7, Wash 4 (W4); Lane 8, Wash 5 (W5); Lane 9, Elution (E) with 500 mM imidazole in **B-15**; Lane 10, Klenow Exo⁻ Fragment (KF) standard (73 kDa).

Klenow Exo⁻ Polymerase Activity Assays

Routine Primer Extension

A 2.5X annealing mix containing NEBuffer 2 (final 1.0 X), 5′-Cy5-labeled primer **ON-5** (final 0.5 μM), and appropriate template (final 1.1 μM, 2.2 eq.) with sufficient ddH_2O was heated to 95ºC for 5 minutes, then allowed to cool at –2.0ºC min^{-1} from 95ºC to 35ºC in a thermocycler. After annealing, yeast inorganic pyrophosphatase (YIPP, final 5 U/μL), Klenow Fragment (3′→5′ Exo⁻ , final 0.75 U/μL) and DTT (final 2.5 mM added in addition to NEBuffer 2, total 3.5 mM) was added to 2.5X annealing mix form a 2.0X master mix. Separately, 2X nucleotide mixes were prepared in accordance (see below) such that the reaction commenced when the 2.0X master mix was introduced to the corresponding 2.0X nucleotide mixes in 1:1 volume ratio to a final volume of 10 μL per reaction, following incubation at 37ºC for 9–60 min. Typically, 10% of the reaction crude was heated for 95ºC in **B-5** for 5 min prior to denaturing PAGE analysis (15%, 400 V, 23 min), following fluorescent-mode gel imaging.

Standardized Primer Extensions

For the purpose of standardizing a model reaction condition, the following parameters were used. [Primer **ON-5**]$_{Final}$ = 500 μM, [Template **ON-1–4**]$_{Final}$ = 1.1 μM, reaction buffer = 1X NEBuffer 2.0, incubation temperature = 37 ºC, incubation time = 60 min, information on [dNTP]$_{Final}$ are available in **Figure S67**–**Figure S71**.

Source	-	NEB	In-house				
[DNA Pol] (U/μL)	-	1.25	-				
[DNA Pol] (ng/μL)	-	-	2.5	5	10	20	30
[dNTP] (μM, each)	-	10	10	10	10	10	10

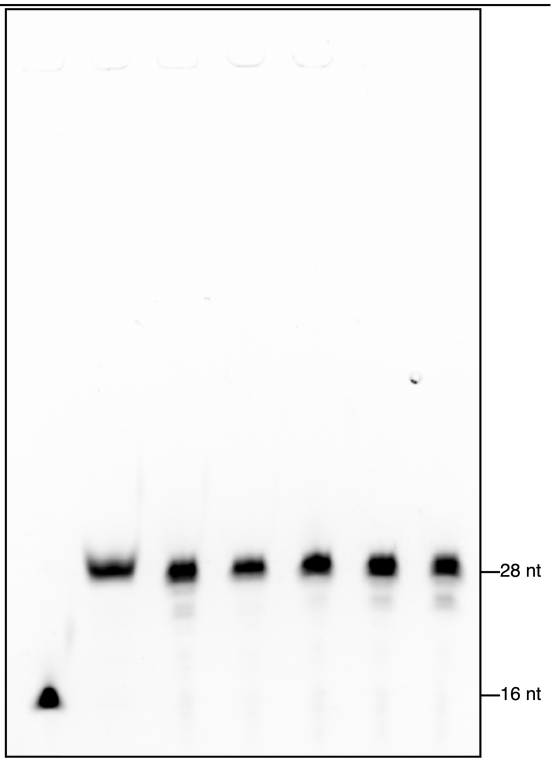

Lane 1 2 3 4 5 6 7

Figure S67. Preliminary functional validation of in-house Klenow Exo⁻ polymerase against commercial (New England Biolabs NEB) sample with only canonical nucleotide triphosphates.

A

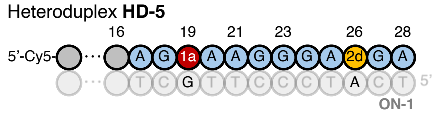

B

[dGTP]	*µ*M	-	-	10	10	10	10	10	10	10	10
[dATP]	*µ*M	-	10	10	10	10	10	10	10	10	10
C Analog		-	-	-	C	-	C	**1a**	C	**1a**	**1a**
[C Analog]	*µ*M	-	-	-	8	-	8	8	8	8	40
T Analog		-	-	-	-	T	T	T	**2d**	**2d**	**2d**
[T Analog]	*µ*M	-	-	-	-	8	8	8	8	8	40

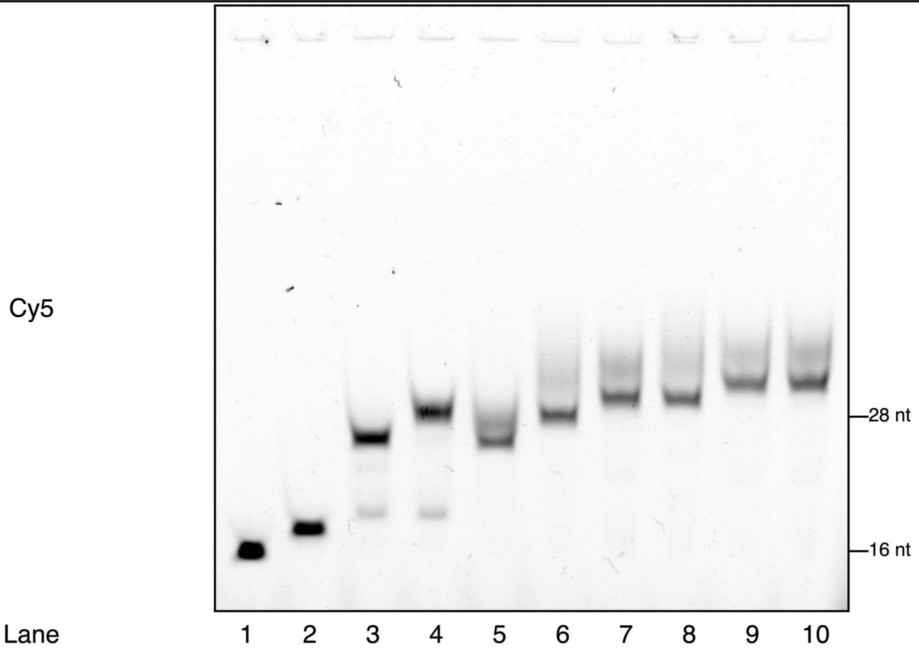

Figure S68. Primer extension using template **ON-1**, 5′-Cy5-labeled primer **ON-5** (16 nt), and modified nucleotides **1a** and **2d**. (**A**) The expected heteroduplex **HD-5** in the case where both **1a** and **2d** were used in lieu of dCTP (C) or dTTP (T) respectively. Grey circles represent commercially obtained oligonucleotides, blue, red, and yellow circles respectively represent canonical dNTPs, **1a**, and **2d** added by the polymerase. (**B**) PEx products were resolved by denaturing PAGE with different degrees of modification. Final dNTP concentrations for each primer extension are indicated in the respective lanes.

A

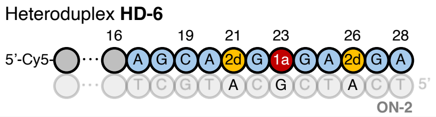

B

[dGTP]	*μ*M	-	-	10	10	10	10	10	10	10	10
[dATP]	*μ*M	-	10	10	10	10	10	10	10	10	10
C Analog		-	-	-	C	-	C	**1a**	C	**1a**	**1a**
[C Analog]	*μ*M	-	-	-	8	-	8	8	8	8	40
T Analog		-	-	-	-	T	T	T	**2d**	**2d**	**2d**
[T Analog]	*μ*M	-	-	-	-	8	8	8	8	8	40

Cy5

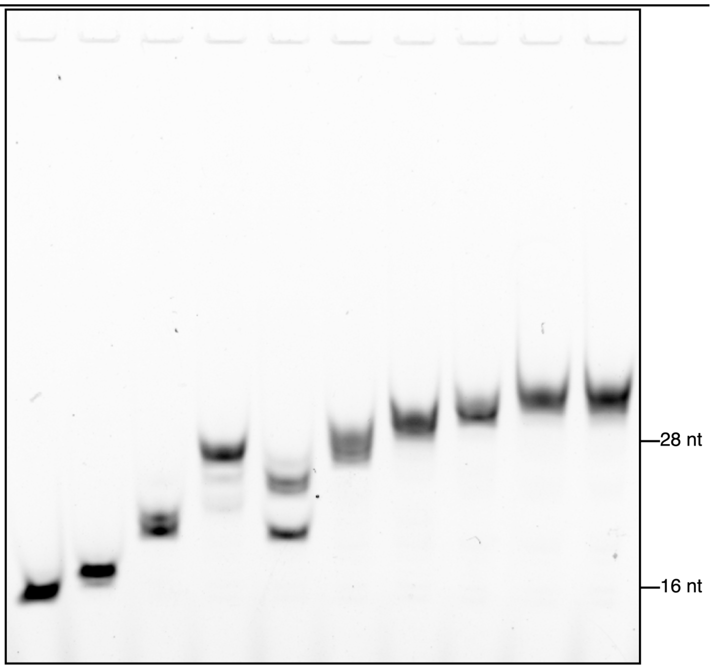

Lane 1 2 3 4 5 6 7 8 9 10

Figure S69. Primer extension using template **ON-2**, 5′-Cy5-labeled primer **ON-5** (16 nt), and modified nucleotides **1a** and **2d**. (**A**) The expected heteroduplex **HD-6** in the case where both **1a** and **2d** were used in lieu of dCTP (C) or dTTP (T) respectively. Grey circles represent commercially obtained oligonucleotides, blue, red, and yellow circles respectively represent canonical dNTPs, **1a**, and **2d** added by the polymerase. (**B**) PEx products were resolved by denaturing PAGE with different degrees of modification. Final dNTP concentrations for each primer extension are indicated in the respective lanes.

A

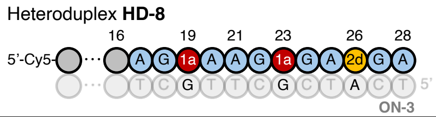

B

		1	2	3	4	5	6	7	8	9	10
[dGTP]	μM	-	-	10	10	10	10	10	10	10	10
[dATP]	μM	-	10	10	10	10	10	10	10	10	10
C Analog		-	-	-	C	-	C	**1a**	C	**1a**	**1a**
[C Analog]	μM	-	-	-	8	-	8	8	8	8	40
T Analog		-	-	-	-	T	T	T	**2d**	**2d**	**2d**
[T Analog]	μM	-	-	-	-	8	8	8	8	8	40

Cy5

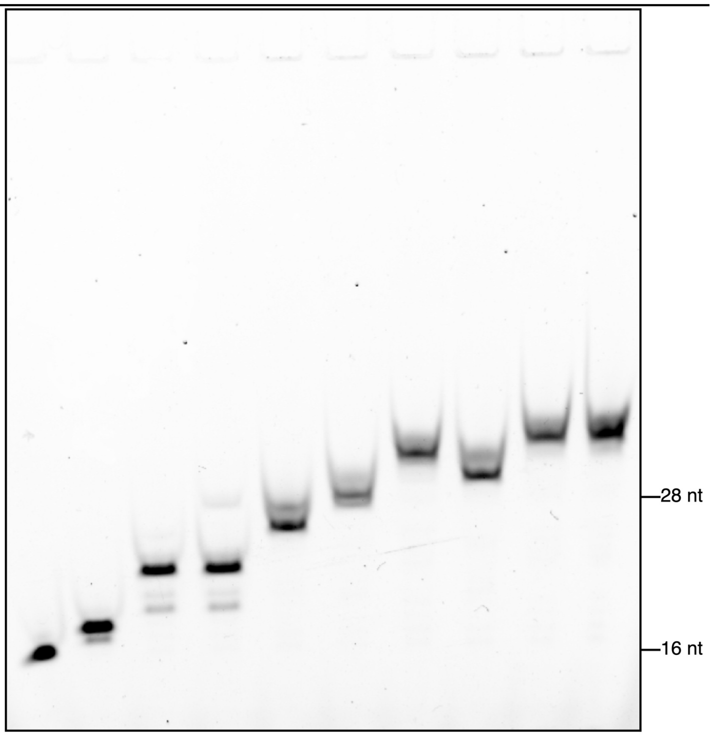

Lane 1 2 3 4 5 6 7 8 9 10

Figure S70. Primer extension using template **ON-3**, 5′-Cy5-labeled primer **ON-5** (16 nt), and modified nucleotides **1a** and **2d**. (**A**) The expected heteroduplex **HD-8** in the case where both **1a** and **2d** were used in lieu of dCTP (C) or dTTP (T) respectively. Grey circles represent commercially obtained oligonucleotides, blue, red, and yellow circles respectively represent canonical dNTPs, **1a**, and **2d** added by the polymerase. (**B**) PEx products were resolved by denaturing PAGE with different degrees of modification. Final dNTP concentrations for each primer extension are indicated in the respective lanes.

A

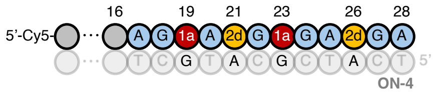

B

[dGTP]	μM	-	-	10	10	10	10	10	10	10	10
[dATP]	μM	-	10	10	10	10	10	10	10	10	10
C Analog		-	-	-	C	-	C	**1a**	C	**1a**	**1a**
[C Analog]	μM	-	-	-	8	-	8	8	8	8	40
T Analog		-	-	-	-	T	T	T	**2d**	**2d**	**2d**
[T Analog]	μM	-	-	-	-	8	8	8	8	8	40

Cy5

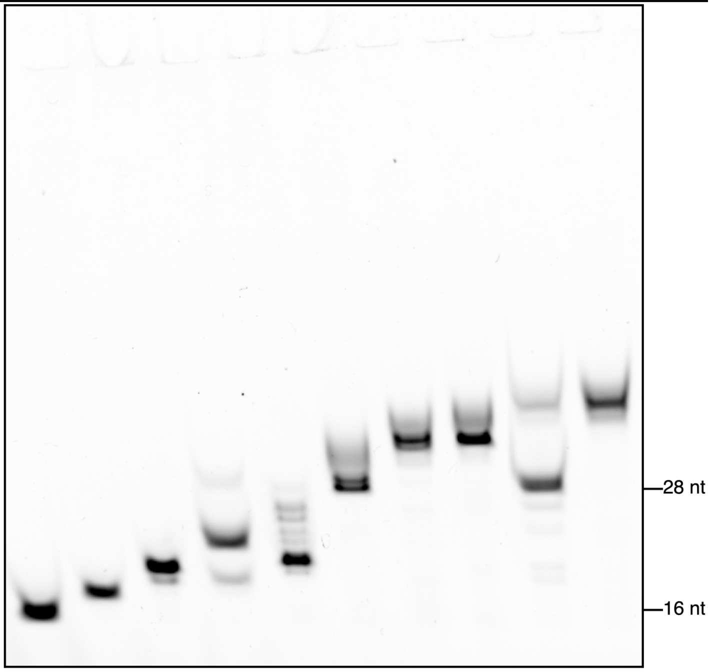

Lane	1	2	3	4	5	6	7	8	9	10

Figure S71. Primer extension using template **ON-4**, 5′-Cy5-labeled primer **ON-5** (16 nt), and modified nucleotides **1a** and **2d**. (**A**) The expected heteroduplex **HD-9** in the case where both **1a** and **2d** were used in lieu of dCTP (C) or dTTP (T) respectively. Grey circles represent commercially obtained oligonucleotides, blue, red, and yellow circles respectively represent canonical dNTPs, **1a**, and **2d** added by the polymerase. (**B**) PEx products resolved by denaturing PAGE with different degrees of modification. Final dNTP concentrations for each primer extension are indicated in the respective lanes.

Primer Extension with up to 160 µM Modified Nucleotide Triphosphates

For the purpose of exploring the effects of high concentration of DOTA-modified nucleotide triphosphates (up to 160 µM), the parameters in **Standardized Primer Extensions** were used. Information on $[dNTP]_{Final}$ are available in **Figure S72**.

Template		**ON-1**	**ON-1**	**ON-1**	**ON-4**	**ON-1**	**ON-1**	**ON-1**	**ON-2**	**ON-4**	**ON-3**	**ON-4**	**ON-4**	**ON-1**
[dGTP]	µM	-	10											
[dATP]	µM	-	10											
[dCTP]	µM	-	5	5	5	-	-	-	-	-	-	-	-	-
[dTTP]	µM	-	5	-	-	5	-	-	-	5	-	-	-	-
**[1b]**	µM	-	-	-	-	40	40	160	40	40	40	40	160	-
**[2d]**	µM	-	-	40	40	-	40	160	40	-	40	40	160	-
Number of modified nucleotides		-	-	1	2	1	2	2	3	2	3	4	4	-

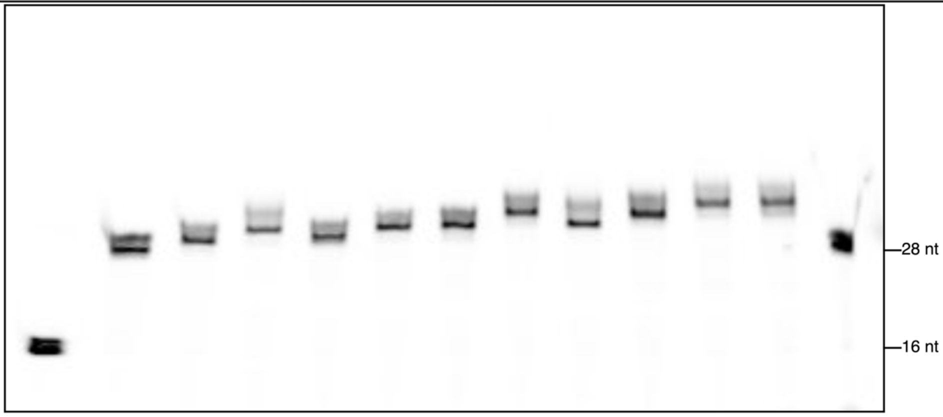

Lane	1	2	3	4	5	6	7	8	9	10	11	12	13

Figure S72. Primer extension using templates **ON-1–4**, 5′-Cy5-labeled primer **ON-5** (16 nt), and modified nucleotides **1b** and **2d**. PEx products were resolved by denaturing PAGE with different degrees of modification. Final dNTP concentrations for each primer extension are indicated in the respective lanes.

Time-Course Analysis of Primer Extension Using Modified Nucleotide Triphosphates

For the purpose of exploring whether higher concentration of DOTA-modified nucleotide triphosphates, the parameters in **Standardized Primer Extensions** were used, except aliquots of reaction crudes were removed at 9–60 min. Information on $[dNTP]_{Final}$ are available in **Figure S73**.

Template		-	**ON-1**	**ON-1**			**ON-4**			**ON-4**			-	**ON-1**
[dGTP]	*μ*M	-	10											
[dATP]	*μ*M	-	10											
[dCTP]	*μ*M	-	5	-			-			-			-	5
[dTTP]	*μ*M	-	5	-			-			-			-	5
**[1b]**	*μ*M	-	-	40			40			160			-	-
**[2d]**	*μ*M	-	-	40			40			160			-	-
Number of modified nucleotides		-	-	2			4			4			-	-
Incubation Time (min)		-	60	9	30	60	9	30	60	9	30	60	-	60

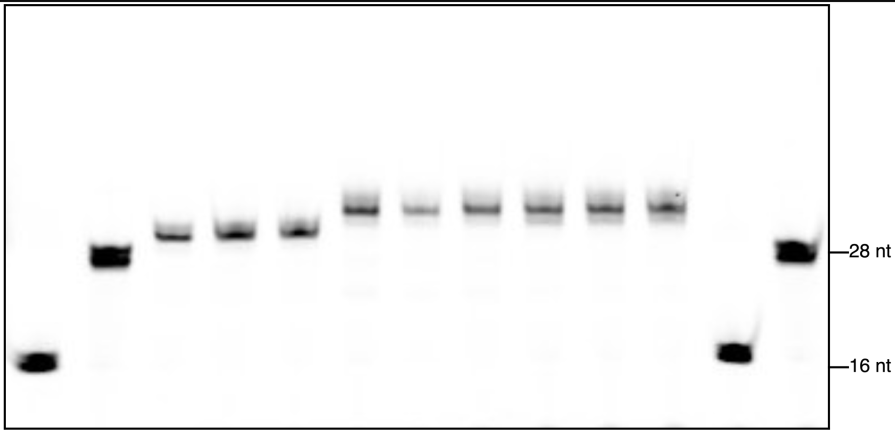

Lane	1	2	3	4	5	6	7	8	9	10	11	12	13

Figure S73. Primer extension using templates **ON-1** or **ON-4**, 5′-Cy5-labeled primer **ON-5** (16 nt), and modified nucleotides **1b** and **2d**. Aliquots of PEx products were removed at time points 9, 30 or 60 min and resolved by denaturing PAGE with different degrees of modification. Final dNTP concentrations for each primer extension are indicated in the respective lanes.

Lanes 3–8 show that when metalated triphosphate concentrations of $[\mathbf{1b}]_{Final} = [\mathbf{2d}]_{Final}$ = 40 μM were used, primer extensions were fully completed within 9 minutes for both templates **ON-1** and **ON-4**. At higher concentrations of $[\mathbf{1b}]_{Final} = [\mathbf{2d}]_{Final}$ = 160 μM (lanes 9–11), the extension with template **ON-4** also reached completion.

Primer Extension with XadYTP-PEG$_n$-DOTA series **19–24**

Appreciating the success of DOTA-modified nucleotide triphosphates with C_6 linker **1** and **2**, the PEG linker series **19–21** (**Figure S74**) and **22–24** (**Figure S75**) were synthesized and validated by primer extension with templates **ON-10–11** and primer **ON-5**. Unless otherwise stated, parameters in **Standardized Primer Extensions** were used, and [modified nucleotides **19–24**]$_{Final}$ = 40 µM.

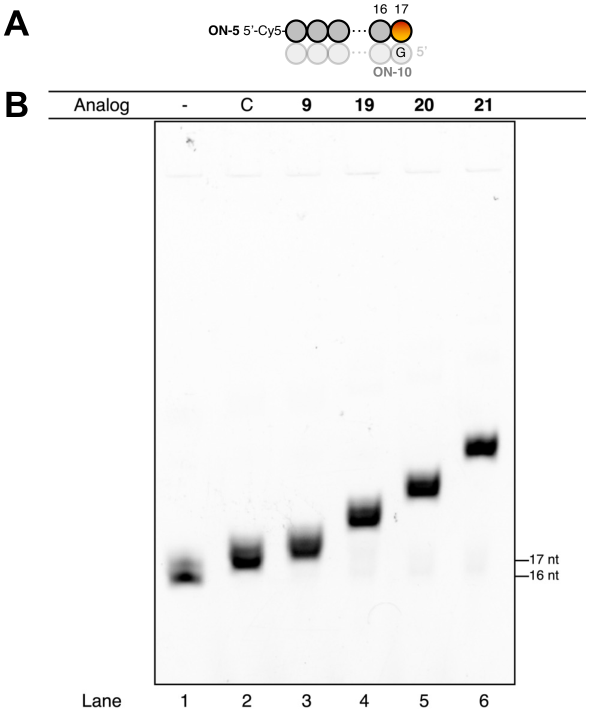

Figure S74. Primer extension using template **ON-10**, 5′-Cy5-labeled primer **ON-5** (16 nt), commercially available nucleotide triphosphates dCTP (C), 5-propargylamino-dCTP **9**, and modified counterparts **19–21**. (**A**) The expected heteroduplex product after primer extension. Grey circles represent commercially obtained oligonucleotides, blue and red-yellow circles respectively represent canonical dNTPs and the nucleobase to be added to position 17. (**B**) The PEx products were resolved by denaturing PAGE.

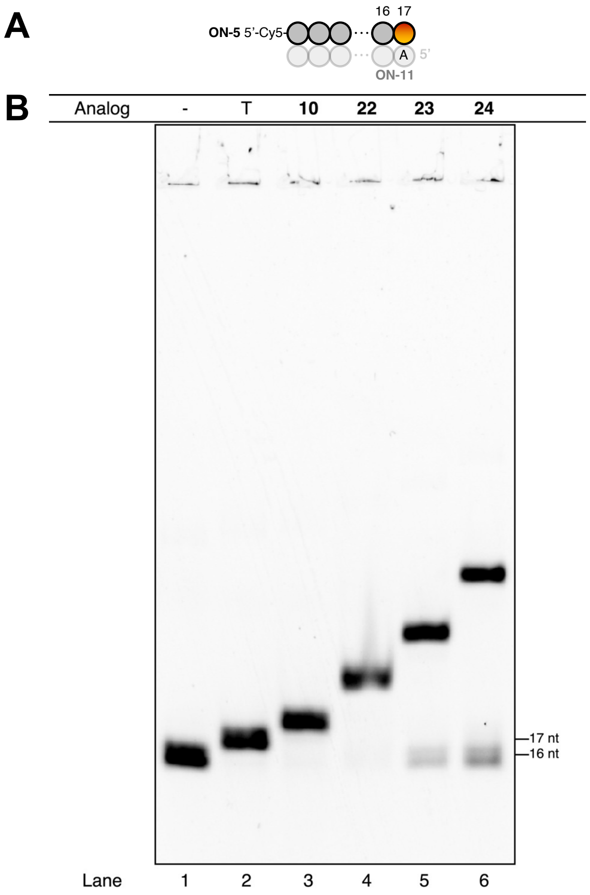

Figure S75. Primer extension using template **ON-11**, 5′-Cy5-labeled primer **ON-5** (16 nt), commercially available nucleotide triphosphates dCTP (C), 5-aminoallyl-dUTP **10**, and modified counterparts **22–24**. (**A**) The expected heteroduplex product after primer extension. Grey circles represent commercially obtained oligonucleotides, blue and red-yellow circles respectively represent canonical dNTPs and the nucleobase to be added to position 17. (**B**) The PEx products were resolved by denaturing PAGE.

Deleterious Effects of Superstoichiometric Metal Trication on Primer Extension

Standards for generating non-radioactive metal complexes typically involve adding superstoichiometric amounts of metals to the chelator of choice to ensure complete conversion to the metal complex. However, we report that this approach is not advisable for this process (**Figure S76**). Exceptionally for this experiment, 3.0 eq. MCl_3 (M = Y or Lu) was used to prepare complexed triphosphates **1b** and **2d** for primer extension with template **ON-1** and primer **ON-5**. Unless otherwise stated, parameters in **Standardized Primer Extensions** were used.

A

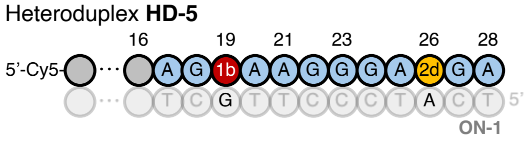

B

		1	2	3	4	5	6	7	8	9	10	11	12	13	14
[dGTP]	μM	-	10												
[dATP]	μM	-	10												
[dCTP]	μM	-	-	5	-	5	5	5	5	5	-	-	5	-	-
[dTTP]	μM	-	-	-	5	5	5	5	5	-	5	-	-	5	-
[YCl_3]	μM	-	-	-	-	-	-	40	40	-	-	-	-	-	-
[$LuCl_3$]	μM	-	-	-	-	-	40	-	40	-	-	-	-	-	-
**[1]**	μM	-	-	-	-	-	-	-	-	-	40	40	-	-	-
**[2]**	μM	-	-	-	-	-	-	-	-	40	-	40	-	-	-
**[1b]**	μM	-	-	-	-	-	-	-	-	-	-	-	-	40	40
**[2d]**	μM	-	-	-	-	-	-	-	-	-	-	-	40	-	40

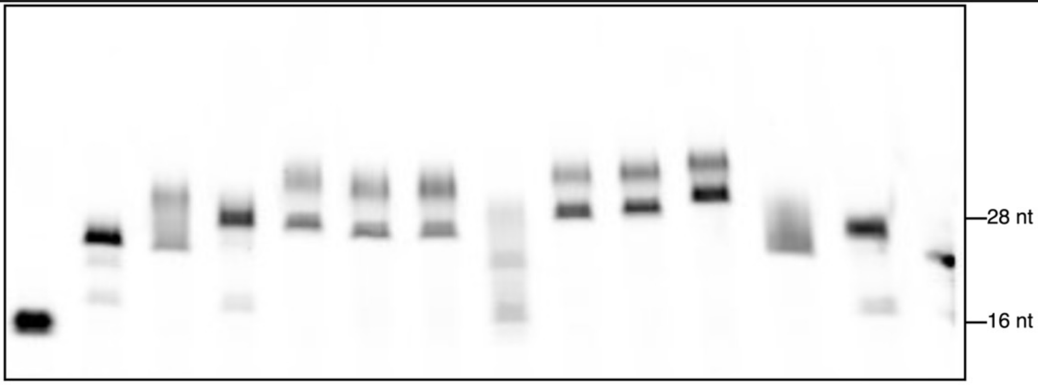

Lane 1 2 3 4 5 6 7 8 9 10 11 12 13 14

Figure S76. Primer extension using template **ON-1**, 5′-Cy5-labeled primer **ON-5** (16 nt), modified nucleotide triphosphates **1**, **2**, **1b**, **2d**. (**A**) The expected heteroduplex **HD-5** in the case where both **1b** and **2d** were used in lieu of dCTP (C) or dTTP (T) respectively. Grey circles represent commercially obtained oligonucleotides, blue, red, and yellow circles respectively represent canonical dNTPs, **1b**, and **2d** added by the polymerase. (**B**) The PEx products were resolved by denaturing PAGE. Final MCl_3 and dNTP concentrations for each primer extension are indicated in the respective lanes.

Appreciating that lane 5 as the expected product with canonical bases, lanes 6–8 simulated the effects of excess Y^{3+} and Lu^{3+} inhibiting primer extension. Notwithstanding when either one of YCl_3 or $LuCl_3$ was added (lanes 6–7), the products obtained were shorter than that of lane 5. In the case when both YCl_3 and $LuCl_3$ were added (lane 8), early arrest of polymerase addition was apparent. Lanes 9–11 contains the expected products with modified nucleotides **1** and **2**.

Superstoichiometrically labelled **1b** and **2d** inhibited primer extension (lane 12–14) to the desired full-length products.

Radioactive Primer Extension

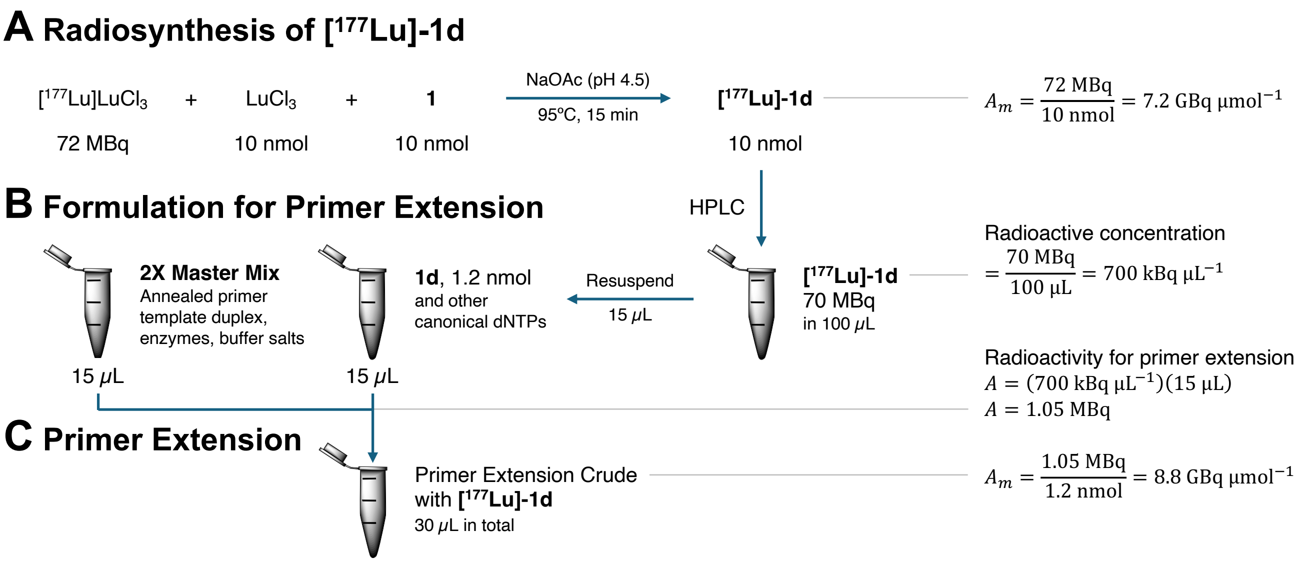

Figure S77. Typical radiolabeling of nucleotide triphosphates following primer extension with relevant radiochemical calculations, using **[^{177}Lu]-1d** as an example. (**A**) Radiosynthesis of **[^{177}Lu]-1d** with carrier added [^{177}Lu]$LuCl_3$, following HPLC purification. (**B**) Formulation of **[^{177}Lu]-1d** for primer extension by resuspending with dried **1d** (1.2 nmol) and canonical dNTPs with purified **[^{177}Lu]-1d** (15 µL at 700 kBq µL^{-1}). A 2X Master Mixture containing the annealed primer-template duplex, Klenow Exo^{-} polymerase, yeast inorganic pyrophosphatase (YIPP), and buffer salts was prepared ahead of time. (**C**) Primer Extension commenced by mixing 2X Master Mix and the formulated (radio)nucleotide triphosphate at 1:1 volume ratio, at a final volume of 30 µL.

The protocol was similar to the non-radioactive version discussed in **Routine Primer Extension**, with modifications specified in **Figure S77**. Briefly for a 30 µL reaction, a 2.5X annealing mix containing NEBuffer 2 (final 1.0 X), 5′-Cy5-labeled primer **ON-5** (final 0.5 µM), and template **ON-4** (final 1.1 µM, 2.2 eq.) with sufficient ddH_2O was heated to 95ºC for 5 minutes, then allowed to cool at 2.0 ºC min^{-1} from 95ºC to 35ºC in a thermocycler. After annealing, DTT (final 2.5 mM added in addition to NEBuffer 2, total 3.5 mM), yeast inorganic pyrophosphatase (YIPP, final 5 U/µL), and Klenow Fragment (3′→5′ Exo^{-}, final 0.75 U/µL) was added to 2.5X annealing mix form a 2.0X master mix (15 µL). Separately, freeze-dried nucleotide triphosphates (1.2 nmol non-radioactive pyrimidine triphosphate standard, 300 pmol each for dGTP and dATP, and 150 pmol for the remaining pyrimidine triphosphate) was resuspended in the carrier-added radioactive nucleotide triphosphate to 5 µL (2.0 X concentration). To balance radiation safety with detection sensitivity, primer extension was conducted with no more than 10 MBq total radioactivity. This allowed loading of approximately 400 kBq per lane, optimized for overnight autoradiography exposure. The reaction was initiated by combining the master mix (5 µL, 2 X) with the radioactive nucleotide triphosphates (15 µL, 2 X), in effect diluting to a final volume of 30 µL at 1X concentration, following incubation at 37ºC for 60 min. To ensure complete primer extension, decayed samples were analyzed by denaturing PAGE (15%, 400V, 20 min) and fluorescent-mode gel imaging.

Electrophoretic Mobility Shift Assay (EMSA)

Aliquots of extended primers (0.5 µM, 1 µL, 0.5 pmol) was introduced to DMSO (5 µL), choice of one of displacement oligonucleotide (**ON-7–9**, 50 µM, 1 µL, 50 pmol, 100 eq.), **B-1** (3 µL), and topped up with H_2O to total reaction volume of 10 µL. Strand displacement occurred in 45 ºC for 5 min. In the case of band retardation by streptavidin, streptavidin (2 mg/mL, 1 µL) was added to an aliquot of extended primers (0.5 µM, 1 µL, 0.5 pmol), **B-1** (3 µL), and H_2O topped up to 10 µL. All samples were analyzed by native PAGE (15%, 400 V, 15 min) in native gel running buffer **B-8**, following fluorescent-mode gel imaging and overnight autoradiographic exposure, where appropriate (**Figure S78**–**Figure S82**).

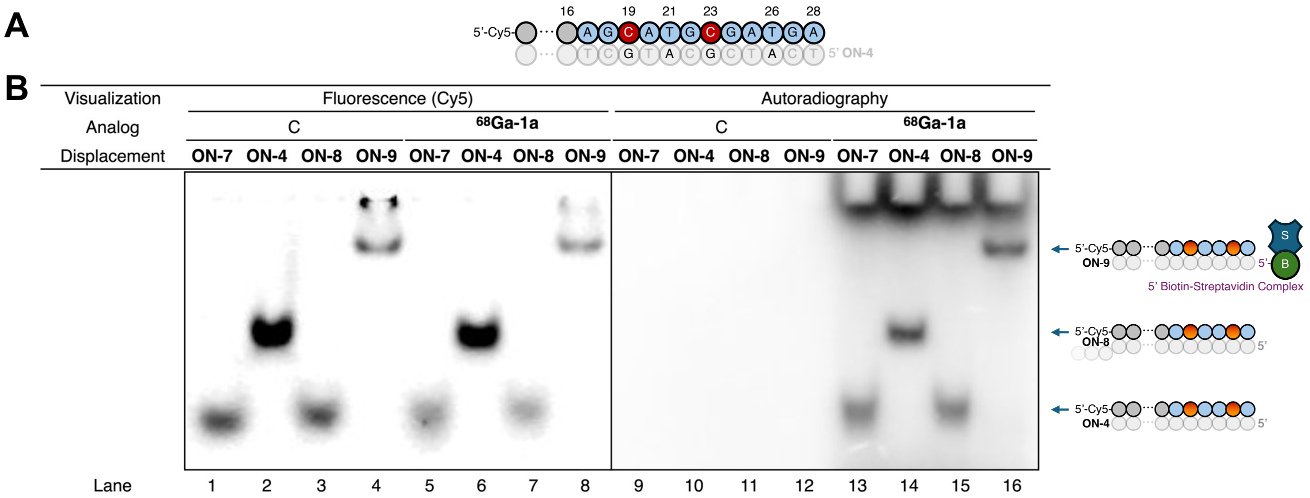

Figure S78. Primer extension using template **ON-4**, 5′-Cy5-labeled primer **ON-5** (16 nt), and modified nucleotide **^{68}Ga-1a**. (**A**) The expected heteroduplex in the case where **1a** was used in lieu of dCTP (C). Grey circles represent commercially obtained oligonucleotides, and red circles respectively represent **^{68}Ga-1a** added by the polymerase. (**B**) PEx products were strand-separated and rehybridized with **ON-4** or **ON-7–9**. The newly formed heteroduplexes were resolved by native PAGE.

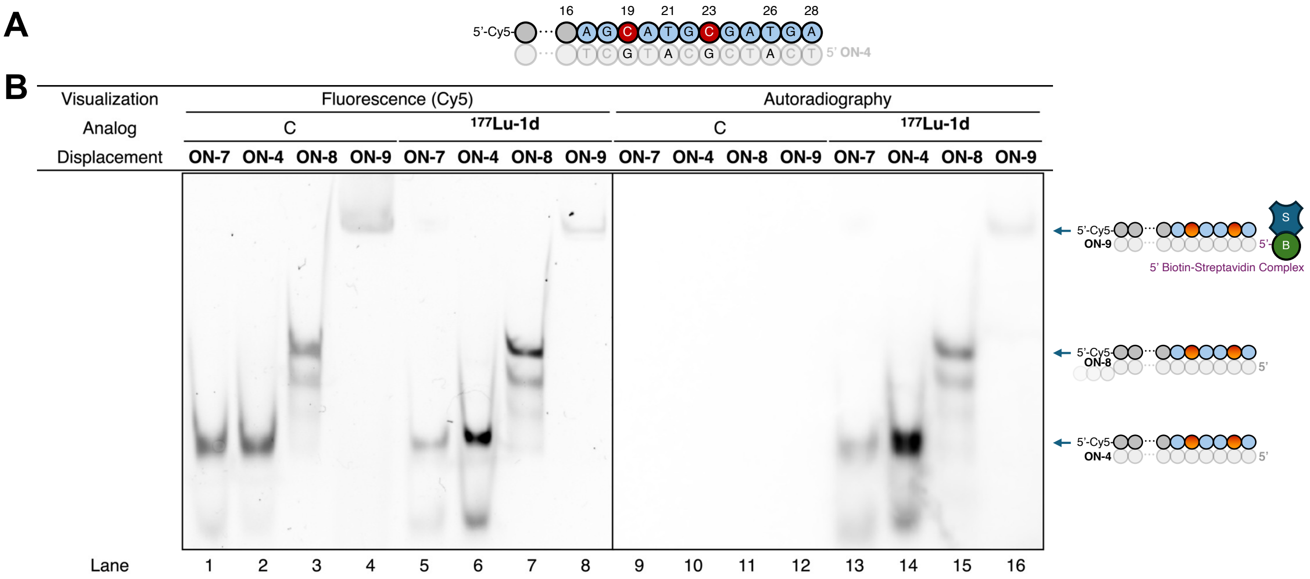

Figure S79. Primer extension using template **ON-4**, 5′-Cy5-labeled primer **ON-5** (16 nt), and modified nucleotide **^{177}Lu-1d**. (**A**) The expected heteroduplex in the case where **1d** was used in lieu of dCTP (C). Grey circles represent commercially obtained oligonucleotides, and red circles respectively represent **^{177}Lu-1d** added by the polymerase. (**B**) PEx products were strand-separated and rehybridized with **ON-4** or **ON-7–9**. The newly formed heteroduplexes were resolved by native PAGE.

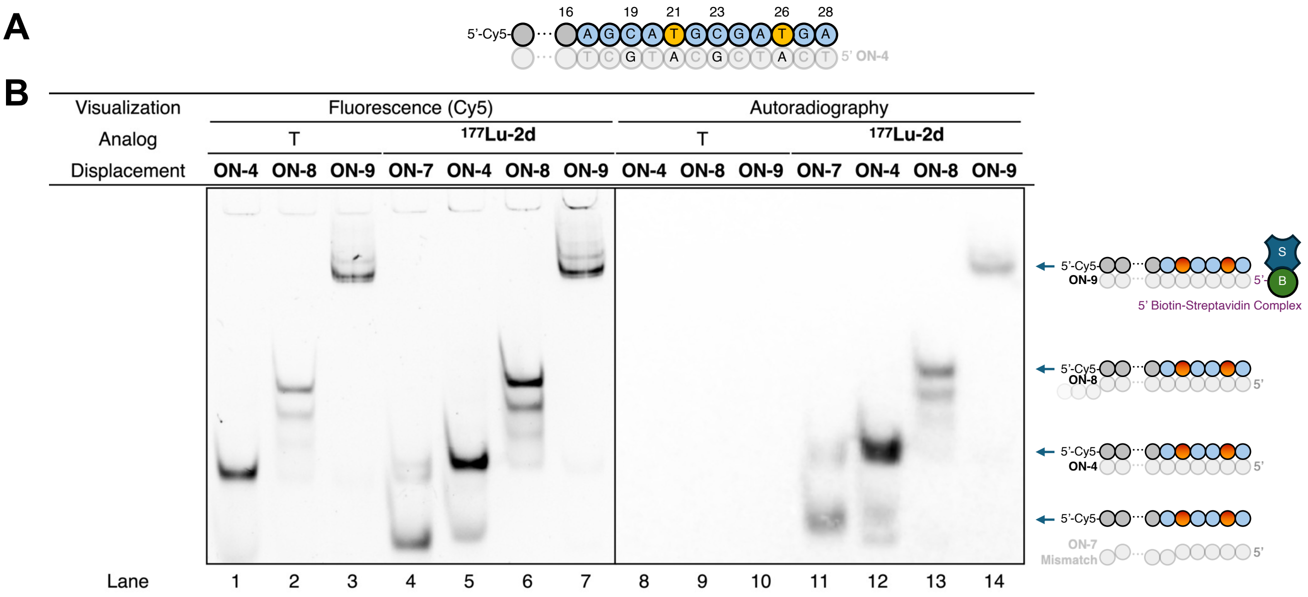

Figure S80. Primer extension using template **ON-4**, 5′-Cy5-labeled primer **ON-5** (16 nt), and modified nucleotide ^{177}**Lu-2d**. (**A**) The expected heteroduplex in the case where **2d** was used in lieu of dTTP (T). Grey circles represent commercially obtained oligonucleotides, and red circles respectively represent ^{177}**Lu-2d** added by the polymerase. (**B**) PEx products were strand-separated and rehybridized with **ON-4** or **ON-7–9**. The newly formed heteroduplexes were resolved by native PAGE.

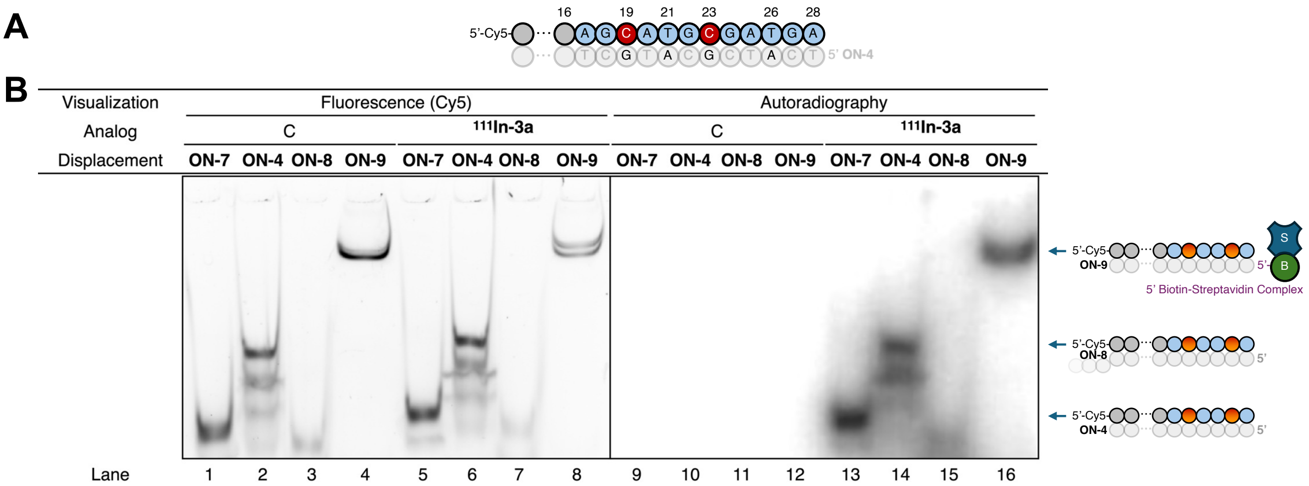

Figure S81. Primer extension using template **ON-4**, 5′-Cy5-labeled primer **ON-5** (16 nt), and modified nucleotide ^{111}**In-3a**. (**A**) The expected heteroduplex in the case where **3a** was used in lieu of dCTP (C). Grey circles represent commercially obtained oligonucleotides, and red circles respectively represent ^{111}**In-3a** added by the polymerase. (**B**) PEx products were strand-separated and rehybridized with **ON-4** or **ON-7–9**. The newly formed heteroduplexes were resolved by native PAGE.

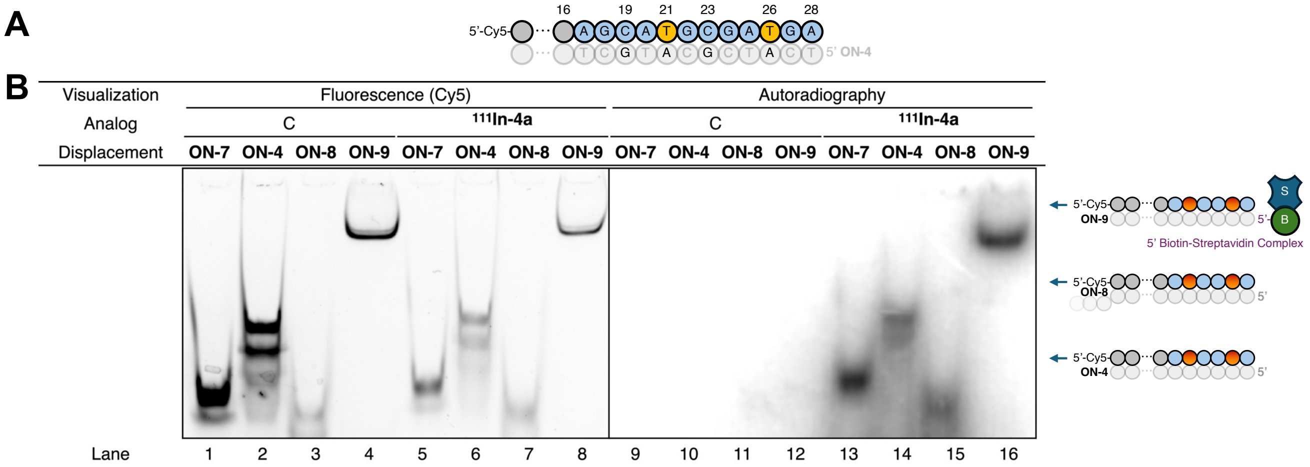

Figure S82. Primer extension using template **ON-4**, 5′-Cy5-labeled primer **ON-5** (16 nt), and modified nucleotide **^{111}In-4a**. (**A**) The expected heteroduplex in the case where **4a** was used in lieu of dTTP (T). Grey circles represent commercially obtained oligonucleotides, and red circles respectively represent **^{111}In-4a** added by the polymerase. (**B**) PEx products were strand-separated and rehybridized with **ON-4** or **ON-7–9**. The newly formed heteroduplexes were resolved by native PAGE.

Terminal Transferase 3′-ExtensionTerminal Deoxynucleotidyl Transferase (Radio)labeling

For preparation of **Figure 3**, to total reaction volume of 10 µL, primer **ON-6** (100 nM), Terminal Transferase Buffer (1X) **B-3** were subjected to 3′-extension by Terminal Transferase (0.5 U/µL, New England Biolabs M0315L) in one of dCTP (0.3–20 µM), **1d** (0.3–20 µM), or carrier added **^{177}Lu-1d** (0.3–20 µM, 0.2–7.2 GBq µmol^{-1}), or water as negative control, for 1 h at 37ºC. The extension products were separated by native PAGE (20%, 500 V, 30 min) electrophoresed in native PAGE buffer **B-8**. For similar studies involving other nucleotides (**Figure S83**–**Figure S86, Figure S88**, **Figure S89**), the supplied triphosphate was supplied to the same concentration range. For the purpose of data integrity, the un-cropped images used for **Figure S86** are also provided.

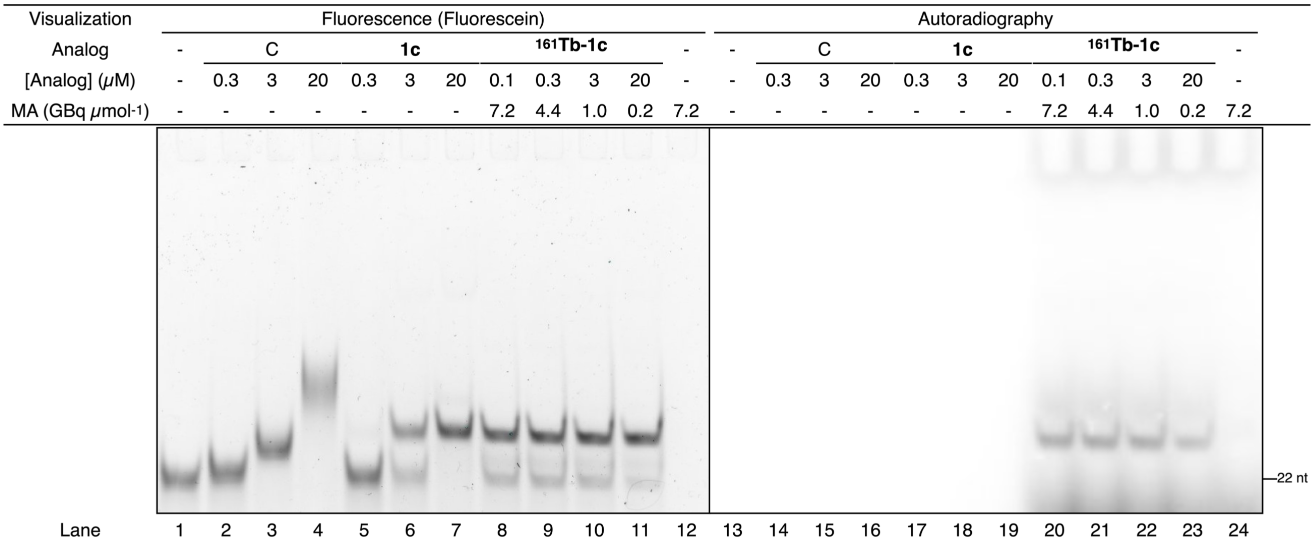

Figure S83. 3′-terminal transferase reaction using 5′-fluorescein-labeled primer **ON-6** (22 nt), and one of dCTP (C), and modified nucleotides **1c**, and **^{161}Tb-1c**. Concentrations and molar activities (for radioactive dNTPs) are indicated in each lane.

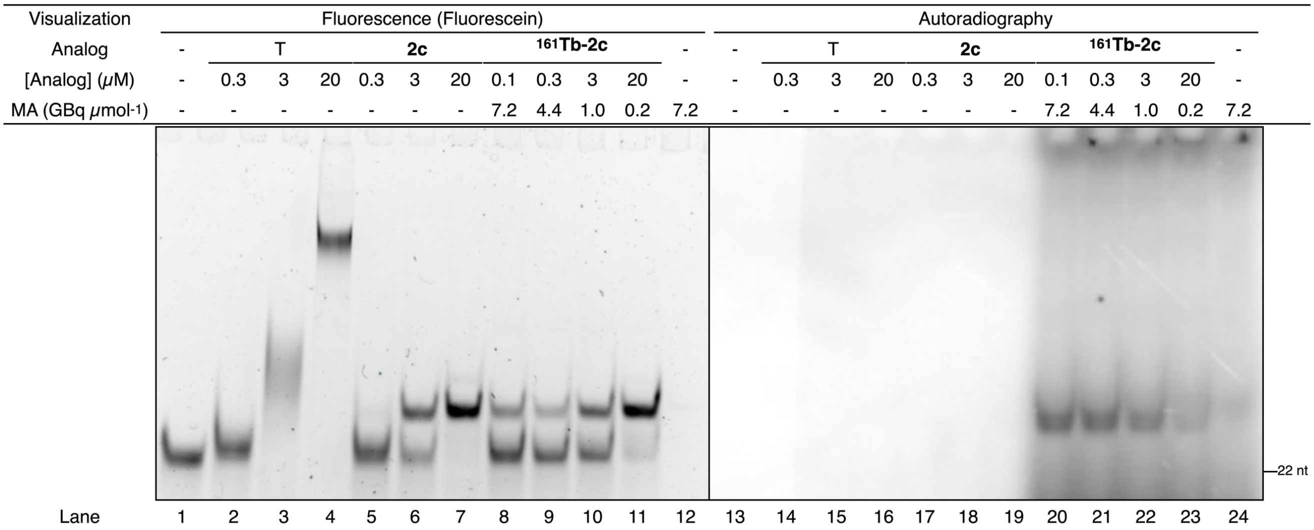

Figure S84. 3′-terminal transferase reaction using 5′-fluorescein-labeled primer **ON-6** (22 nt), and one of dTTP (T), and modified nucleotides **2c**, and **^{161}Tb-2c**. Concentrations and molar activities (for radioactive dNTPs) are indicated in each lane.

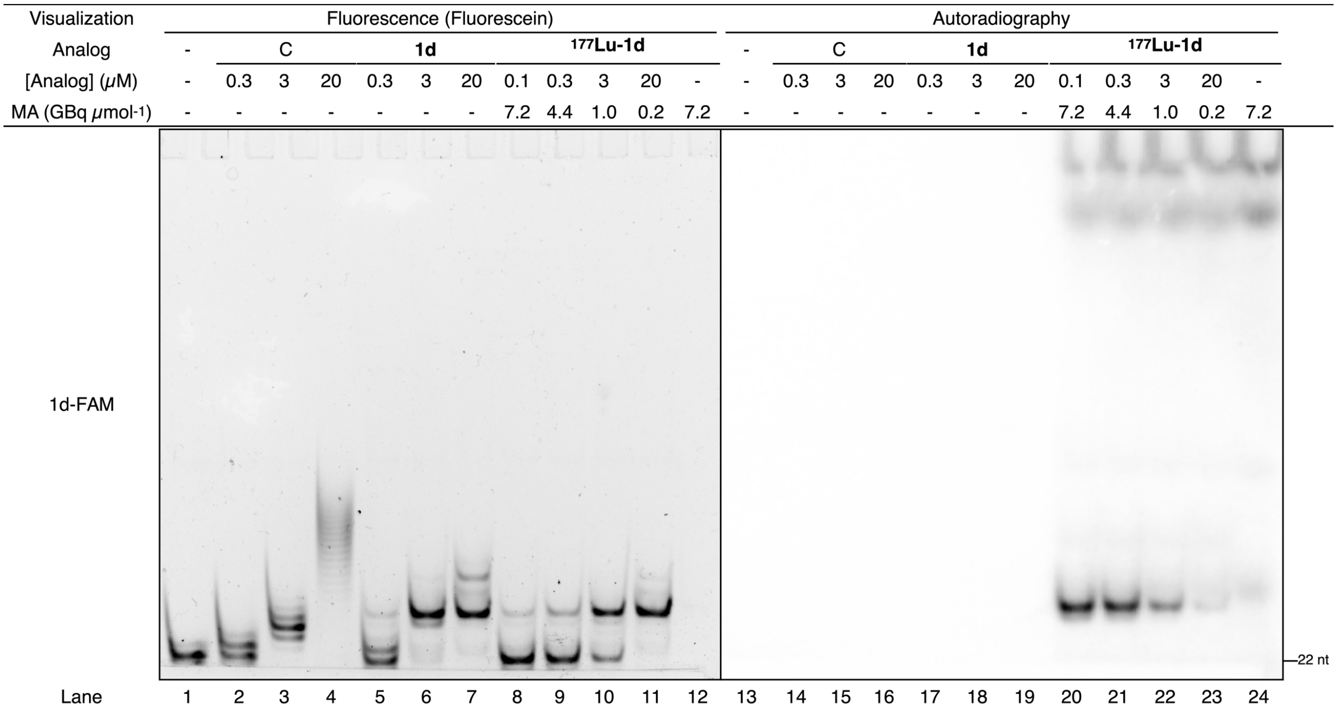

Figure S85. 3′-terminal transferase reaction using 5′-fluorescein-labeled primer **ON-6** (22 nt), and one of dCTP (C), and modified nucleotides **1d**, and **^{177}Lu-1d**. Concentrations and molar activities (for radioactive dNTPs) are indicated in each lane.

Visualization	Fluorescence (Fluorescein)												Autoradiography											
Analog	-	T			**2d**			^{177}**Lu-2d**				-	-	T			**2d**			^{177}**Lu-2d**				-
[Analog] (μM)	-	0.3	3	20	0.3	3	20	0.1	0.3	3	20	-	-	0.3	3	20	0.3	3	20	0.1	0.3	3	20	-
MA (GBq μmol^{-1})	-	-	-	-	-	-	-	7.2	4.4	1.0	0.2	-	-	-	-	-	-	-	-	7.2	4.4	1.0	0.2	-

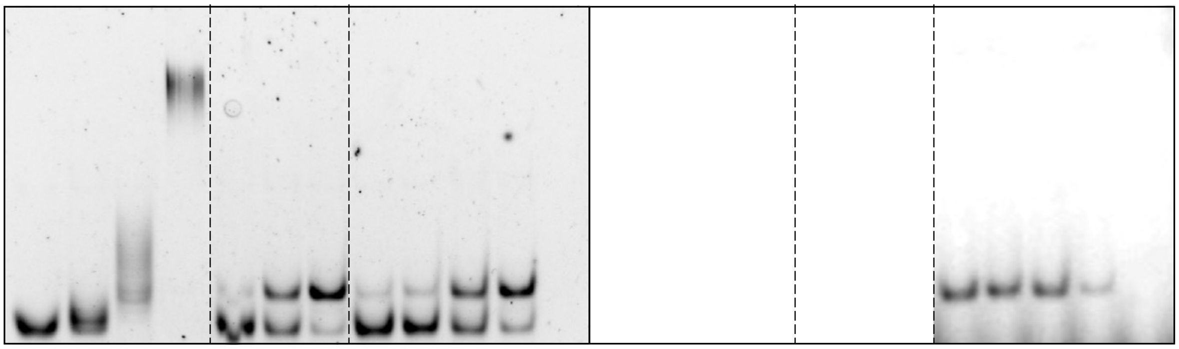

Lane	1	2	3	4	5	6	7	8	9	10	11	12	13	14	15	16	17	18	19	20	21	22	23	24

Figure S86. 3′-terminal transferase reaction using 5′-fluorescein-labeled primer **ON-6** (22 nt), and one of dTTP (T), and modified nucleotides **2d**, and ^{177}**Lu-2d**. Concentrations and molar activities (for radioactive dNTPs) are indicated in each lane.

For the purpose of data integrity, the un-cropped images used for **Figure S86** are provided below.

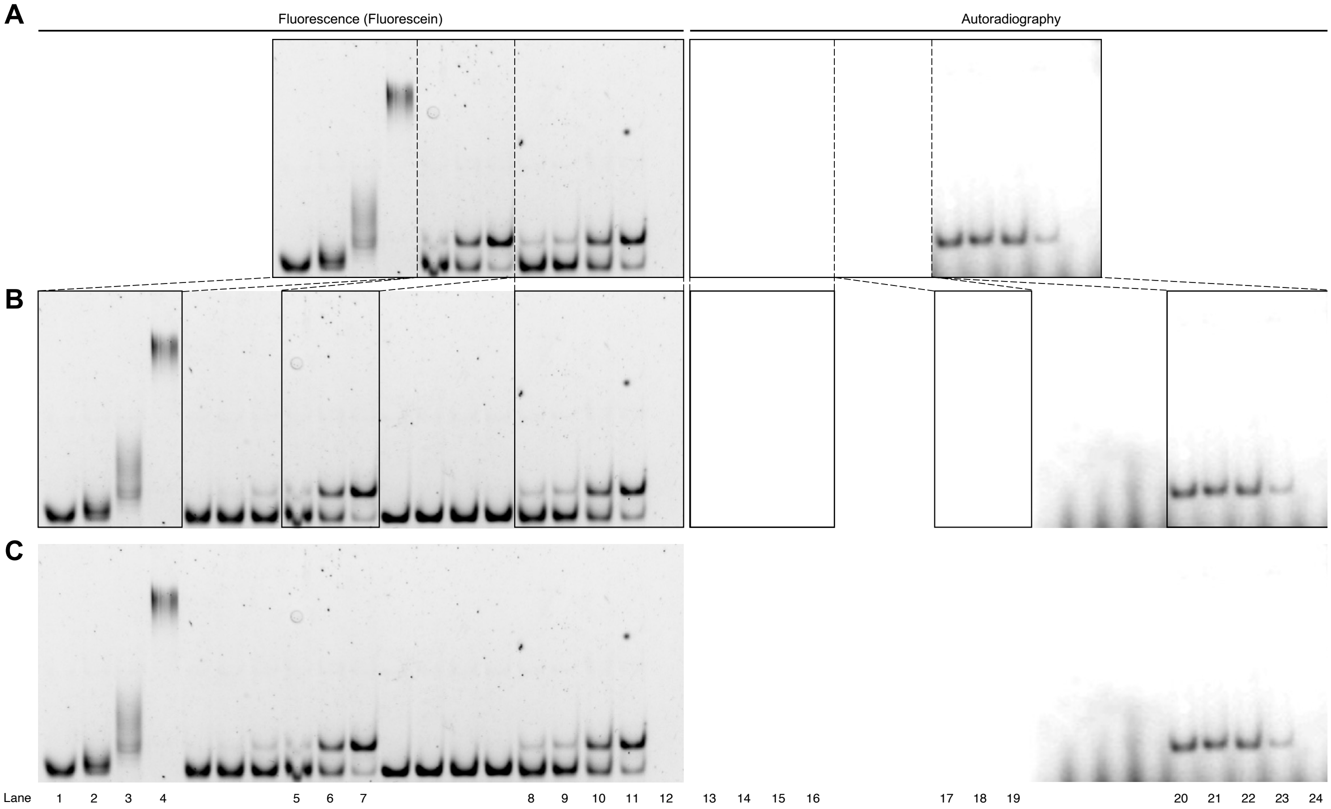

Figure S87. Cropping of original fluorescence and autoradiographic images to produce dataset for 3′-terminal transfer of dTTP (T), modified nucleotides **2d**, or ^{177}**Lu-2d** to primer **ON-6** (**Figure S86**) from original images. Dataset (**A**) was collected by cropping the marked sections (**B**) of the unmarked images (**C**). Lane numbers in **Figure S86** are recapitulated for clarity.

Visualization	Fluorescence (Fluorescein)											Autoradiography										
Analog	-	C			**^{111}In-3a**							-	C			**^{111}In-3a**						
[Analog] (μM)	-	0.3	3	20	0.3	3	20	0.1	0.3	3	20	-	0.3	3	20	0.3	3	20	0.1	0.3	3	20
MA (GBq μmol^{-1})	-	-	-	-	-	-	-	7.2	4.4	1.0	0.2	-	-	-	-	-	-	-	7.2	4.4	1.0	0.2

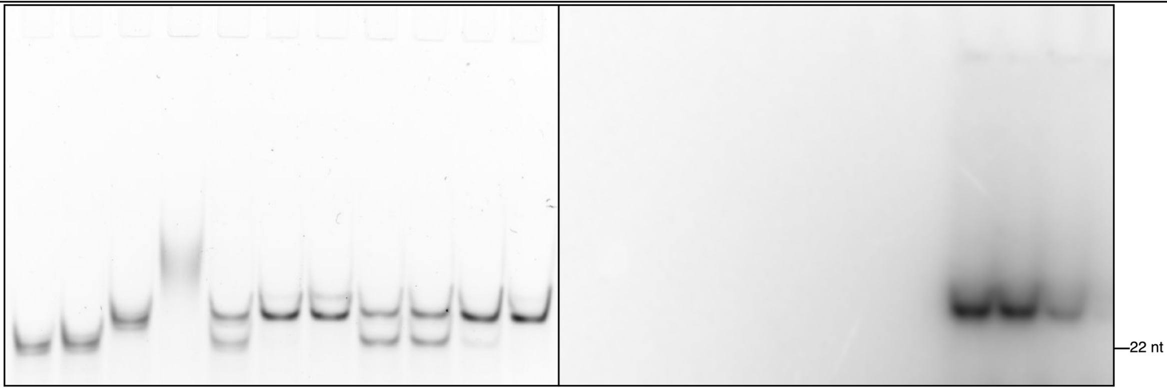

Lane	1	2	3	4	5	6	7	8	9	10	11	12	13	14	15	16	17	18	19	20	21	22

Figure S88. 3′-terminal transferase reaction using 5′-fluorescein-labeled primer **ON-6** (22 nt), and one of dCTP (C), and modified nucleotides **3a**, and **^{111}In-3a**. Concentrations and molar activities (for radioactive dNTPs) are indicated in each lane.

Visualization	Fluorescence (Fluorescein)											Autoradiography										
Analog	-	T			**^{111}In-4a**							-	T			**^{111}In-4a**						
[Analog] (μM)	-	0.3	3	20	0.3	3	20	0.1	0.3	3	20	-	0.3	3	20	0.3	3	20	0.1	0.3	3	20
MA (GBq μmol^{-1})	-	-	-	-	-	-	-	7.2	4.4	1.0	0.2	-	-	-	-	-	-	-	7.2	4.4	1.0	0.2

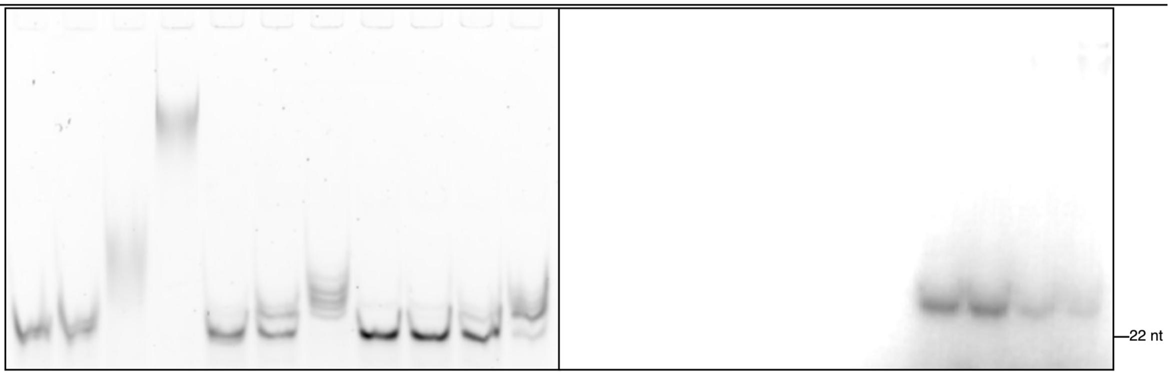

Lane	1	2	3	4	5	6	7	8	9	10	11	12	13	14	15	16	17	18	19	20	21	22

Figure S89. 3′-terminal transferase reaction using 5′-fluorescein-labeled primer **ON-6** (22 nt), and one of dTTP (T), and modified nucleotides **4a**, and **^{111}In-4a**. Concentrations and molar activities (for radioactive dNTPs) are indicated in each lane.

Determination of Lu(DOTA) as an Inhibitor of Terminal Deoxynucleotidyl Transferase

This experiment examines Lu(DOTA) complex as an inhibitor of terminal deoxynucleotidyl transferase (TdT). To generate the complex, DOTA (1.0 mM) and $LuCl_3$ (1.0 mM) in NaOAc (1.0 M, pH 4.5) was heated to 95ºC for 15 min to yield the desired complex Lu(DOTA) at 1.0 mM final.

To study the inhibitory effect of the complex, to total reaction volume of 10 µL, primer **ON-5** (100 nM), Terminal Transferase Buffer (1X) **B-3** were subjected to 3′-extension by Terminal Transferase (0.5 U/µL, New England Biolabs M0315L) in dCTP (3 µM), Lu(DOTA) (0.3–300 µM), or water as negative control, for 1 h at 37ºC. The extension products were separated by native PAGE (20%, 500 V, 30 min) electrophoresed in native PAGE buffer **B-8**.

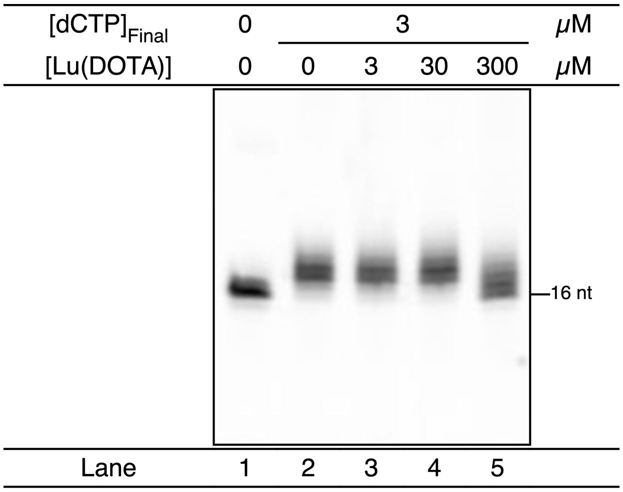

Figure S90. 3′-terminal transferase reaction using 5′-Cy5-labeled primer **ON-5** (16 nt), dCTP, and inhibitor Lu(DOTA). Concentrations are indicated in each lane.

Inductively Coupled Plasma–Mass Spectrometry (ICP-MS)

Inductively coupled plasma–mass spectrometry (ICP-MS) is a sensitive method to quantitatively measure concentrations of selected metal ions. Therefore, preparation of all reagents, samples and solvents must consider accuracy, adventitious contamination and matrix effects.

Preparation of ICP-compliant Reagents, Calibration Standards and Analytes

All parental metal standards were purchased from Millipore Sigma at nominally 1 g/L at ICP-compatible grade. Unless otherwise stated, all calibration standards (1–5 nM) and analytes were prepared in 1% HNO_3 (aq., *v/v*, trace metal grade, Fisher Scientific S010101TFIQ03, shipped in high-density polypropylene bottles) in only plastic equipment or containers (polypropylene: volumetric flasks, micropipette tips, centrifuge tubes, 500 mL bottles, disposable spatulas; polystyrene: weigh boats). The authors would stress that metal or glass vessels must be avoided to prevent adventitious metal ion contamination. Successive dilutions were prepared *via* weighing by difference on an analytical balance, where temperature fluctuations between different dates, typically between 21–23ºC, accounted for density change in HNO_3 (1%).

For all solvents in this section, each solvent (200 mL) was treated with Chelex 100 Resin (Bio-rad, 130 mg) prior to 0.22 µm filtration. $LiClO_4$ (anhydrous) was purchased at ≥ 99.9% purity (Chem-Impex 26479), and sealed under Argon for long term storage, due to its hygroscopic nature. Oligonucleotide containing peaks were collected and diluted in ICP-MS-compliant 1% HNO_3 directly for analysis.

Anionic Exchange HPLC of Oligonucleotides for ICP-MS Analysis Method **M-11**

After primer extension at 10 pmol (20 µL scale, **Figure 4A**) with the corresponding primer, each sample were separated by the HPLC Method **M-11**. Solvent A, 25 mM TRIS (pH 8.5) in 15% MeCN in milliQ-filtered H_2O; solvent B, 25 mM TRIS (pH 8.5), 200 mM $LiClO_4$, in 15% MeCN in milliQ-filtered H_2O; 0.0–7.0 min, 40–61% B; 7.0–15.0 min, 61–74% B; 15.0–15.5 min, 74–75% B; 15.5–18.5 min, 75% B; 18.5–19.0 min, 75–40% B; 19.0–22.0 min, 40% B; flow rate, 0.5 mL/min; column temperature, 19ºC – 21ºC. Column: DNAPac^{TM} PA-100 (ThermoFisher Scientific), BioLC 4 × 250 mm, analytical column (quaternary ammonium), Product No. 043010).

Oligonucleotides HPLC Traces

HPLC Traces of primer **ON-5**, primer-template (**ON-1**) duplex, and heteroduplexes **HD-1–9** are provided in **Figure S91**–**Figure S101**. For naming convention of the heteroduplexes, please consult **Figure 2B**.

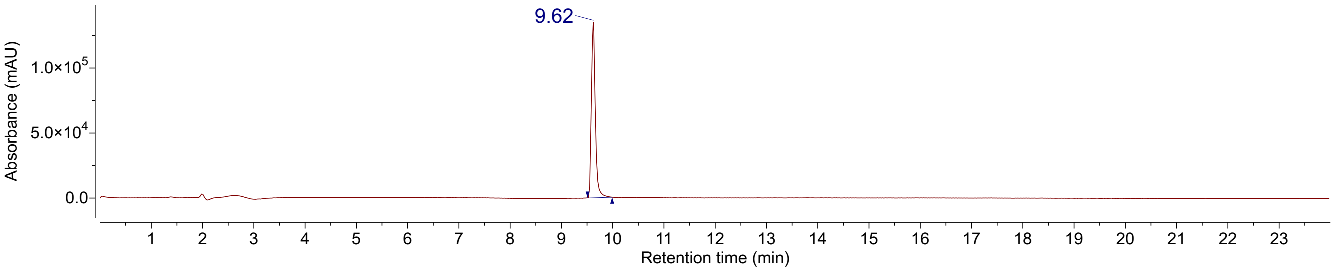

Figure S91. HPLC Trace of primer **ON-5**.

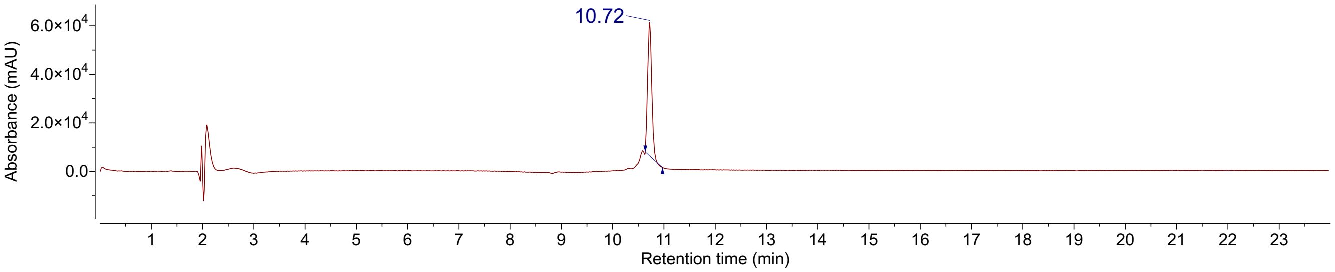

Figure S92. HPLC Trace of primer **ON-5** hybridized to template **ON-1**.

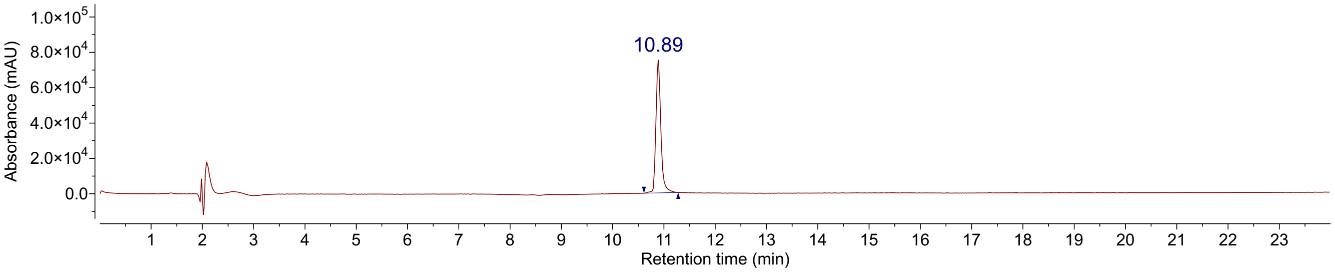

Figure S93. HPLC Trace of heteroduplex **HD-1**.

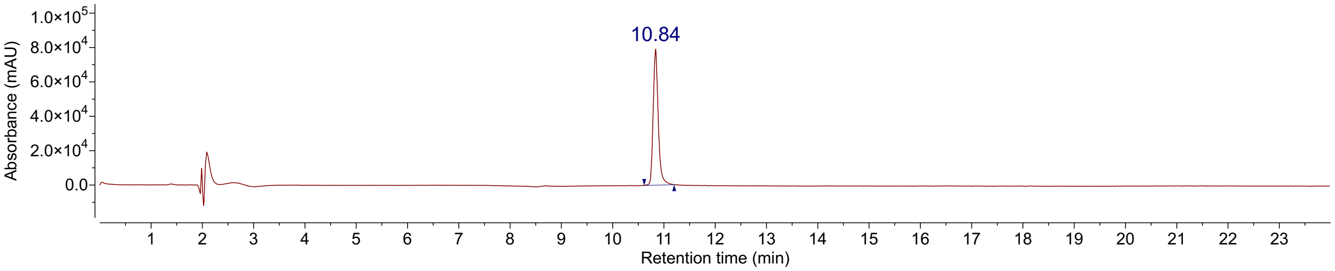

Figure S94. HPLC Trace of heteroduplex **HD-2**.

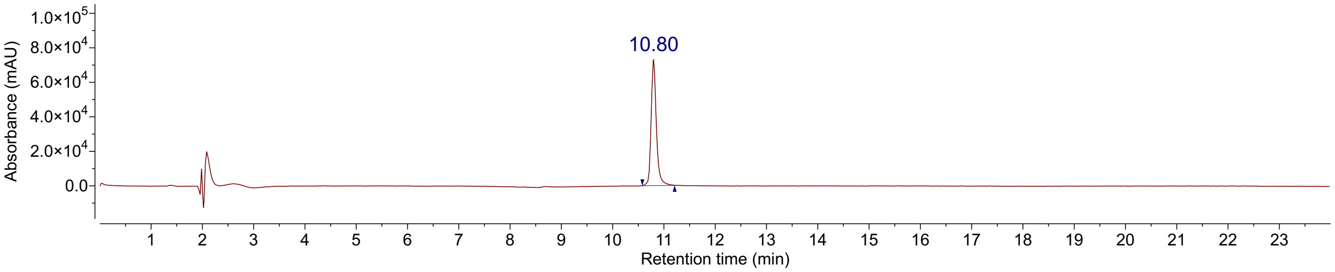

Figure S95. HPLC Trace of heteroduplex **HD-3**.

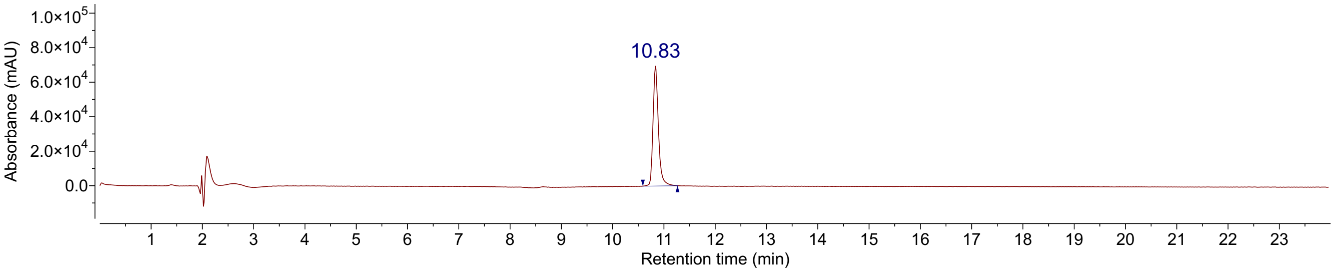

Figure S96. HPLC Trace of heteroduplex **HD-4**.

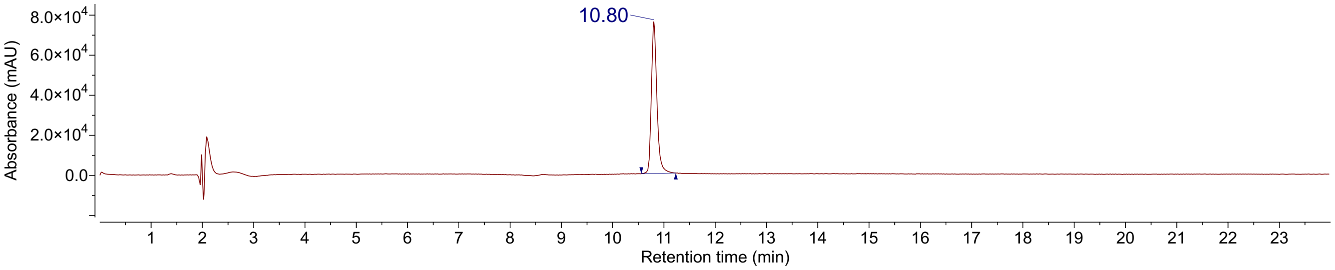

Figure S97. HPLC Trace of heteroduplex **HD-5**.

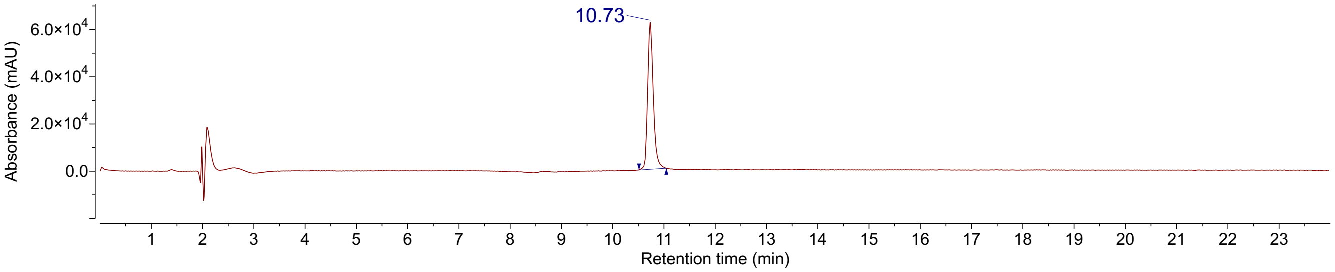

Figure S98. HPLC Trace of heteroduplex **HD-6**.

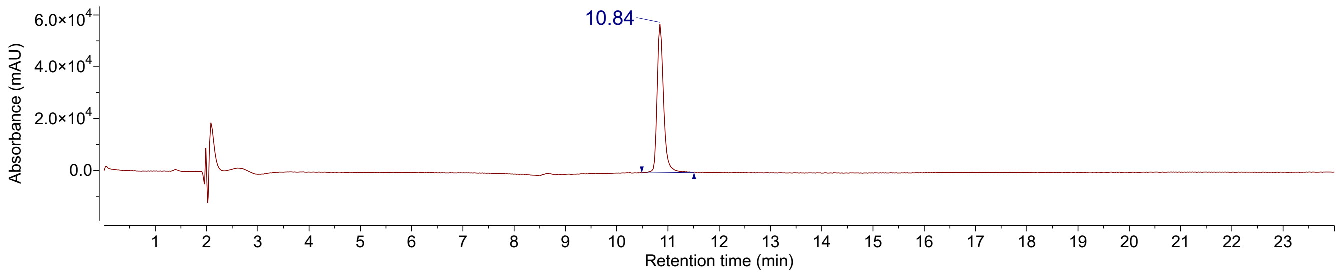

Figure S99. HPLC Trace of heteroduplex **HD-7**.

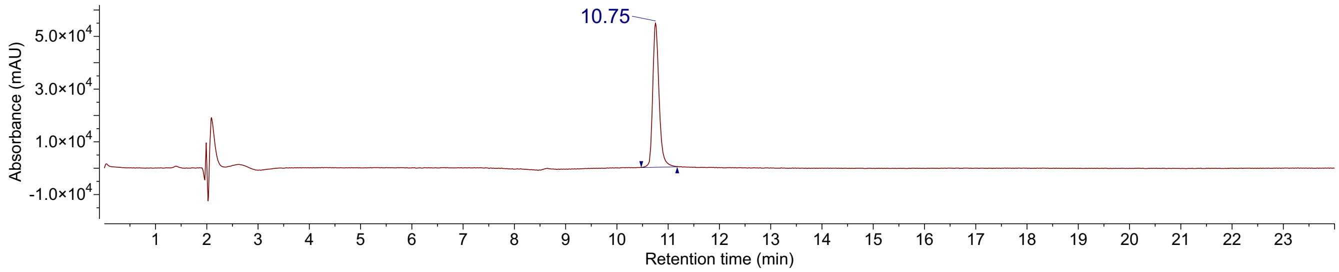

Figure S100. HPLC Trace of heteroduplex **HD-8**.

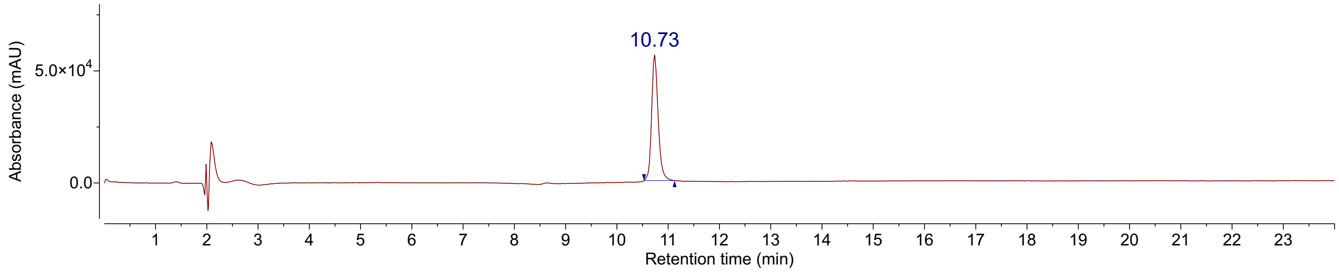

Figure S101. HPLC Trace of heteroduplex **HD-9**.

Sample Introduction and ICP-MS Settings

The HPLC purified analytes (150–170 µL) were diluted in 1% HNO_3 (aq., trace metal grade, Fisher Scientific S010101TFIQ03, 3 mL) with Ga^{3+} (3.0 nM) as internal standard freshly prior to analysis by Agilent 7850 ICP-MS (G8422A, Santa Clara, USA) equipped with an autosampler SPS4 and analysis software MassHunter 5.1 (Agilent G7201D, version D01.01, Build 653.5). Levels of metals detected were reported in counts per second (cps) in **Figure 2D** and **Figure S102**.

Table S7. ICP-MS parameters used.

ICP-MS Setting	Value
Radiofrequency (RF) plasma power	1550 W
Plasma gas	Argon
Nebulizer gas flow	1.0 L min^{-1}
Nebulizer	MicroMist nebulizer
Spray chamber	Peltier-cooled quartz spray chamber
Spray chamber temperature	2 ºC
Sample/skimmer cone	Platinum cones
Collision gas	None
Detection mode	Pulse and Analog
Sample Depth	8 mm

ICP-MS Setting	Value
Energy Discrimination	3.0 V
Injector	1.5 mm injector
Tubing	Peristaltic pump 3-bridged gray/gray tubing
Rinsing Solution	18.2 MΩ cm Type I H_2O
Maintenance Solution	1% HNO_3 in 18.2 MΩ cm Type I H_2O
Replicates per calibration sample	5
Replicates per analyte	5, unless otherwise stated

ICP-MS Source Data

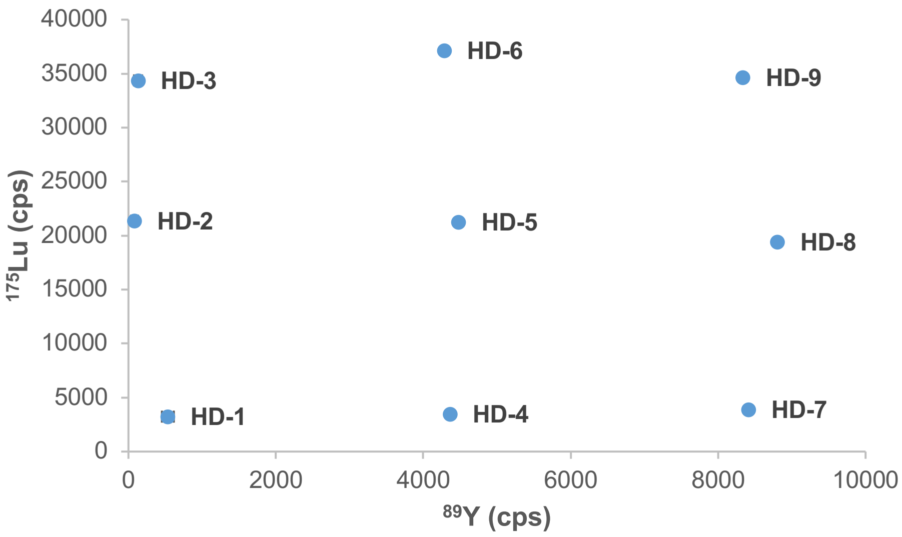

Figure S102. ICP-MS data with error bars, before background subtraction. Error bars are smaller than the marker size.

If a data point is negative after background subtraction, it would be treated as no metal detected.

Table S8. ICP-MS data before background subtraction.

	^{89}Y			^{177}Lu		
Heteroduplex **HD**	Counts (cps)	SD (cps)	N	Counts (cps)	SD (cps)	N
**HD-1**	534.93	80.83	5	3236.49	21.04	5
**HD-2**	82.43	46.14	5	21386.21	4.72	5
**HD-3**	130.46	55.09	5	34374.97	5.18	5
**HD-4**	4363.11	6.10	5	3447.93	5.10	5
**HD-5**	4482.05	11.69	4*	21256.82	6.73	4*
**HD-6**	4284.21	8.14	5	37157.23	6.48	5
**HD-7**	8412.81	7.30	5	3867.38	11.27	5
**HD-8**	8808.00	13.12	4*	19393.79	6.47	4*
**HD-9**	8338.17	3.21	5	34655.98	2.47	5

* An air bubble was introduced during data acquisition, and therefore the affected entry was discarded in data analysis.

References

[8a] A. Roivainen, T. Tolvanen, S. Salomäki, G. Lendvai, I. Velikyan, P. Numminen, M. Välilä, H. Sipilä, M. Bergström, P. Härkönen, H. Lönnberg, B. Långström, *J. Nucl. Med.* **2004**, *45*, 347-355.

[29] J. Schindelin, I. Arganda-Carreras, E. Frise, V. Kaynig, M. Longair, T. Pietzsch, S. Preibisch, C. Rueden, S. Saalfeld, B. Schmid, J.-Y. Tinevez, D. J. White, V. Hartenstein, K. Eliceiri, P. Tomancak, A. Cardona, *Nat. Methods* **2012**, *9*, 676-682.

[30] S. Eissler, M. Kley, D. Bächle, G. Loidl, T. Meier, D. Samson, *J. Pept. Sci.* **2017**, *23*, 757-762.

[31] H. T. Kuo, Z. X. Zhang, C. C. Zhang, H. Merkens, R. Y. Tan, A. Wong, C. F. Uribe, F. Bénard, K. S. Lin, *Theranostics* **2023**, *13*, 4559-4573.
